# Supplementary material for: Can goal-setting for patients with multimorbidity improve outcomes in primary care? Cluster randomised feasibility trial
Source: BMJ Open. 2019 Jun 3;9(6):e025332. doi: 10.1136/bmjopen-2018-025332 (PMC6561432; doi:10.1136/bmjopen-2018-025332)
Supplement: Supplementary data [file bmjopen-2018-025332supp002.pdf]

University of East Anglia

GoalPlan Study:
Goal-setting form
for completion before care plan appointment

Part 1) What are your goals? What is important to you?

Write down what you would really like to do or achieve over the next 6 months, even if you think it may not be related to your health. Think about things that you would like to do in your personal, home, work, and social life—things that you ***need*** to do, ***want*** to do and / or ***enjoy doing***. Then list them in order of priority— starting with 1 for the goal that matters to you most and that you would like to focus on at the moment. To help you, we have put a completed example overleaf.

**What do you really want to achieve over the next 6 months? (your goals)**

Example
• *I want to be able to do more in the garden as it relaxes me*<br>• *I want to lose weight so I can walk more confidently at my daughter's wedding and fit into my favourite outfit again*<br>• *I want to be able to get more exercise as it used to help me sleep well*<br>• *I want to get back to driving so I can visit my friend at his house every week*<br>• *I want to be able to go out with friends once a week again for lunch*

Part 2) Why are these goals important to you?

We now ask you to focus on your goals from Part 1, and to think about why they are important to you. Below is an example of how to find out if a goal you have come up with is important for its own sake, or if it is important because it will help you achieve something else. We ask you to do this exercise with up to three of your goals overleaf.

Part 3) What are the first steps you would like to take towards achieving this goal or goals?

Having identified your most important goals, the final step on this form is to start thing about steps you would like to take to achieve those goals. This is also included in the example below. Please do the same for your goals overleaf.

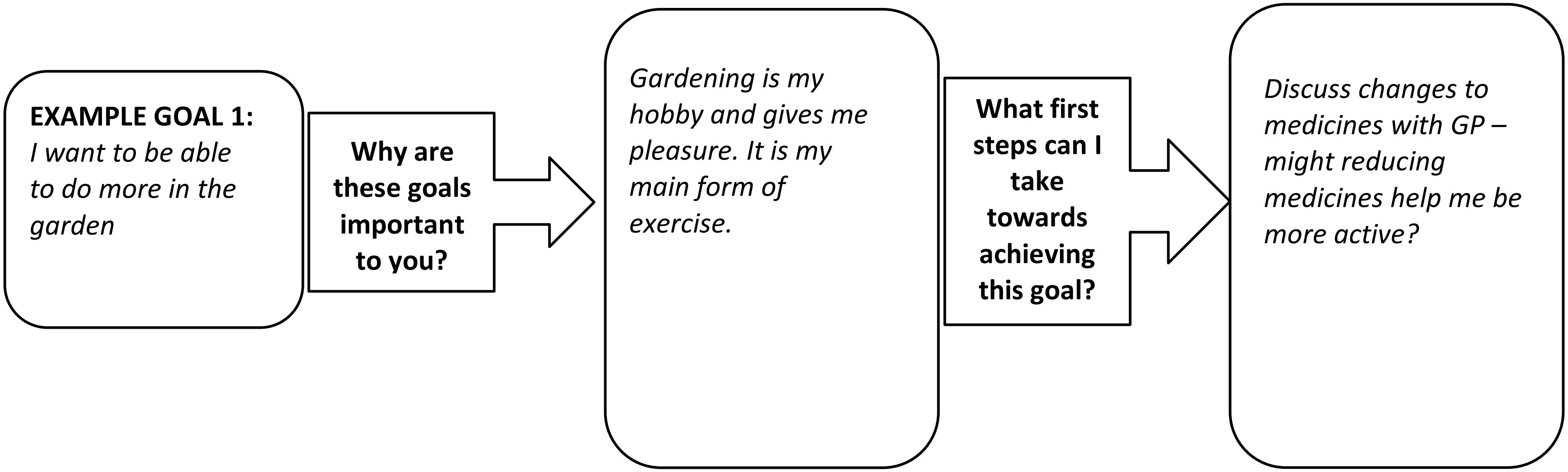

GOAL 1:

Why are these goals important to you?

What first steps can I take towards achieving this goal?

GOAL 2:

Why are these goals important to you?

What first steps can I take towards achieving this goal?

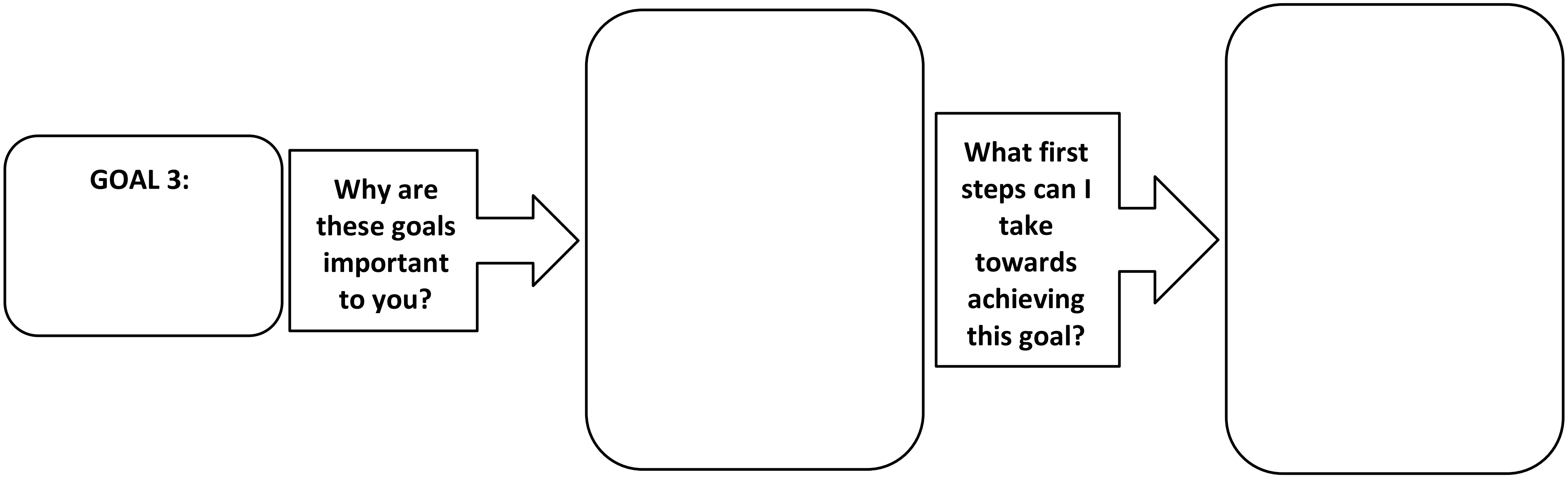

Thank you for completing this goal-setting form. Please take it with you to your care plan appointment. Your GP will discuss it with you and together you will make specific plans to achieve your goals.
