# Supplementary material for: Can goal-setting for patients with multimorbidity improve outcomes in primary care? Cluster randomised feasibility trial
Source: BMJ Open. 2019 Jun 3;9(6):e025332. doi: 10.1136/bmjopen-2018-025332 (PMC6561432; doi:10.1136/bmjopen-2018-025332)
Supplement: Author's manuscript [file bmjopen-2018-025332.draft_revisions.pdf]

BMJ Open

BMJ Open is committed to open peer review. As part of this commitment we make the peer review history of every article we publish publicly available.

When an article is published we post the peer reviewers' comments and the authors' responses online. We also post the versions of the paper that were used during peer review. These are the versions that the peer review comments apply to.

The versions of the paper that follow are the versions that were submitted during the peer review process. They are not the versions of record or the final published versions. They should not be cited or distributed as the published version of this manuscript.

BMJ Open is an open access journal and the full, final, typeset and author-corrected version of record of the manuscript is available on our site with no access controls, subscription charges or pay-per-view fees (http://bmjopen.bmj.com).

If you have any questions on BMJ Open's open peer review process please email editorial.bmjopen@bmj.com

Goal-setting for patients with multimorbidity in primary care: a cluster randomised feasibility trial

Journal:	*BMJ Open*
Manuscript ID	bmjopen-2018-025332
Article Type:	Research
Date Submitted by the Author:	10-Jul-2018
Complete List of Authors:	Ford, John; University of East Anglia, Public Health<br>Lenaghan, E; University of East Anglia<br>Salter, Charlotte; University of East Anglia<br>Turner, David; University of East Anglia, Public Health and Primary Care<br>Shiner, A.; University of East Anglia<br>Clark, Allan; Norwich Medical School<br>Murdoch, Jamie; University of East Anglia, School of Nursing Sciences<br>Green, Carole<br>Lipp, Alistair; NHS England Midlands & East,, West Wing<br>Moseley, Annie<br>Wade, Tom; University of East Anglia<br>Winterburn, Sandra; University of East Anglia<br>Steel, Nicholas; University of East Anglia
Keywords:	PRIMARY CARE, HEALTH SERVICES ADMINISTRATION & MANAGEMENT, SOCIAL MEDICINE

SCHOLARONE™
Manuscripts

Title

Goal-setting for patients with multimorbidity in primary care: a cluster randomised feasibility trial

Authors

John A. Ford, Elizabeth Lenaghan, Charlotte Salter, David A. Turner, Alice Shiner, Allan Clark, Jamie Murdoch, Carole Green, Alistair Lipp, Annie Moseley, Tom Wade, Sandra Winterburn, Nick Steel

Corresponding author

Prof Nick Steel

Norwich Medical School
University of East Anglia
Chancellor's Drive
Norwich
NR4 7TJ
n.steel@uea.ac.uk
01603 591161

Trials registration:
Title: Goal-setting in care planning for people with multimorbidity
Trial ID: ISRCTN13248305
Date registered: 21/12/2016
Link: http://www.isrctn.com/ISRCTN13248305

Protocol: Available at:
http://www.uea.ac.uk/documents/246046/14839702/GoalPlan+Research+Protocol+v1.2+170316.pdf/86549c5b-c4ed-435b-8719-4c3160f9793f

Word count: 4173

Keywords: goals, multimorbidity, primary health care, patient-centred care, randomized controlled trial

For peer review only

For peer review only - http://bmjopen.bmj.com/site/about/guidelines.xhtml

Abstract

Introduction

Goal-setting has been recommended for patients with multimorbidity, but there is little evidence to support use in general practice.

Objective

To assess the feasibility of goal-setting for patients with multimorbidity with a view to undertaking a definitive trial.

Design and setting

Cluster-randomised controlled feasibility trial of goal-setting compared to usual care planning in six general practices. Blinding was not possible.

Participants

Adults, at risk of unplanned admission, diagnosed with ≥2 chronic health problems and eligible for a care planning consultation.

Interventions

In the three goal-setting practices, General Practitioners (GPs) underwent training and patients were asked to consider goals before an initial goal-setting consultation and six month follow-up consultation. The control group received usual care planning.

Outcome measures

Standard scales for health-related quality of life (EQ5D), capability (ICECAP-O) and care for chronic conditions (PACIC) and health care use at six months. All consultations were video-recorded, and focus groups held with participating GPs and patients.

Results

Fifty-two participants were recruited (response rate 9%) and full follow-up data was available for 41. Mean age was 78.7 years (SD 9.2) and median prescribed medications 12 (IQR 9-17), 46% were women, and 69% had slight cognitive impairment. Participants in the goal-setting group set between one and three goals on a wide range of subjects. The goal-setting group had higher scores for shared decision-making compared to the usual care planning group, but not statistically significantly. There was no significant difference in EQ5D or PACIC between groups, and ICECAP-O was slightly higher in usual care planning. Patient participants found goal-setting acceptable and would have liked more frequent follow-up. GPs unanimously liked goal-setting, felt it delivered more patient-centred care and highlighted the importance of training.

Conclusions

This goal-setting intervention was feasible to deliver in general practice. A larger, definitive study is needed to test its effectiveness.

For peer review only - http://bmjopen.bmj.com/site/about/guidelines.xhtml

Article summary

- Goal-setting is recommended for patients with multimorbidity, but there is currently little evidence to support its use in primary care.
- Here we present a feasibility study of goal-setting in primary care for patients with multimorbidity.
- We found that goal-setting was acceptable to patients and unanimously supported by GPs who felt it helped deliver more patient-centred care.
- A larger, definitive trial is needed to test the effectiveness and cost-effectiveness of goal-setting.

For peer review only

For peer review only - http://bmjopen.bmj.com/site/about/guidelines.xhtml

Introduction

The rising number of long-term conditions and prescribed medications has increased the burden of treatment for patients [1]. People with multimorbidity (defined as two or more chronic conditions [1]) tend to have a lower quality of life and worse health than those with single conditions [2]. Medical outcomes that work well for relatively healthy patients (e.g. blood pressure control, or disease-free survival) may be inappropriate for patients with multimorbidity or severe disability [3 4], and the use of current single-disease guidelines in this group can encourage harmful polypharmacy with resulting drug-drug and drug-disease interactions [5].

Personalised care planning is 'a conversation in which patients and clinicians agree goals and actions for managing the patient's conditions' [6]. Goal-setting, the sharing of realistic treatment goals by physicians and patients, is core to the theory and effective practice of care planning and is particularly important for patients with multimorbidity [6 7]. Patient engagement and co-production with goal-setting as part of a care planning process is currently recommended by NICE for patients with multimorbidity [8]. Despite this recommendation, there is little evidence to support the use of goal-setting between general practitioners and patients, and it is rarely used in primary care [6 9 10]. A recent systematic review highlighted the lack of evidence exploring 'the effects of personalised care planning on goal-attainment, especially patient's personal goals as opposed to goals determined by clinicians or researchers' [11]. Our goal-setting intervention was designed within the context of a nationally funded recommendation that the top 2% of patients at risk of admission should have a care plan [12]. The extra funding for this care planning has now been removed, but the recommendation for GPs to discuss care plans with patients with complex needs remains.

We aimed to assess the feasibility of goal-setting for patients with multimorbidity, at high risk of hospital admission and eligible for a care planning consultation, with a view to undertaking a future definitive randomised controlled trial. Our objectives were to assess participant recruitment and retention, the acceptability of a goal-setting intervention to patients and GPs, the training needs of GPs, the content of usual care planning consultations, goal-setting and the feasibility of collecting relevant outcome measures.

For peer review only - http://bmjopen.bmj.com/site/about/guidelines.xhtml

Methods

Setting

We undertook a parallel group, cluster randomised controlled feasibility trial of goal-setting compared to usual care in six general practices in Norfolk and Suffolk with six months follow-up. There were no significant changes to the protocol, which is publicly available [13]. Research ethics approval was obtained from the NHS Research Ethics Committee (16/EM/0411). Participants were recruited between April and May 2017 and follow-up completed in February 2018.

Eligibility criteria

General practices were recruited via the East of England Clinical Research Network on a first-come first-served basis. Patients were eligible if they were aged 18 or over, identified as in the top 2% for risk of unplanned admission as part of the 'Avoiding Unplanned Admissions' Enhanced Service [12], eligible for a new or review care planning consultation during the data collection period and diagnosed with at least two of 40 morbidities in Barnett's analysis of multimorbidity [1]. Patients were excluded if they were deemed to be unable to participate in goal-setting in the GP's professional opinion (e.g. advanced dementia or acute psychosis), in receipt of care planning consultation in previous three months or required translation services to communicate verbally.

Recruitment

Each practice undertook a search of their electronic patient register, according to the eligibility criteria, and sent a letter of invitation to 100 identified patients, with the intention of recruiting 10 patients per practice. Patient participants were randomly selected with oversampling in postcodes in the lowest Index of Multiple Deprivation quartile [14] to increase the likelihood of recruiting participants from a range of socio-economic groups. The protocol allowed GPs to opportunistically invite patients they thought might be interested, however no patients were recruited through this process. Interested patients were visited by a study researcher to discuss the study and obtain written informed consent.

Randomisation

The Norwich Clinical Trials Unit independently randomised three practices to goal-setting and three to usual care planning, by simple block randomisation using a 1:1 ratio and sealed opaque envelopes. Practices were randomised after at least 10 expressions of interest were received from patients. It was not possible to blind participants, health professionals or researchers due to the nature of the intervention.

Intervention

GPs from practices allocated to goal-setting received training in the form of a three hour experiential workshop, led by senior consultation skills tutors (CS and SW) and a GP with experience in communication skills training (AS). The training model we developed built on both the work of Elwyn and colleagues on shared decision making [15 16] and the Calgary Cambridge Guide [17]. It included an introduction to the study, interactive skill spotting and role-play. GPs were trained in groups of three and provided in advance with a detailed handbook. Usual care planning group GPs received no training and were asked to undertake a care planning consultation as they would usually do in routine clinical practice.

For peer review only - http://bmjopen.bmj.com/site/about/guidelines.xhtml

For peer review only

Patient participants in the goal-setting arm were given a face-to-face explanation of goal-setting lasting approximately 15 minutes and a patient held goal-setting sheet (PGS) by the study researcher (EL) with three questions to consider prior to their consultation. The questions were: what's important to you and what would you like to achieve over the next 6 months; why is it important to you; and, what are the first steps you would like to take towards achieving this goal or goals? (See Supplementary Appendix 1). During the initial goal-setting consultation GPs, in partnership with participants, documented the goals which had been agreed. Participants in both the usual care planning and the goal-setting groups had an initial consultation which lasted about 20 minutes, but only patients in the goal-setting arm were invited back for a follow-up consultation after 6 months to discuss their goal attainment.

Data

Quantitative and qualitative data were used to meet our feasibility study objectives. Key data on recruitment and retention were collected throughout the trial. Postcode IMD scores for participants and practices were recorded. Data collected from patients during a researcher visit at baseline and six months assessed health-related quality of life and capability (EQ-5D-5L [18], ICECAP-O [19]), cognition (general practitioner assessment of cognition scale [20]) and patient centred care (patient assessment of care for chronic conditions scale [21]). Data collected from the electronic patient record included demographics (baseline only), medications on repeat prescription, diagnoses, Quality and Outcomes Framework information [22] and primary and secondary care use.

GPs and patient participants were asked to complete an assessment of shared decision making during each consultation using the CollaboRATE scale [23] for patients and dyadic OPTION scale [24] for GPs. GPs and patients in the goal-setting group were asked to discuss and complete a goal attainment scaling (GAS-Light) questionnaire [25] (See Supplementary Appendix 2) at the second consultation. Goal attainment was scored using the following system: -1 = worse than expected, 0 = no change, 1 = partially attained, 2 = as expected, 3 = a little more and 4 = a lot more than expected.

All initial consultations were video or audio recorded and transcribed. Three team members scored the consultations using the observer OPTION measure to assess shared decision making [26]. One focus group was held with patients and one with GPs from the goal-setting group at the end of the six month follow-up period to discuss perspectives, experiences and overall acceptability of the goal-setting intervention. Both focus groups lasted about 90 minutes, were held at the university, guided by a topic guide, audio-recorded and transcribed. Participants unable to attend the focus group were interviewed by phone using the same topic guide.

Statistical analysis

The recruitment rate was summarised by practice and by randomisation group. Demographic variables were compared for those recruited and those not recruited. The characteristics of baseline consultations were summarised both by practice and by intervention group. Key characteristics were compared using a linear mixed model with practice included as a random effect.

The change in outcome measures from baseline to follow-up was summarised using descriptive statistics by randomisation group. Exploratory analysis estimated the difference between randomisation groups using a linear mixed model with practice included as a random effect. This would allow the estimation of potential differences in a full scale trial. The intra cluster correlation coefficient was estimated for each outcome, however great care should be taken in the interpretation of these due to the small number of clusters [27].

For peer review only - http://bmjopen.bmj.com/site/about/guidelines.xhtml

Health economic evaluation

Data were collected on resource use from an NHS perspective to test data collection processes and to inform a future health economic evaluation estimating quality adjusted life years (QALYs). A record was kept of resources required to provide GP training, as well as the length of initial and follow-up goal-setting consultations. Additional health care resource use was extracted from electronic health records by practices supported by a study researcher (EL) for the 6-months prior to randomisation and from randomisation to follow-up. Health care use was collected for: day-case and inpatient hospital admissions; outpatient visits; accident and emergency visits (A&E); consultations at the GP practice (GP, practice nurse, health care assistant, nurse practitioners); and other contacts, such as district nursing, allied health professional contacts, ambulance call outs, and specialist nursing contacts.

Resource use was costed using the NHS reference costs [28] for secondary care and a published source for primary care contacts [29]. NHS reference costs were used to estimate a weighted average cost for day cases, non-elective short stay, non-elective long stay, and elective admissions. For longer stays, additional days were costed using a weighted average of all excess bed day costs. For the first and second GP consultations in the goal-setting group, we had data on length of consultation and setting. The cost of providing training was estimated from a description given by the study researcher of duration and required staff. The cost of academic staff time was estimated using University pay scales (including employer's national insurance and superannuation payments). As the training would have relevance beyond the duration of the study, we estimated a useful life of 3 years and calculated an annual equivalent cost[30]. All costs are in 2015/16 UK pounds sterling. As the duration of the study was 6-months, we did not discount costs and benefits. As the study size was very small with great variability in estimates of cost and effect, we did not estimate formal cost-effectiveness.

Review of video-recorded consultations

The usual care planning and goal-setting consultations were compared by the research team (CS, EL, AS, JM and RH) to measure duration and explore the content and methodological implications for a future study. An in-depth analysis of the consultations using a conversation analytic informed approach [31] to explore sequential patterns, verbal and non-verbal behaviours and turn taking will be reported elsewhere.

Focus groups

A thematic framework-based analysis was used to analyse the focus groups [32]. This enabled a structured summarised overview of the acceptability of the goal-setting intervention to patients and GPs and an understanding of possible future improvements to the goal-setting intervention, training and trial design.

Patient and Public Involvement (PPI)

Two PPI representatives contributed to the design of the research as co-applicants on the initial application for funding (AM and HS) and steering group membership (AM and CG). Furthermore PPI members contributed to the analysis and interpretation of the results, with one PPI representative reviewing and scoring video consultations (RH) and a further two reviewing a selection of video consultation transcripts (AM and CG). Two PPI members reviewed and commented on the manuscript and are co-authors (AM and CG).

For peer review only - http://bmjopen.bmj.com/site/about/guidelines.xhtml

Results

Recruitment and retention

Sixty general practices were invited with seven expressing interest and six being recruited (Figure 1). Across the six practices (Table 1), 550 patients met the eligibility criteria and were invited. In total, 52 patients were recruited (9.5%) with 24 belonging to practices randomised to goal-setting and 28 to practices in the usual care planning group. There was little variation in age and deprivation between those who participated and those who did not, but slightly fewer women (46%) than men took part (Supplementary Table 1). Two participants in the goal-setting group and five in the usual care planning group did not receive the initial consultation because they declined to attend, were unavailable or withdrew consent. Four participants in the goal-setting group did not receive the follow-up consultation because of ill health or death. Data collected directly from participants were available for 18 participants in the goal-setting group and 23 in the usual care planning group. Participant data collected from practices were available for 23 participants in the goal-setting group and 28 in the usual care planning group. Recruitment started in

Baseline characteristics of practices and participants

The usual care planning practices were in more urbanised areas with larger practice populations and more female GPs participating compared to goal-setting practices (Table 2). The goal-setting group, compared to usual care planning, had more patient participants who were female (54% compared to 29%), older (80 years old compared to 77), with a higher number of health problems (5 compared to 4) and medications (13.0 compared to 11.5), but similar quality of life (Table 3). The usual care planning group had participants spread across all four IMD quartiles, whereas the goal-setting group had participants in only the second and third quartiles. Table 1 shows that there was variation in mean age (range 69.5 to 85.8 years old), proportion of females (range 25% to 73%), number of medications (range 10.0 to 15.5) and number of health problems (range 3.0 to 7.5) across participating practices.

Consultation findings

The mean initial consultation time in the goal-setting group was 23.0 minutes and in the usual care planning 19.2 minutes (Table 4). Patients spoke more in the goal-setting group initial consultation (mean GP:patient word count ratio (WCR) 1.35) compared to usual care planning (WCR 1.52). Dyadic OPTION scores for GPs perceptions of shared decision making were higher, but not statistically significantly, in the goal-setting group compared to the usual care planning group, but collaboRATE scores were similar. Observer OPTION scores proved less informative because of large variation and inconsistency in scoring between the three research team members (data not presented).

Most patients set two or three goals (Table 5), with Practice 1 setting on average one more goal than Practice 3. The commonest type of goals were related to management of chronic conditions, walking, maintaining social and leisure interests or weight management (Table 6). Forty-two of the 50 goals were scored with a mean attainment score per patient of 1.45 (1= partially attained and 2= as expected) with 'partially attained' being the commonest outcome.

Based on review of the initial and follow-up goal-setting consultations, we found variation in the extent to which patients were prepared for the goal-setting consultation, either in terms of considering goals or completing the suggested goal-setting sheets. Some patients quickly understood the concept of goal-setting and were able to identify suitable goals, whereas others found this process more difficult. The term 'care planning' was not mentioned in any consultation.

For peer review only - http://bmjopen.bmj.com/site/about/guidelines.xhtml

In the usual care planning arm, goals were rarely mentioned. However, whilst four usual-care GPs followed the care planning template recommended within the Enhanced Service [12], one GP appeared to treat it as a normal consultation (i.e. problem focused) and another focused entirely on end of life issues. The consultations were generally doctor-led and, despite covering a wide range of topics from mobility aids to end of life wishes, were not structured to plan care according to the patient's own goals and concerns.

Outcome measures

There was no statistically significant difference between goal-setting and usual care planning from baseline to follow-up in PACIC score, health-related quality of life as measured by EQ5D, number of medications or GPCOG score (Table 7). Capability as measured by ICECAP-O at six months, improved slightly more in the usual care planning group than in the goal-setting group (mean difference between groups 0.12, 95% CI 0.02 to 0.22).

There was considerable variation in health care use in the 6 months prior to randomisation and 6 months follow-up as was expected with a small study (Table 8). Most health care contact increased in both the usual care planning and goal-setting groups, but district nurse contacts increased and inpatient admissions decreased only in the goal-setting group. Quality and Outcomes Framework data were collected at baseline and follow-up, but the results were uninformative due to low numbers and low variability (Supplementary Table 2). There was one death in the goal-setting group due to cancer, which was judged to be unrelated to the intervention. The estimated cost of the goal-setting was £147 per patient, of which £95 related to costs of providing initial and follow-up GP consultations, and £43 related to the cost of GP training. There was a small cost for the study researcher to explain goal-setting. A mean cost of £50 per patient was incurred in the usual care planning group for the initial consultation. The single largest cost for the 6-months prior to recruitment and the 6-months of follow-up was inpatient stays (Table 8). However, significant costs occurred outside the hospital setting, for example in general practice contacts and district nurse services. Costs were very heterogeneous, as would be expected.

Acceptability and methodological implications for a definitive trial from focus group data

All six patient participants attending the focus group reported positive experiences and views of the intervention, particularly regarding the different emphasis of the consultation. Participants spoke of using goals to plan and focus their lives, helping them to direct their energy onto something that really mattered to them. The patient below encapsulated this, saying:

> *"[Goal-setting] gives he or she a much better understanding of particularly what is worrying you, what your aims are, the things that you miss being able to do and to be able to actually explain it where [GPs] have time, because very often the GPs, you know, you've only got ten minutes. But with these consultations, you're actually able to talk to a doctor, as you would indeed a friend almost" (Patient 107)*

There was unanimous support for the intervention amongst the four GPs who attended the GP focus group and one GP who was interviewed by phone. GPs described the goal-setting consultations as *more patient-centred and reflected on its 'therapeutic powers'* s (GP10) compared to day-to-day general practice that GPs felt could be dominated by '*box-ticking*' and '*target driven*' (GP018) medicine. Extract 1 below illustrates this sentiment:

> *"I felt almost as if I was trying to put on a different hat, you know, trying not to constantly interrupt them or to sort of sway them in any way, I was trying to give them the opportunity*

For peer review only - http://bmjopen.bmj.com/site/about/guidelines.xhtml

to just say what they wanted to say and set any goal that they wanted to and I, and it made me reflect on actually what I do during the day to day when I've got ten minutes with a patient and I'm very aware of the sort of pressure of, oh I've got to do a medication review and I've got to do this and oh no, their cholesterol's now 7 and oh gosh I've, have my colleagues already spoke to them about this and are they aware of X, Y and Z and actually it was quite nice in a way just take a step back and think, um I don't have to do that with this consultation, let's see what happens when the patient has more control over it" (GP025)

Patient participants spoke positively about the baseline researcher visit because it helped them understand the study and encouraged them to reflect on what was important. However, when discussing wider implementation across the health service, participants acknowledged that a home visit for each patient may be too costly and alternative provision would be acceptable to most people.

Continuity of care was a concern for patient participants. While one person was disappointed not to see their own GP, three were positive about consulting with a different doctor, especially if it was difficult to see their usual GP. However, participants spoke of wanting more follow-up and consistency amongst the health care team in relation to their goals in the future; some participants felt there was a disconnection between the activity of goal setting and their subsequent treatment by staff within the practice.

GPs stated that the experiential work, especially role play and skill spotting, was the most useful aspect of training. When discussing delivering training at scale, GPs felt e-training with opportunities to watch '*other people role-play*', would fit in with their busy schedules. In addition, multiple shorter e-training modules, using a '*step-by-step*' approach (GP014) that contributed to continuing professional development, would be attractive to GPs when implementing the intervention more widely.

For peer review only - http://bmjopen.bmj.com/site/about/guidelines.xhtml

Discussion

Summary of principal finding

We found that the overall process of setting goals, reviewing them in a GP consultation and follow-up over six months was acceptable to patients and unanimously supported by participating GPs. Recruitment and retention of practices and patients was achieved. A wide range of goals were set and, as expected with a feasibility study, there were no statistically significant differences in the main outcomes, except for the ICECAP-O score which was slightly higher in the usual care planning group. The qualitative findings were that goal-setting helped patients and GPs focus on what was important and supported GPs to deliver more patient-centred care. Patient preparedness, continuity of care and being able to deliver training at scale were highlighted as important future considerations.

Strengths and weakness of the study

Whilst recruitment was sufficient, there was under-recruitment in one practice (Practice 3 recruited four out of a target of ten), perhaps because of the small practice size. Those GPs and patients choosing to take part are also likely to be a self-selecting group who are willing to take part in research and are open to goal-setting. An intention-to-treat analysis was undertaken to reduce the impact of protocol violations (e.g. patients not receiving the pre-specified intervention) and attrition bias. Data on the number of health problems were not sufficiently robust for analysis because they were extracted from practice records using different processes. Asking GPs in the non-intervention group to undertake a video-recorded usual care planning consultation is likely to have altered practice compared to what would have happened within the enhanced service.

Implications for a definitive trial

Goal-setting training was important, and the small group face-to-face training with role play could be delivered at scale to the whole GP workforce using online e-learning, based on material collected during this feasibility study.

Both EQ-5D-5L and the ICECAP-O could be used any future economic evaluation. While EQ5D values did not differ between groups, ICECAP-O improved more in the usual care planning participants. This may be a spurious finding due to the small number of participants in the goal-setting arm, or because they were slightly older with more health problems and therefore more likely to deteriorate during follow-up.

Quality and Outcomes Framework data did not prove useful because of the small numbers and low variation. The observer OPTION scoring, initially developed within a rehabilitation context, had poor consistency between researchers and therefore was less useful. The readiness of patients to undertake goal-setting appeared to be important.

The disconnection reported by patients when goals they had set were not considered in future health care contact suggests that more effort is needed in future studies to ensure that goals were communicated with the rest of the health care team. Additional follow-up with their GP may support continuity, which has recently been associated with lower mortality [33].

Comparison with other studies

A Cochrane review, published in 2015, assessed the effects of personalised care planning (defined as goal-setting and action planning), for adults with long term health conditions compared to usual care [6]. Whilst 19 RCTs were included, all except for one focused on single conditions. The one multiple

For peer review only - http://bmjopen.bmj.com/site/about/guidelines.xhtml

condition study included patients who had high health care use and focused on care planning, with goal-setting as part of the process, across the wider health care system to reduce unplanned admissions [34]. The authors found an increase in quality of life (measured by SF36) in the intervention compared to control, however with 50% of participants lost to follow-up and intention to treat not undertaken, there is a possibility of a lost to follow-up bias in favour of the intervention. Our study has focused on goal-setting specifically in primary care.

A systematic review of randomised and non-randomised studies, published in 2017, looked at collaborative goal-setting or health priority setting for elderly people with a chronic condition or multimorbidity [11]. Based on eight included studies, the authors found that in four intervention studies multifactorial approaches had positive effects on the application of goal-setting or care planning, but the review did not assess the impact on health outcomes or quality of life. The authors conclude that future research is needed to determine the "mix of essential elements within a multifactorial intervention to provide recommendations on daily practice". Our study helps to answer this question by identifying some key requirements of goal-setting in primary care as described above.

Implications for clinical practice

This was a feasibility study and the main implications are for the design of a subsequent definitive trial. Goal-setting consultations increased opportunities for GPs to deliver more patient-centred care for patients with complex health conditions, with no longer consultations than in the comparator care planning group. Care planning consultations are likely to remain an important part of the work of GPs, particularly with frail patients [35], and an ageing population with multimorbidity. Our study suggests that goal-setting can be a valuable tool for GPs to carry out patient-centred assessments.

For peer review only - http://bmjopen.bmj.com/site/about/guidelines.xhtml

Competing interests: None declared.

Author contributions: NS, JF, CS and AS conceived the idea. All authors contributed to the design of the study. LL led the data collection. CS, JM and AS led the analysis of the qualitative data. AC undertook the statistical analysis. DT undertook the economic analysis. All authors contributed to the interpretation of the results. JF drafted the initial manuscript. All authors revised the manuscript and approved the final version. NS is the guarantor.

Acknowledgements: We thank Becky Harmston for reviewing the video-consultations from the patient and public involvement perspective. We thank Clara Yates and Gosia Majsak-Newman at the Norfolk and Suffolk Primary and Community Care Research Office for their support. Finally we thank the patients and staff who took part in the study.

Funding statement: This work was supported by the National Institute for Health Research - Research for Patient Benefit programme grant number PB-PG-0215-36079. This article/paper/report presents independent research funded by the National Institute for Health Research (NIHR). The views expressed are those of the author(s) and not necessarily those of the NHS, the NIHR or the Department of Health. The funder did not have any role in the design, collection, analysis or interpretation of data or in writing the manuscript.

Data sharing: Requests for data sharing from this trial should be directed to the corresponding author.

For peer review only

References

1. Barnett K, Mercer SW, Norbury M, Watt G, Wyke S, Guthrie B. Epidemiology of multimorbidity and implications for health care, research, and medical education: a cross-sectional study. *Lancet* 2012;**380**(9836):37-43 doi: 10.1016/s0140-6736(12)60240-2.
2. Fortin M, Lapointe L, Hudon C, Vanasse A, Ntetu AL, Maltais D. Multimorbidity and quality of life in primary care: a systematic review. *Health Qual Life Outcomes* 2004;**2**:51-51 doi: 10.1186/1477-7525-2-51.
3. Wilkie P. Really putting patients first: ensuring significant involvement for patients in healthcare decision making. *Br J Gen Pract* 2015;**65**(632):108-09 doi: 10.3399/bjgp15X683821.
4. Reuben DB, Tinetti ME. Goal-Oriented Patient Care — An Alternative Health Outcomes Paradigm. *N Eng J Med* 2012;**366**(9):777-79 doi: 10.1056/NEJMp1113631.
5. Guthrie B, Payne K, Alderson P, McMurdo MET, Mercer SW. Adapting clinical guidelines to take account of multimorbidity. *BMJ* 2012;**345** doi: 10.1136/bmj.e6341.
6. Coulter A, Entwistle VA, Eccles A, Ryan S, Shepperd S, Perera R. Personalised care planning for adults with chronic or long-term health conditions. *Cochrane Database Syst Rev* 2015(3):Cd010523 doi: 10.1002/14651858.CD010523.pub2.
7. Muth C, van den Akker M, Blom JW, et al. The Ariadne principles: how to handle multimorbidity in primary care consultations. *BMC Med* 2014;**12**:223 doi: 10.1186/s12916-014-0223-1.
8. National Institute for Health and Care Excellence. Multimorbidity: clinical assessment and management NICE guideline [NG56]. London, 2016.
9. Reeves D, Hann M, Rick J, et al. Care plans and care planning in the management of long-term conditions in the UK: a controlled prospective cohort study. *Br J Gen Pract* 2014;**64**(626):e568-75 doi: 10.3399/bjgp14X681385.
10. Burt J, Roland M, Paddison C, et al. Prevalence and benefits of care plans and care planning for people with long-term conditions in England. *J Health Serv Res Policy* 2012;**17 Suppl 1**:64-71 doi: 10.1258/jhsrp.2011.010172.
11. Vermunt NPCA, Harmsen M, Westert GP, Olde Rikkert MGM, Faber MJ. Collaborative goal setting with elderly patients with chronic disease or multimorbidity: a systematic review. *BMC Geriatr* 2017;**17**:167 doi: 10.1186/s12877-017-0534-0.
12. NHS England. Enhanced service specification: Avoiding unplanned admissions: proactive case finding and patient review for vulnerable people, 2014.
13. Steel N, Clark A, Ford J, et al. Goal-setting in care planning for people with multimorbidity: feasibility study and intervention refinement Norwich: University of East Anglia,, 2016.
14. English indices of deprivation 2015. Ministry of Housing, Communities & Local Government, London, 2015.
15. Elwyn G, Frosch D, Thomson R, et al. Shared Decision Making: A Model for Clinical Practice. *J Gen Intern Med* 2012;**27**(10):1361-67 doi: 10.1007/s11606-012-2077-6.
16. Elwyn G, Durand MA, Song J, et al. A three-talk model for shared decision making: multistage consultation process. *BMJ* 2017;**359** doi: 10.1136/bmj.j4891.
17. Silverman J, Kurtz S, Draper J. *Skills for communicating with patients*: CRC Press, 2016.
18. Herdman M, Gudex C, Lloyd A, et al. Development and preliminary testing of the new five-level version of EQ-5D (EQ-5D-5L). *Qual Life Res* 2011;**20**(10):1727-36 doi: 10.1007/s11136-011-9903-x.
19. Coast J, Flynn TN, Natarajan L, et al. Valuing the ICECAP capability index for older people. *Soc Sci Med* 2008;**67**(5):874-82 doi: 10.1016/j.socscimed.2008.05.015.
20. Brodaty H, Pond D, Kemp NM, et al. The GPCOG: a new screening test for dementia designed for general practice. *J Am Geriatr Soc* 2002;**50**(3):530-4.
21. Glasgow RE, Wagner EH, Schaefer J, Mahoney LD, Reid RJ, Greene SM. Development and validation of the Patient Assessment of Chronic Illness Care (PACIC). *Med Care* 2005;**43**(5):436-44.

For peer review only - http://bmjopen.bmj.com/site/about/guidelines.xhtml

22. Employers N. 16 General Medical Services (GMS) contract Quality and Outcomes Framework (QOF). *NHS England Gateway reference* 2014;**1264**(2014):2014-15.
23. Barr PJ, Thompson R, Walsh T, Grande SW, Ozanne EM, Elwyn G. The psychometric properties of CollaboRATE: a fast and frugal patient-reported measure of the shared decision-making process. *J Med Internet Res* 2014;**16**(1):e2 doi: 10.2196/jmir.3085.
24. Melbourne E, Sinclair K, Durand MA, Legare F, Elwyn G. Developing a dyadic OPTION scale to measure perceptions of shared decision making. *Patient Educ Couns* 2010;**78**(2):177-83 doi: 10.1016/j.pec.2009.07.009.
25. Turner-Stokes L. Goal attainment scaling (GAS) in rehabilitation: a practical guide. *Clin Rehabil* 2009;**23**(4):362-70 doi: 10.1177/0269215508101742.
26. Barr PJ, O'Malley AJ, Tsulukidze M, Gionfriddo MR, Montori V, Elwyn G. The psychometric properties of Observer OPTION5, an observer measure of shared decision making. *Patient Educ Couns* 2015;**98**(8):970-76 doi: https://doi.org/10.1016/j.pec.2015.04.010.
27. Eldridge SM, Costelloe CE, Kahan BC, Lancaster GA, Kerry SM. How big should the pilot study for my cluster randomised trial be? *Stat Methods Med Res* 2016;**25**(3):1039-56 doi: 10.1177/0962280215588242.
28. Department of Health and Social Care. NHS reference costs 2015 to 2016. London, 2016.
29. Curtis L. Unit Costs of Health and Social Care 2017. In: Personal Social Services Research Unit, ed. Canterbury: University of Kent 2017.
30. Drummond MF, Sculpher MJ, Claxton K, Stoddart GL, Torrance GW. *Methods for the economic evaluation of health care programmes*: Oxford university press, 2015.
31. Drew P, Chatwin J, Collins S. Conversation analysis: a method for research into interactions between patients and health-care professionals. *Health Expect* 2001;**4**(1):58-70.
32. Ritchie J, Lewis J, Nicholls CM, Ormston R. *Qualitative research practice: A guide for social science students and researchers*: Sage, 2013.
33. Pereira Gray DJ, Sidaway-Lee K, White E, Thorne A, Evans PH. Continuity of care with doctors—a matter of life and death? A systematic review of continuity of care and mortality. *BMJ Open* 2018;**8**(6) doi: 10.1136/bmjopen-2017-021161.
34. Battersby M, Harvey P, Mills PD, et al. SA HealthPlus: a controlled trial of a statewide application of a generic model of chronic illness care. *Milbank Q* 2007;**85**(1):37-67 doi: 10.1111/j.1468-0009.2007.00476.x.
35. NHS England. Supporting routine frailty identification and frailty through the GP Contract 2017/2018. London, 2017.
36. 2011 Rural/urban Classification. In: Office for National Statistics, ed. London, 2011.

For peer review only - http://bmjopen.bmj.com/site/about/guidelines.xhtml

Figure 1: Consort flow diagram (N= number of practice, n= number of individuals)

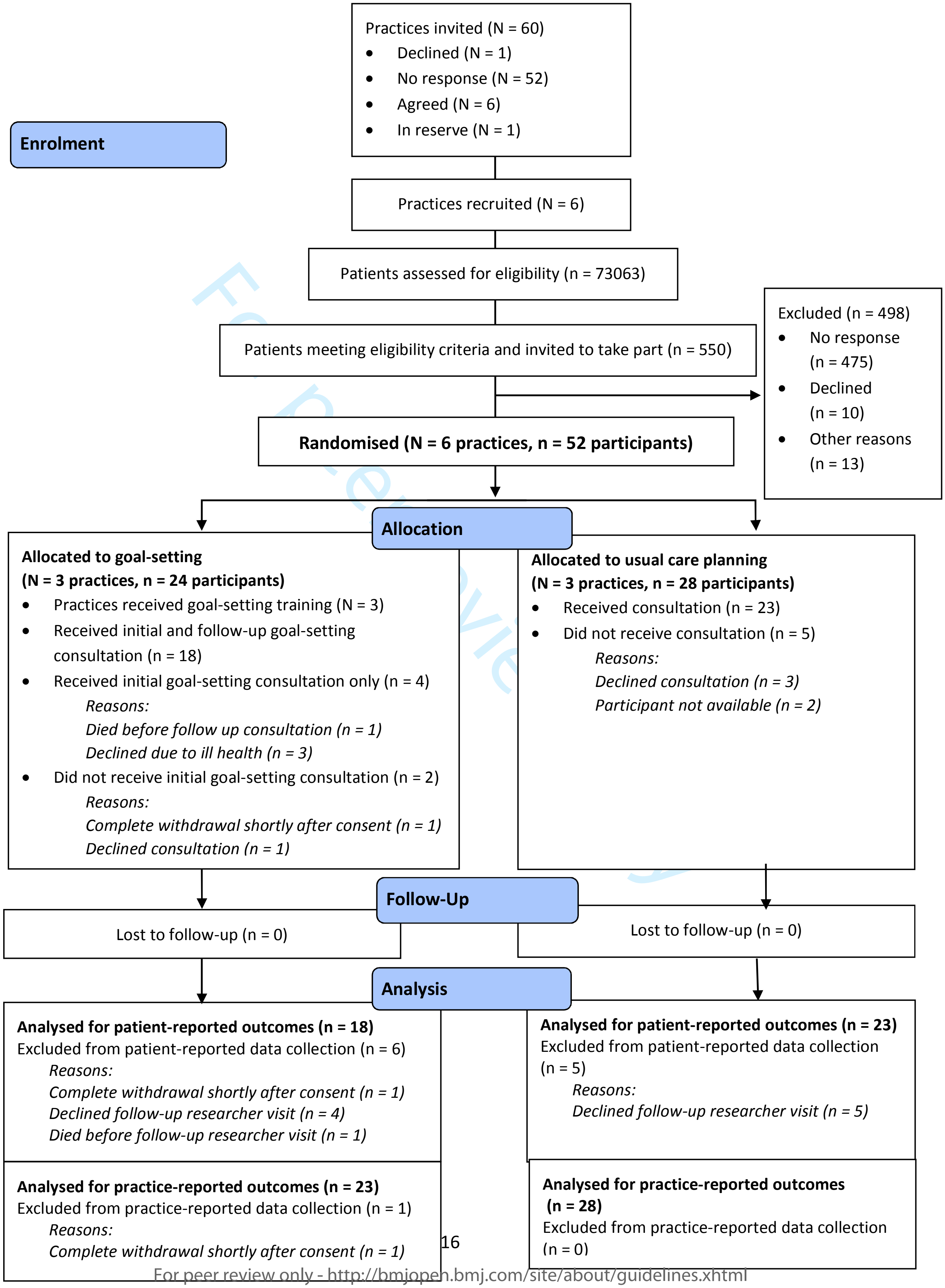

For peer review only - http://bmjopen.bmj.com/site/about/guidelines.xhtml

Table 1: Practice level recruitment and baseline characteristics

	Goal-setting				Usual care planning				Grand total
	Practice 1	Practice 2	Practice 3	Goal-setting total	Practice 4	Practice 5	Practice 6	Control total	
**Practice level recruitment**									
Patients assessed for eligibility (n)	9067	14845	6791	30703	18540	10381	13439	42360	73063
Patients invited (n)	77	108	47	232	108	124	86	318	550
Recruited (n)	11	9	4	24	8	10	10	28	52
**Practice level baseline characteristics**									
Age (mean SD)	78.5 (10.4)	82.9 (6.7)	80.3 (8.3)	80.4 (8.7)	85.8 (5.4)	69.5 (7.5)	78.0 (7.5)	77.1 (9.4)	78.7 (9.2)
Female (n %)	8 (73%)	4 (44%)	1 (25%)	13 (54%)	5 (62%)	3 (30%)	3 (30%)	11 (39%)	24 (46%)
Number of medications (median IQR)	13.5 (11.8 to 17.3)	10.0 (9.0 to 17.5)	15.5 (6.5 to 18.5)	13.0 (10.0 to 17.0)	10.5 (9.3 to 15.5)	12.0 (6.5 to 17.5)	12.0 (4.0 to 16.8)	11.5 (7.0 to 16.0)	13.0 (9.0 to 17.0)
Number of diagnoses* (median IQR)	5.0 (3.8 to 5.3)	4.0 (3.0 to 6.5)	7.5 (3.0 to 9.0)	5.0 (3.0 to 5.0)	5.0 (3.0 to 5.0)	4.5 (3.8 to 5.0)	3.0 (2.0 to 4.3)	4.0 (3.0 to 5.0)	4.0 (3.0 to 6.0)

n= number, SD = standard deviation, IQR = Interquartile Range, * = based on Barnett list [1]

For peer review only - http://bmjopen.bmj.com/site/about/guidelines.xhtml

For peer review only

Table 2: Characteristics of participating practices

	Goal-setting			Usual care planning		
	**Practice 1**	**Practice 2**	**Practice 3**	**Practice 4**	**Practice 5**	**Practice 6**
Practice rurality*	Village	Town and fringe	Town and fringe	Urban >10K	Urban >10K	Urban >10K
Patient population	5000 to 9,900	10,000 to 14,900	5000 to 9,900	>14,900	10,000 to 14,900	10,000 to 14,900
IMD practice decile	7	5	7	9	5	5
Characteristics of participating GPs	2 x male (partners, 2 x PT)	1 x male, 1 x female (partners, 2 x FT)	1 x male (partner, PT)	1 x male, 1 x female (partners, 1 x FT, 1 x PT)	2 x female (partners, 2 x PT)	2 x female (partners, 2 x PT)
Years qualified of participating GPs	GP014 = >20 yrs; GP018 = 10 to 20 yrs	GP025 = <10 yrs; GP026 = 10 to 20 yrs	GP038 = 10 to 20 yrs	GP046 = >20 yrs; GP047 = >20 yrs	GP053 = >20 yrs; GP055 = >20 yrs	GP061 = 10 to 20 yrs; GP067 = 10 to 20 yrs

*ONS indicator 2011 [36], IMD = Index of Multiple Deprivation (1= most deprived and 10 least deprived), partner = GP with responsibility for the practice, FT= full time, PT = part time,

For peer review only

For peer review only - http://bmjopen.bmj.com/site/about/guidelines.xhtml

Table 3: Baseline characteristics of patient participants

Variable		Usual care planning	Goal-setting
Number		28	24
Female n (%)		11 (39%)	13 (54%)
Age mean (SD)		77.18 (9.42)	80.42 (8.72)
Number of medications median (IQR)		11.50 (7.00, 16.00)	13.00 (10.00, 17.00)
GPCOG category n (%)	Impairment and further investigations implied	1 (4%)	0 (0%)
	Informant interview required	17 (61%)	19 (79%)
	No cognitive impairment	10 (36%)	5 (21%)
PACIC score mean (SD)		1.49 (0.35)	1.92 (0.71)
EQ-5Q-5L score mean (SD)		0.53 (0.35)	0.52 (0.30)
EQ-5D-5L Visual Analogue Scale mean (SD)		60.18 (23.39)	64.96 (18.16)
ICECAP-O score mean (SD)		0.74 (0.24)	0.77 (0.14)
Number of diagnoses* median (IQR)		4.00 (3.00, 5.00)	5.00 (3.00, 6.00)
IMD national quartile n (%)	1	5 (18%)	0 (0%)
	2	9 (32%)	14 (58%)
	3	3 (11%)	10 (42%)
	4	11 (39%)	0 (0%)
Marital status n (%)	Divorced	0 (0%)	2 (8%)
	Living with partner	0 (0%)	2 (8%)
	Married	12 (43%)	10 (42%)
	Single	2 (7%)	4 (17%)
	Widowed	14 (50%)	6 (25%)

N= number, SD = standard deviation, IQR = Interquartile Range, GPCOG = General Practitioner assessment of Cognition, PACIC = Patients Assessment Chronic Illness Care, EQ-5Q-5L = 5 level EQ-5D, ICECAP-O = ICEpop CAPability measure for Older people, * = based on Barnett list [1], IMD = Index of Multiple Deprivation

For peer review only

For peer review only - http://bmjopen.bmj.com/site/about/guidelines.xhtml

Table 4: Characteristics of baseline consultations

	Intervention group				Control group				Mean difference between intervention and control (95% CI)
	Practice 1 (n = 10)	Practice 2 (n = 8)	Practice 3 (n = 4)	Intervention total (n = 22)	Practice 4 (n = 7)	Practice 5 (n = 9)	Practice 6 (n = 7)	Control total (n = 23)	
Duration of initial consultation (mins) mean (SD)	24.1 (4.0)	23.3 (4.4)	19.9 (6.2)	23.0 (4.6)	14.3 (4.8)	25.2 (5.7)	16.3 (4.1)	19.2 (6.9)	3.88 (-3.25,11.01)
Dyadic OPTION scores mean (SD)	65.3 (9.0)	63.2 (6.4)	62.5 (3.6)	64.0 (7.2)	63.5 (13.0)	62.7 (4.0)	42.1 (20.4)	56.6 (16.2)	7.57 (-6.37,21.50)
CollaboRATE scores mean (SD)	7.8 (1.0)	8.5 (0.9)	8.8 (0.2)	8.2 (1.0)	7.0 (2.6)	8.6 (0.7)	8.7 (0.6)	8.1 (1.8)	0.20 (-1.06,1.47)
GP:patient word count ratio mean (SD)	1.23 (0.40)	1.41 (0.78)	1.50 (1.05)	1.35 (0.67)	1.13 (0.45)	1.92 (0.75)	1.39 (0.52)	1.52 (0.67)	-0.14 (-0.65,0.37)

SD= standard deviation, n= number, 95%CI = 95% confidence interval

For peer review only - http://bmjopen.bmj.com/site/about/guidelines.xhtml

Table 5: Number and attainment score of goals set

		Practice 1 (n = 10)	Practice 2 (n = 8)	Practice 3 (n = 4)	Total
Goals set		27	16	7	50
Number of goal per patient	1 goal set	0	2	1	3
	2 goals set	3	4	3	10
	3 goals set	7	2	0	9
Number of goals with data available for attainment scoring		21	15	6	42
Mean score of goal attainment per patient		1.43	1.67	1.0	1.45
Goal attainment n (%)	worse than expected	1 (4.8)	2 (13.3)	1 (16.7)	4 (9.5)
	no change	4 (19.0)	0 (0.0)	2 (33.3)	6 (14.3)
	partially attained	9 (42.9)	5 (33.3)	1 (16.7)	15 (35.7)
	as expected	2 (9.5)	3 (20.0)	1 (16.7)	6 (14.3)
	a little more	2 (9.5)	4 (26.7)	0 (0.0)	6 (14.3)
	a lot more than expected	3 (14.3)	1 (6.7)	1 (16.7)	5 (11.9)

n = number of participants

For peer review only

For peer review only - http://bmjopen.bmj.com/site/about/guidelines.xhtml

Table 6: Categories of goals set

Goal categories	Number of goals
Management of chronic condition (non-medication)	9
Walking-related	8
Maintain interests	5
Management of chronic condition (medication-related)	5
Gain weight	4
Social participation	3
Healthy living	3
Balance/mobility	3
Gardening-related	3
Manual dexterity	3
Mental health	2
End of life management	1
Cooking/food preparation	1
Grand Total	50

For peer review only

Table 7: Change in outcome measures between groups

Variable	Change from baseline to follow-up mean (SD)				Mean difference between goal-setting and usual care planning (95% CI)	p-value	Intraclass correlation coefficient (95% CI)
	n	Usual care planning	n	Goal-setting			
Number of medication	28	0.29 (2.65)	23	1.04 (3.21)	0.76 (-0.85,2.37)	0.356	0.00
GPCOG	23	-0.57 (2.02)	19	-0.58 (2.63)	0.09 (-1.65,1.84)	0.918	0.08 (0.00,0.77)
PACIC	23	0.40 (0.69)	18	0.31 (0.98)	-0.09 (-0.60,0.42)	0.730	0.00
EQ-5D-5L	23	-0.02 (0.19)	18	-0.01 (0.15)	0.02 (-0.11,0.13)	0.847	0.05 (0.00,0.94)
ICECAP-O	22	0.06 (0.14)	17	-0.02 (0.06)	-0.08 (-0.15,-0.00)	0.037	0.00

SD = standard deviation, GPCOG = General Practitioner assessment of Cognition, PACIC = Patients Assessment Chronic Illness Care, EQ-5Q-5L = 5 level EQ-5D, ICECAP-O = ICEpop CAPability measure for Older people, 95%CI = 95% confidence interval

For peer review only - http://bmjopen.bmj.com/site/about/guidelines.xhtml

Table 8: Costs associated with health care use in GoalPlan study

Resource use	6-months prior to recruitment						Recruitment to 6-month follow-up					
	Usual care planning			Goal-setting			Usual care planning			Goal-setting		
	Total contacts n	Total cost £	Mean cost £ (SD)	Total contacts n	Total cost £	Mean cost £ (SD)	Total contacts n	Total cost £	Mean cost £ (SD)	Total contacts n	Total cost £	Mean cost £ (SD)
Community based services												
GP	157	4,636	166 (164)	89	2,464	107 (115)	177	5,150	184 (150)	124	4,002	174 (145)
Other practice based	97	922	33 (42)	108	1,080	47 (30)	152	1,823	65 (58)	149	1,529	66 (53)
District Nurse	148	3,582	128 (546)	198	6,450	280 (1297)	100	2,879	103 (321)	241	7,450	324 (1384)
Other	72	1,434	51 (132)	72	2,601	113 (193)	189	7,652	273 (355)	97	5,510	240 (224)
All community based	474	10,575	378 (778)	467	12,594	548 (1520)	618	15,681	560 (719)	611	16,962	737 (1537)
Inpatient	4	11,291	403 (1113)	16	28,054	1220 (2584)	12	35,055	1252 (2203)	13	39,889	1734 (4815)
Outpatient	45	4,848	173 (208)	51	7,381	321 (397)	41	4,424	158 (202)	52	6,295	274 (329)
A&E	1	138	5 (26)	6	826	36 (74)	15	2,066	74 (109)	16	2,204	96 (128)
Total for all costs		26,853	959 (1776)		48,856	2124 (4031)		57,226	2044 (2665)		65,349	2841 (4968)

SD = standard deviation, A&E = Accident and Emergency

For peer review only - http://bmjopen.bmj.com/site/about/guidelines.xhtml

Supplementary Table 1: Characteristics of those who participated compared with those who did not

	Participation	Non-participation
Number	52	498
Age mean (SD)	78.5 (9.0)	79.6 (12.2)
Female %	46.2%	53.8%
IMD decile mean (SD)	5.8 (2.3)	5.3 (2.2)

SD = standard deviation, IMD = Index of Multiple Deprivation

For peer review only

For peer review only - http://bmjopen.bmj.com/site/about/guidelines.xhtml

Supplementary Table 2: Quality and Outcomes Framework data

		Goal-setting	Usual care planning
BMI	No of participants	2	3
	Baseline mean (SD)	28.4 (1.9)	37.8 (8.0)
	Follow-up (mean, SD)	28.5 (3.6)	37.0 (9.5)
	Diff (mean, SD)	0.1 (1.7)	-0.8 (2.7)
BP, mmHg	No of participants	5	5
	Baseline systolic (mean, SD)	133.5 (6.5)	127.5 (19.0)
	Baseline diastolic (mean, SD)	70.7 (4.4)	69.2 (5.5)
	Follow-up systolic (mean, SD)	144.7 (7.0)	124.4 (6.2)
	Follow-up diastolic (mean, SD)	80.6 (4.7)	67.1 (5.6)
	Mean diff systolic (mean, SD)	11.2 (12.6)	-3.1 (14.4)
	Mean diff diastolic (mean, SD)	9.9 (3.1)	-2.1 (8.7)
	Baseline Qof target met (150/90)	5/5	5/5
	Follow-up Qof target met (150/90)	4/5	5/5
eGRFR, mL/min/1,73m^2	No of participants	4	6
	Baseline (mean, SD)	54 (14)	57 (24)
	Follow-up (mean, SD)	56 (17)	59 (25)
	Mean diff (mean, SD)	2 (5)	2 (3)
HB1Ac, mmol/mol	No of participants	1	3
	Baseline (mean, SD)	80 (NA)	39 (3)
	Follow-up (mean, SD)	87 (NA)	43 (6)
	Mean diff (mean, SD)	7 (NA)	4 (3)
	Baseline Qof target met - Diabetes and HB1Ac <59	0/1	1/1
	Baseline Qof target met - Diabetes and HB1Ac <64	0/1	1/1
	Baseline Qof target met - Diabetes and HB1Ac <75	0/1	1/1
	Follow-up Qof target met - Diabetes and HB1Ac <59	0/1	1/1
	Follow-up Qof target met - Diabetes and HB1Ac <64	0/1	1/1
	Follow-up Qof target met - Diabetes and HB1Ac <75	0/1	1/1
Total cholesterol, mg/dL	No of participants	2	1
	Baseline (mean, SD)	2.8 (0.7)	4.2 (NA)
	Follow-up (mean, SD)	3.9 (1.1)	4.9 (NA)
	Mean diff (mean, SD)	1.1 (0.5)	0.7 (NA)
HDL cholesterol, mg/dL	No of participants	2	0
	Baseline (mean, SD)	0.84 (0.19)	NA
	Follow-up (mean, SD)	1.01 (0.4)	NA
	Mean diff (mean, SD)	0.17 (0.15)	NA

BMI = body mass index, SD= standard deviation, BP = blood pressure, eGFR = estimated glomerular filtration rate, HB1Ac = glycated haemoglobin, Qof = Quality and Outcomes Framework, HDL = High Density Lipoproteins, NA= not applicable

For peer review only - http://bmjopen.bmj.com/site/about/guidelines.xhtml

University of East Anglia

GoalPlan Study:
Goal-setting form
for completion before care plan appointment

Part 1) What are your goals? What is important to you?

Write down what you would really like to do or achieve over the next 6 months, even if you think it may not be related to your health. Think about things that you would like to do in your personal, home, work, and social life—things that you ***need*** to do, ***want*** to do and / or ***enjoy doing***. Then list them in order of priority— starting with 1 for the goal that matters to you most and that you would like to focus on at the moment. To help you, we have put a completed example overleaf.

**What do you really want to achieve over the next 6 months? (your goals)**

For peer review only

Example

- *I want to be able to do more in the garden as it relaxes me*
- *I want to lose weight so I can walk more confidently at my daughter's wedding and fit into my favourite outfit again*
- *I want to be able to get more exercise as it used to help me sleep well*
- *I want to get back to driving so I can visit my friend at his house every week*
- *I want to be able to go out with friends once a week again for lunch*

Part 2) Why are these goals important to you?

We now ask you to focus on your goals from Part 1, and to think about why they are important to you. Below is an example of how to find out if a goal you have come up with is important for its own sake, or if it is important because it will help you achieve something else. We ask you to do this exercise with up to three of your goals overleaf.

Part 3) What are the first steps you would like to take towards achieving this goal or goals?

Having identified your most important goals, the final step on this form is to start thing about steps you would like to take to achieve those goals. This is also included in the example below. Please do the same for your goals overleaf.

EXAMPLE GOAL 1: *I want to be able to do more in the garden*

→ **Why are these goals important to you?** →

Gardening is my hobby and gives me pleasure. It is my main form of exercise.

→ **What first steps can I take towards achieving this goal?** →

Discuss changes to medicines with GP – might reducing medicines help me be more active?

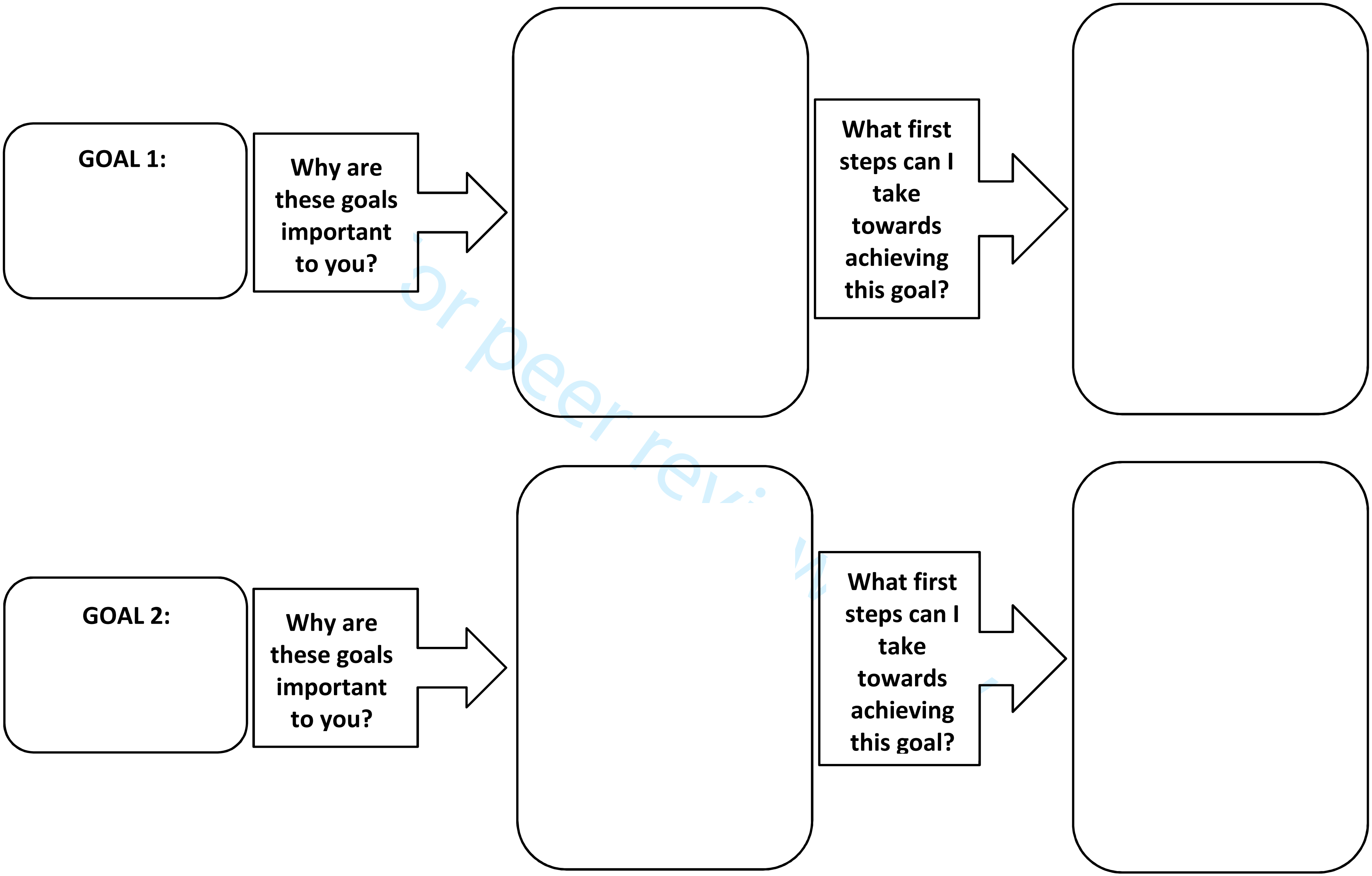

For peer review only - http://bmjopen.bmj.com/site/about/guidelines.xhtml

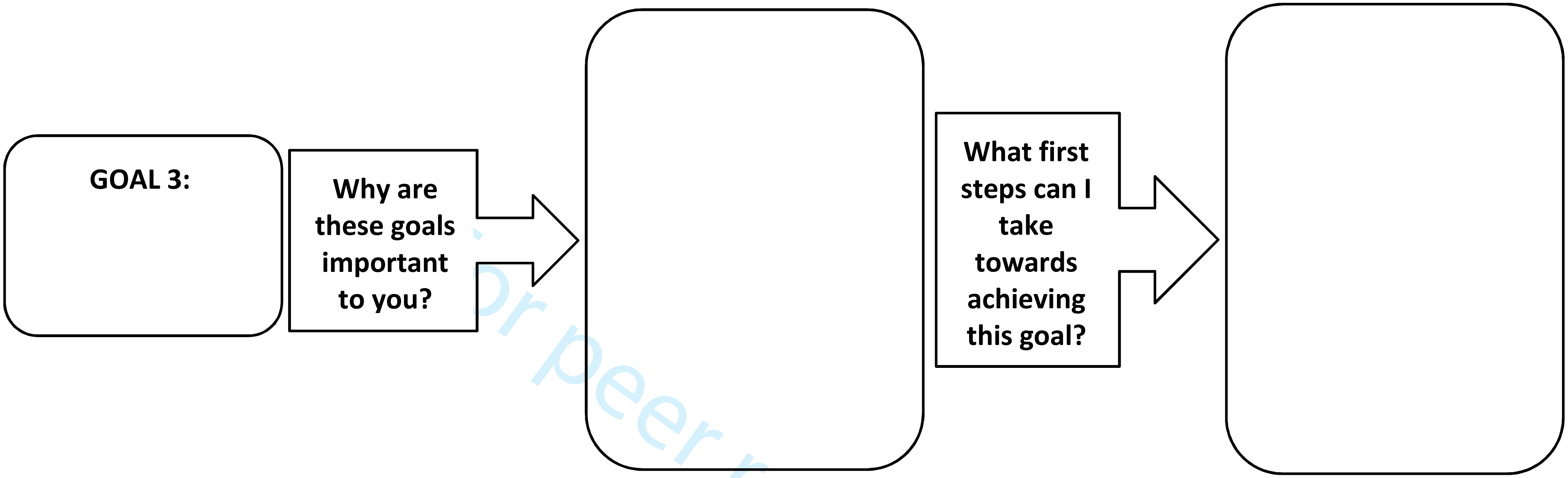

Thank you for completing this goal-setting form. Please take it with you to your care plan appointment. Your GP will discuss it with you and together you will make specific plans to achieve your goals.

For peer review only - http://bmjopen.bmj.com/site/about/guidelines.xhtml

GoalPlan Study

GAS-Light verbal scoring system

**Goal 1:**			Tick
**At Baseline**	With respect to this goal do they have?	Some attainment	☐
		No attainment (as bad as they could be)	☐
**At 6 month review:** Was the goal attained?	**Yes**	A lot more	☐
		A little more	☐
		As expected	☐
	**No**	Partially attained	☐
		No change	☐
		Got worse	☐

PTO for further goals

For peer review only

For peer review only - http://bmjopen.bmj.com/site/about/guidelines.xhtml

**Goal 2:**			Tick
**At Baseline**	With respect to this goal do they have?	Some attainment	☐
		No attainment<br>(as bad as they could be)	☐
**At 6 month review:**<br><br>Was the goal attained?	**Yes**	A lot more	☐
		A little more	☐
		As expected	☐
	**No**	Partially attained	☐
		No change	☐
		Got worse	☐

For peer review only

PTO for further goals

For peer review only - http://bmjopen.bmj.com/site/about/guidelines.xhtml

**Goal 3:**			Tick
**At Baseline**	With respect to this goal do they have?	Some attainment	☐
		No attainment (as bad as they could be)	☐
**At 6 month review:** Was the goal attained?	**Yes**	A lot more	☐
		A little more	☐
		As expected	☐
	**No**	Partially attained	☐
		No change	☐
		Got worse	☐

For peer review only

For peer review only - http://bmjopen.bmj.com/site/about/guidelines.xhtml

CONSORT 2010 checklist of information to include when reporting a pilot or feasibility trial*

Section/Topic	Item No	Checklist item	Reported on page No
**Title and abstract**			
	1a	Identification as a pilot or feasibility randomised trial in the title	1
	1b	Structured summary of pilot trial design, methods, results, and conclusions (for specific guidance see CONSORT abstract extension for pilot trials)	2
**Introduction**			
Background and objectives	2a	Scientific background and explanation of rationale for future definitive trial, and reasons for randomised pilot trial	4
	2b	Specific objectives or research questions for pilot trial	4
**Methods**			
Trial design	3a	Description of pilot trial design (such as parallel, factorial) including allocation ratio	5
	3b	Important changes to methods after pilot trial commencement (such as eligibility criteria), with reasons	5
Participants	4a	Eligibility criteria for participants	5
	4b	Settings and locations where the data were collected	5
	4c	How participants were identified and consented	5
Interventions	5	The interventions for each group with sufficient details to allow replication, including how and when they were actually administered	5-6
Outcomes	6a	Completely defined prespecified assessments or measurements to address each pilot trial objective specified in 2b, including how and when they were assessed	6
	6b	Any changes to pilot trial assessments or measurements after the pilot trial commenced, with reasons	5
	6c	If applicable, prespecified criteria used to judge whether, or how, to proceed with future definitive trial	NA
Sample size	7a	Rationale for numbers in the pilot trial	5
	7b	When applicable, explanation of any interim analyses and stopping guidelines	NA
Randomisation:			
Sequence generation	8a	Method used to generate the random allocation sequence	5
	8b	Type of randomisation(s); details of any restriction (such as blocking and block size)	5
Allocation concealment mechanism	9	Mechanism used to implement the random allocation sequence (such as sequentially numbered containers), describing any steps taken to conceal the sequence until interventions were assigned	5

For peer review only

Implementation	10	Who generated the random allocation sequence, who enrolled participants, and who assigned participants to interventions	5
Blinding	11a	If done, who was blinded after assignment to interventions (for example, participants, care providers, those assessing outcomes) and how	NA
	11b	If relevant, description of the similarity of interventions	NA
Statistical methods	12	Methods used to address each pilot trial objective whether qualitative or quantitative	6
**Results**			
Participant flow (a diagram is strongly recommended)	13a	For each group, the numbers of participants who were approached and/or assessed for eligibility, randomly assigned, received intended treatment, and were assessed for each objective	Figure 1
	13b	For each group, losses and exclusions after randomisation, together with reasons	Figure 1
Recruitment	14a	Dates defining the periods of recruitment and follow-up	6
	14b	Why the pilot trial ended or was stopped	NA
Baseline data	15	A table showing baseline demographic and clinical characteristics for each group	Table 3
Numbers analysed	16	For each objective, number of participants (denominator) included in each analysis. If relevant, these numbers should be by randomised group	Tables 4, 5 and 7
Outcomes and estimation	17	For each objective, results including expressions of uncertainty (such as 95% confidence interval) for any estimates. If relevant, these results should be by randomised group	Table 7
Ancillary analyses	18	Results of any other analyses performed that could be used to inform the future definitive trial	NA
Harms	19	All important harms or unintended effects in each group (for specific guidance see CONSORT for harms)	9
	19a	If relevant, other important unintended consequences	NA
**Discussion**			
Limitations	20	Pilot trial limitations, addressing sources of potential bias and remaining uncertainty about feasibility	11-12
Generalisability	21	Generalisability (applicability) of pilot trial methods and findings to future definitive trial and other studies	11-12
Interpretation	22	Interpretation consistent with pilot trial objectives and findings, balancing potential benefits and harms, and considering other relevant evidence	11-12
	22a	Implications for progression from pilot to future definitive trial, including any proposed amendments	11-12
**Other information**			
Registration	23	Registration number for pilot trial and name of trial registry	1
Protocol	24	Where the pilot trial protocol can be accessed, if available	1
Funding	25	Sources of funding and other support (such as supply of drugs), role of funders	13
	26	Ethical approval or approval by research review committee, confirmed with reference number	5

For peer review only

Citation: Eldridge SM, Chan CL, Campbell MJ, Bond CM, Hopewell S, Thabane L, et al. CONSORT 2010 statement: extension to randomised pilot and feasibility trials. BMJ. 2016;355.

*We strongly recommend reading this statement in conjunction with the CONSORT 2010, extension to randomised pilot and feasibility trials, Explanation and Elaboration for important clarifications on all the items. If relevant, we also recommend reading CONSORT extensions for cluster randomised trials, non-inferiority and equivalence trials, non-pharmacological treatments, herbal interventions, and pragmatic trials. Additional extensions are forthcoming: for those and for up to date references relevant to this checklist, see www.consort-statement.org.

For peer review only

CONSORT 2010 checklist of information to include when reporting a pilot or feasibility randomized trial in a journal or conference abstract

Item	Description	Reported on line number
Title	Identification of study as randomised pilot or feasibility trial	1
Authors *	Contact details for the corresponding author	1
Trial design	Description of pilot trial design (eg, parallel, cluster)	2
Methods		
Participants	Eligibility criteria for participants and the settings where the pilot trial was conducted	2
Interventions	Interventions intended for each group	2
Objective	Specific objectives of the pilot trial	2
Outcome	Prespecified assessment or measurement to address the pilot trial objectives**	2
Randomization	How participants were allocated to interventions	2
Blinding (masking)	Whether or not participants, care givers, and those assessing the outcomes were blinded to group assignment	2
Results		
Numbers randomized	Number of participants screened and randomised to each group for the pilot trial objectives**	2
Recruitment	Trial status†	
Numbers analysed	Number of participants analysed in each group for the pilot objectives**	2
Outcome	Results for the pilot objectives, including any expressions of uncertainty**	2
Harms	Important adverse events or side effects	2
Conclusions	General interpretation of the results of pilot trial and their implications for the future definitive trial	2
Trial registration	Registration number for pilot trial and name of trial register	1
Funding	Source of funding for pilot trial	13

Citation: Eldridge SM, Chan CL, Campbell MJ, Bond CM, Hopewell S, Thabane L, et al. CONSORT 2010 statement: extension to randomised pilot and feasibility trials. BMJ. 2016;355.

**this item is specific to conference abstracts*

***Space permitting, list all pilot trial objectives and give the results for each. Otherwise, report those that are a priori agreed as the most important to the decision to proceed with the future definitive RCT.*

†For conference abstracts.

For peer review only

BMJ Open

Can goal-setting for patients with multimorbidity improve outcomes in primary care?: cluster randomised feasibility trial

Journal:	*BMJ Open*
Manuscript ID	bmjopen-2018-025332.R1
Article Type:	Research
Date Submitted by the Author:	20-Nov-2018
Complete List of Authors:	Ford, John; University of East Anglia, Public Health<br>Lenaghan, E; University of East Anglia<br>Salter, Charlotte; University of East Anglia<br>Turner, David; University of East Anglia, Public Health and Primary Care<br>Shiner, A.; University of East Anglia<br>Clark, Allan; Norwich Medical School<br>Murdoch, Jamie; University of East Anglia, School of Nursing Sciences<br>Green, Carole<br>Lipp, Alistair; NHS England Midlands & East,, West Wing<br>Moseley, Annie<br>Wade, Tom; University of East Anglia<br>Winterburn, Sandra; University of East Anglia<br>Koopmans, Imogen; University of East Anglia, Public Health<br>James, Sarah; University of East Anglia, Public Health<br>Steel, Nicholas; University of East Anglia
**Primary Subject Heading**:	Public health
Secondary Subject Heading:	General practice / Family practice
Keywords:	PRIMARY CARE, HEALTH SERVICES ADMINISTRATION & MANAGEMENT, SOCIAL MEDICINE

SCHOLARONE™
Manuscripts

Title

Can goal-setting for patients with multimorbidity improve outcomes in primary care?: cluster randomised feasibility trial

Authors

John A. Ford, Elizabeth Lenaghan, Charlotte Salter, David A. Turner, Alice Shiner, Allan Clark, Jamie Murdoch, Carole Green, Alistair Lipp, Annie Moseley, Tom Wade, Sandra Winterburn, Imogen Koopmans, Sarah James, Nicholas Steel

Corresponding author

Prof Nicholas Steel

Norwich Medical School
University of East Anglia
Chancellor's Drive
Norwich
NR4 7TJ
n.steel@uea.ac.uk
01603 591161

For peer review only

Trials registration:
Title: Goal-setting in care planning for people with multimorbidity
Trial ID: ISRCTN13248305
Date registered: 21/12/2016
Link: http://www.isrctn.com/ISRCTN13248305

Protocol: Available at:
http://www.uea.ac.uk/documents/246046/14839702/GoalPlan+Research+Protocol+v1.2+170316.pdf/86549c5b-c4ed-435b-8719-4c3160f9793f

Word count: 5219 words

Keywords: goals, multimorbidity, primary health care, patient-centred care, randomized controlled trial

For peer review only - http://bmjopen.bmj.com/site/about/guidelines.xhtml

Abstract

Introduction

Goal-setting is recommended for patients with multimorbidity, but there is little evidence to support its use in general practice.

Objective

To assess the feasibility of goal-setting for patients with multimorbidity, before undertaking a definitive trial.

Design and setting

Cluster-randomised controlled feasibility trial of goal-setting compared to control in six general practices.

Participants

Adults with 2 or more long term health conditions and at risk of unplanned hospital admission.

Interventions

General Practitioners (GPs) underwent training and patients were asked to consider goals before an initial goal-setting consultation and a follow-up consultation six months later. The control group received usual care planning.

Outcome measures

Health-related quality of life (EQ5D5L), capability (ICEpop CAPability measure for Older people (ICECAP-O)), patient assessment of chronic illness care (PACIC) and health care use. All consultations were video or audio-recorded, and focus groups were held with participating GPs and patients.

Results

Fifty-two participants were recruited with a response rate of 12%. Full follow-up data were available for 41. In the goal-setting group, mean age was 80.4 years, 54% were female and the median number of prescribed medications was 13, compared to 77.2 years, 39% female and 11.5 medications in the control group. The mean initial consultation time was 23.0 minutes in the goal-setting group and 19.2 in the control group. Overall 28% of patient participants had no cognitive impairment. Participants set between one and three goals on a wide range of subjects, such as chronic disease management, walking, maintaining social and leisure interests, and weight management. Patient participants found goal-setting acceptable and would have liked more frequent follow-up. GPs unanimously liked goal-setting, felt it delivered more patient-centred care and highlighted the importance of training.

Conclusions

This goal-setting intervention was feasible to deliver in general practice. A larger, definitive study is needed to test its effectiveness.

For peer review only - http://bmjopen.bmj.com/site/about/guidelines.xhtml

Strengths and limitations of this study

- General practitioners and patients with multimorbidities both benefit from preparation before setting goals
- Recruitment reached target levels in five of six practices, but the patient response rate of 12% means that a definitive study will need sufficient numbers of patients with multimorbidity
- Existing measures of patient centred care are usually designed for a single specific treatment decision and were difficult to apply to goal setting consultations, where several goals were discussed
- The most relevant outcome measure for goal setting was the patient assessment of chronic illness care (PACIC), which includes a sub-scale for goal setting
- Qualitative data from video-recorded consultations and focus groups were vital to understand how goal-setting was implemented in practice, and how acceptable it was to GPs and patients.

For peer review only

For peer review only - http://bmjopen.bmj.com/site/about/guidelines.xhtml

Introduction

The rising number of long-term conditions and prescribed medications has increased the burden of treatment for patients [1 2]. People with multimorbidity (defined as two or more chronic conditions [2]) tend to have a lower quality of life and worse health than those with single conditions [3]. Medical outcomes that work well for relatively healthy patients (e.g. blood pressure control, or disease-free survival) may be inappropriate for patients with multimorbidity or severe disability [4 5], and the use of current single-disease guidelines in this group can encourage harmful polypharmacy with resulting drug-drug and drug-disease interactions [6].

The National Institute for Health and Care Excellence (NICE) recommends an approach to care that takes account of multimorbidity by establishing patient goals, values and priorities [7]. Goal setting is the sharing of realistic goals by health professionals and patients and agreement of the best course of action [8]. Goal setting enables patients and doctors to focus health care on the outcomes that are most important to the patient. Examples of outcomes that matter to patients may include maintaining independence, undertaking paid or voluntary work, preventing adverse outcomes (e.g. falls) and reducing treatment burden [7]. Despite the recommendation, there is little evidence to support the use of goal-setting between general practitioners and patients, and it is rarely used in primary care [8-10]. Goal setting should include shared decision making, the process by which health professionals and patients make decisions together based on the best available evidence [11], because both involve partnership working, choices, options and decisions. The difference is that shared decision making is usually concerned with specific clinical treatment decisions, whereas goal setting usually involves a wider discussion around ways to deliver outcomes that matter to the patient.

Goal-setting should be, but is not always, an important element of care planning. Care planning may be defined as ‘a conversation in which patients and clinicians agree on goals and actions for managing the patient's conditions’ [8]. For patients with long term health conditions, personalised care planning has been found to improve physical and psychological health, in addition improving capability to self-manage, compared to usual care [8]. A recent systematic review highlighted the need for evidence exploring ’the effects of personalised care planning on goal-attainment, especially patient’s personal goals as opposed to goals determined by clinicians or researchers‘ [12].

Our goal-setting intervention was designed within the context of a national recommendation that the top 2% of patients at risk of unplanned hospital admission should have a care plan [13]. We wanted to find out if a consultation focussed on goal-setting would improve outcomes for this patient group, compared to control (usual care planning) consultations. Before we could conduct a full trial to answer this question, we needed to answer questions about the feasibility of such a trial. We aimed to assess the feasibility of goal-setting for patients with multimorbidity, at high risk of hospital admission and eligible for a care planning consultation, with a view to undertaking a future definitive randomised controlled trial. Our objectives were to assess participant recruitment and retention, the acceptability of a goal-setting intervention to patients and GPs, the training needs of GPs, the content of control consultations, goal-setting and the feasibility of collecting relevant outcome measures.

For peer review only - http://bmjopen.bmj.com/site/about/guidelines.xhtml

Methods

We undertook a cluster randomised controlled feasibility trial of goal-setting compared to usual care in six general practices in the United Kingdom, with six months follow-up. Six months was long enough for patients and GPs to work towards the agreed goals, but not so long that the goals would have been forgotten. There were no significant changes to the protocol [14]. Research ethics approval was obtained from the NHS Research Ethics Committee (16/EM/0411). Participants were recruited between April and May 2017 and follow-up completed in February 2018.

General practices were invited via two emails through the East of England Clinical Research Network and recruited on a first-come first-served basis. To be eligible, practices had to be using risk stratification to identify patients at high risk of unplanned admission (for example by participating in the Avoiding Unplanned Admissions Enhanced Service [13]), have at least one Good Clinical Practice trained GP and nurse, be available to attend the goal-setting training and not be a single handed practice. Practices were reimbursed for staff time and travel to undertake the research and deliver the intervention. Patients were eligible if they were aged 18 or over, identified as in the top 2% for risk of unplanned admission and diagnosed with at least two of 40 morbidities in Barnett's analysis of multimorbidity [2].Patients were excluded if they were deemed to be unable to participate in goal-setting in the GP's professional opinion (e.g. advanced dementia or acute psychosis), had received a care planning consultation in the previous three months, or required translation services to communicate verbally.

Practice administrators searched their electronic patient register according to the eligibility criteria, and a GP then checked the resulting patient list for exclusion criteria. Eligible patients were sent a letter of invitation and participant information leaflet, with the intention of recruiting 10 patients per practice. The number of eligible patients ranged from 47 to 124 and all were invited. The protocol allowed GPs to opportunistically invite patients they thought might be interested, however no patients were recruited through this process. A study researcher visited interested patients at home to discuss the study and obtain written informed consent.

The Norwich Clinical Trials Unit independently randomised three practices to goal-setting and three to control, by simple block randomisation using a 1:1 ratio and sealed opaque envelopes. Practices were randomised after at least 10 expressions of interest were received from patients. It was not possible to blind participants, health professionals or researchers due to the nature of the intervention, with the exception of the statistician undertaking the analysis, who was blinded to the allocation.

Intervention

All five participating GPs from practices allocated to goal-setting (see Table 1) received training in a three hour experiential workshop, led by senior consultation skills tutors (CS and SW) and a GP with experience in communication skills training (AS). One other GP attended the training but withdrew prior to delivering the intervention for personal reasons. The training model we developed for goal setting adapted relevant elements of the work of Elwyn and colleagues on shared decision making [15 16] and of patient-centred care in the leading training model in clinical communication (the Calgary Cambridge Guide [17]). Our model adopted a structured, patient-centred stepped approach. Steps included preparation, goal elicitation, assessing options, making goals smart, decision-making and evaluation. Following an introduction to the study, the training was mainly experiential to enable GPs to rehearse existing skills and integrate additional skills for facilitating the goal-setting

For peer review only - http://bmjopen.bmj.com/site/about/guidelines.xhtml

process. Experiential methods included role-play, video analysis and interactive skill spotting . GPs were trained in groups of three and were given a detailed handbook in advance. The handbook contained information about the study and a "how to" guide for goal-setting, including theoretical background and examples of goal setting. The control group GPs received no training for this study and were asked to undertake a care planning consultation as they would usually do in routine clinical practice.

A study researcher discussed goal-setting and the associated paperwork with participants during the face-to-face baseline visit, which lasted approximately 15 minutes. The researcher gave all patient participants a patient-held goal-setting sheet (PGS), with three questions to consider prior to their consultation. The questions (Supplementary Appendix 1) were:

- What are your goals? What is important to you? What do you really want to achieve over the next six months?
- Why are these goals important to you?
- What are the first steps you would like to take towards achieving this goal or goals?

The goal-setting consultations were held with the participating GPs even if they were different from the patient's usual GP. During the initial goal-setting consultation GPs, in partnership with participants, documented the goals which had been agreed. GPs then provided support, within their clinical expertise and with the help of other health care professionals, to help patients achieve their goals, for example by providing information on local groups and services. Participants in both the goal-setting and control groups had an initial consultation which lasted about 20 minutes, but only patients in the goal-setting arm were invited back for a follow-up consultation after six months to discuss their goal attainment.

Data and statistical analysis

We collected quantitative and qualitative data to meet the feasibility study objectives. Data were collected from patients during a researcher visit at baseline and six months were: health-related quality of life (EQ-5D-5L [18]); capability (as measured through the five attributes of attachment, security, role, enjoyment and control in the ICEpop CAPability measure for Older people questionnaire (ICECAP-O) [19])(ICEPOP is the name of the UK MRC-funded programme through which the index was developed), cognition (general practitioner assessment of cognition scale (GP-COG) [20]) and patient centred care (patient assessment of care for chronic conditions scale (PACIC) [21]). Data collected from the electronic patient record included age, sex and postcode Index of Multiple Deprivation (IMD) score (baseline only), medications on repeat prescription, diagnoses, achievement of relevant quality of care indicators in the Quality and Outcomes Framework [22] and primary and secondary care use (see health economic section below for more details). Practice data were collected before randomisation and patient data were collected after.

GPs and patient participants were asked to complete an assessment of shared decision making during each consultation using the CollaboRATE scale [23] for patients and dyadic OPTION scale [24] for GPs. GPs and patients in the goal-setting group were asked to discuss and complete a goal attainment scaling (GAS-Light) questionnaire [25] (See Supplementary Appendix 2) at the second consultation. Goal attainment was scored using the following system: -1 = worse than expected, 0 = no change, 1 = partially attained, 2 = as expected, 3 = a little more and 4 = a lot more than expected.

All initial consultations were video (n=41) or audio recorded (n=4) and transcribed. Three team members scored the consultations using the observer OPTION measure to assess shared decision making [26]. One focus group was held with patients and one with GPs from the goal-setting group

For peer review only - http://bmjopen.bmj.com/site/about/guidelines.xhtml

at the end of the six month follow-up period to discuss perspectives, experiences and overall acceptability of the goal-setting intervention. All patients in the intervention group were sent a letter of invitation to the focus group, except two who indicated at the researcher visit they did not want to take part. Both focus groups lasted about 90 minutes, were held at the university, guided by a topic guide, audio-recorded and transcribed. Patient or GP participants unable to attend the focus groups were interviewed by phone or face-to-face using the same topic guide.

We calculated the recruitment rate by practice and by randomisation group. Demographic variables were compared for those recruited and those not recruited. The characteristics of baseline consultations were summarised both by practice and by intervention group. Key characteristics were compared using a linear mixed model with practice included as a random effect.

The change in outcome measures from baseline to follow-up was summarised using descriptive statistics by randomisation group. We estimated the difference between randomisation groups using a linear mixed model with practice included as a random effect. This would allow the estimation of potential differences in a full-scale trial. The intra-cluster correlation coefficient was estimated for each outcome, however great care should be taken in the interpretation of these due to the small number of clusters [27].

Health economic evaluation

Data were collected on resource use from an NHS perspective to test data collection processes and to inform a future health economic evaluation estimating quality adjusted life years (QALYs). A record was kept of resources required to provide GP training, as well as the length of initial and follow-up goal-setting consultations. Additional health care resource use was extracted from electronic health records by practices supported by a study researcher (EL) for the six-months prior to randomisation and from randomisation to follow-up. Health care use was collected for: day-case and inpatient hospital admissions; outpatient visits; accident and emergency visits (A&E); consultations at the GP practice (GP, practice nurse, health care assistant, nurse practitioners); and other contacts, such as district nursing, allied health professional contacts, ambulance call outs, and specialist nursing contacts.

Resource use was costed using the NHS reference costs [28] for secondary care and a published source for primary care contacts [29]. NHS reference costs were used to estimate a weighted average cost for day cases, non-elective short stay, non-elective long stay, and elective admissions. For longer stays, additional days were costed using a weighted average of all excess bed day costs. For the first and second GP consultations in the goal-setting group, we had data on length of consultation and setting. The cost of providing training was estimated from a description given by the study researcher of duration and required staff. The cost of academic staff time was estimated using University pay scales (including employer's national insurance and superannuation payments). As the training would have relevance beyond the duration of the study, we estimated a useful life of 3 years and calculated an annual equivalent cost [30]. All costs are in 2015/16 UK pounds sterling. As the duration of the study was six-months, we did not discount costs and benefits. As the study size was very small with great variability in estimates of cost and effect, we did not estimate formal cost-effectiveness.

Qualitative analysis

The video and audio recordings of control and goal-setting consultations were compared by the research team (CS, EL, AS, JM and RH) to measure duration and explore the content and

For peer review only - http://bmjopen.bmj.com/site/about/guidelines.xhtml

methodological implications for a future study. An in-depth analysis of the consultations using a conversation analytic informed approach [31] is reported elsewhere [32].

A thematic framework-based analysis was used to analyse the focus groups [33] to assess the acceptability of the goal-setting intervention to patients and GPs and possible future improvements to the goal-setting intervention, training and trial design.

Patient and Public Involvement (PPI)

Two PPI representatives contributed to the design of the research as co-applicants on the initial application for funding (AM and HS) and steering group membership (AM and CG). PPI members contributed to the analysis and interpretation of the results, with one PPI representative reviewing and scoring video consultations using OPTION (RH) and a further two reviewing a selection of video consultation transcripts (AM and CG). Two PPI members reviewed and commented on the manuscript and are co-authors (AM and CG).

For peer review only

Results

Recruitment and retention

Sixty general practices were invited with seven expressing interest and six being recruited (Figure 1). Across the six practices (Table 1), 550 patients met the eligibility criteria and were invited. In total, 52 patients were recruited with 24 belonging to practices randomised to goal-setting and 28 to practices in the control group. Thirteen patients were held in reserve from three practices which had recruited enough patients. The response rate was 12% ((52+13))/550). There was little variation in age, sex and deprivation between those who participated and those who did not (Supplementary Table 1). Two participants in the goal-setting group and five in the control group did not receive the initial consultation because they declined to attend, were unavailable or withdrew consent. Four participants in the goal-setting group did not receive the follow-up consultation because of ill health or death. Data collected directly from participants were available for 18 participants in the goal-setting group and 23 in the control group. Participant data collected from practices were available for 23 participants in the goal-setting group and 28 in the control group. Recruitment of practices took place between December 2016 and February 2017 and recruitment of patients between April and May 2017.

The control practices were in more urbanised areas with larger practice populations and more female GPs participating compared to goal-setting practices (Table 1). The goal-setting group, compared to control (see Table 2), had more patient participants who were female (54% compared to 29%), older (80 years old compared to 77), with a higher number of health problems (5 compared to 4) and medications (13.0 compared to 11.5), but similar quality of life. The control group had participants spread across all four IMD quartiles, whereas the goal-setting group had participants in only the second and third quartiles. All participants were white British and retired, except for one participant in the goal-setting group who was of working age but not employed and one in the control group who was self-employed. There was variation in participant baseline characteristics between practices in mean age (range 69.5 to 85.8 years old), proportion of females (range 25% to 73%), number of medications (range 10.0 to 15.5) and number of health problems (range 3.0 to 7.5) across participating practices.

The mean initial consultation time in the goal-setting group was 23.0 minutes and in the control 19.2 minutes (Table 3). GPs in the intervention group saw a mean number of 4.4 patients (range 4 to 5), whereas GPs in the control group saw a mean of 3.8 patients (range 2 to 7). Patients spoke more in the goal-setting group initial consultation (mean GP:patient word count ratio (WCR) 1.35) than the control group (WCR 1.52). Dyadic OPTION scores for GPs perceptions of shared decision making were not statistically significantly higher in the goal-setting group compared to the control group, and collaboRATE scores were similar. Observer OPTION scores showed large variation and inconsistency in scoring between the three research team members (data not presented).

Most patients set two or three goals (Table 4) in the goal setting intervention arm, with GPs and patients setting on average one more goal in Practice 1 than in Practice 3. The commonest types of goals were related to management of chronic conditions, walking, maintaining social and leisure interests and weight management (Table 5). Forty-two of the 50 goals were scored with a mean attainment score per patient of 1.45 (1= partially attained and 2= as expected) with ‘partially attained’ being the commonest outcome (Table 4).

In the control arm, goals were rarely mentioned. Four usual-care GPs followed the care planning template recommended within the enhanced service for avoiding unplanned admissions [13], one

For peer review only - http://bmjopen.bmj.com/site/about/guidelines.xhtml

GP appeared to treat it as a normal consultation (i.e. problem focused) and another GP focused mainly on end of life issues.

Outcome measures

As expected in this small feasibility study, there were no statistically significant differences between goal-setting and control from baseline to follow-up in PACIC score, health-related quality of life as measured by EQ5D, number of medications or GPCOG score (Table 6 which also shows the intra-class correlation coefficients). Capability as measured by ICECAP-O at six months, improved slightly more in the control group than in the goal-setting group, but the 95% confidence interval included zero (mean difference between groups -0.08, 95% CI -0.15 to -0.00).

There was considerable variation in health care use in the six months prior to randomisation and six months follow-up (Table 7). Most types of health care contact increased in both the control and goal-setting groups, but district nurse contacts increased and inpatient admissions decreased only in the goal-setting group. Quality and Outcomes Framework data were collected at baseline and follow-up, but the results were uninformative due to low numbers and low variability (Supplementary Table 2). There was one death in the goal-setting group due to cancer, which was judged to be unrelated to the intervention. The estimated cost of the goal-setting was £147 per patient, of which £95 related to costs of providing initial and follow-up GP consultations, and £43 related to the cost of GP training. There was a small cost for the study researcher to explain goal-setting. A mean cost of £50 per patient was incurred in the control group for the initial consultation. The single largest cost for the six-months prior to recruitment and the six-months of follow-up was inpatient stays (Table 7). There were also substantial costs in other settings, for example in general practice contacts and district nurse services. The types, number and associated costs of health service use varied considerably, as would be expected in a feasibility study.

Acceptability

Eleven patients expressed interest in the focus group and six were able to attend on the selected date. Two patients who were unable to attend took part in a telephone interview. Of the five GPs who delivered the intervention, four attended the focus group and one was unable to attend, and was subsequently interviewed face-to-face at the GP surgery. All six patient participants attending the focus group reported positive experiences and views of the intervention, particularly regarding the different emphasis of the consultation. Participants spoke of goal setting providing clarity about what mattered to them, and helping them to plan and focus their lives

> *"[Goal-setting] gives he or she a much better understanding of particularly what is worrying you, what your aims are, the things that you miss being able to do and to be able to actually explain it where [GPs] have time, because very often the GPs, you know, you've only got ten minutes. But with these consultations, you're actually able to talk to a doctor, as you would indeed a friend almost" (Patient 107)*

Goal-setting appeared to function as a mechanism for helping make consultations patient-centred. This was reflected in the unanimous support for the intervention amongst the four GPs who attended the GP focus group and one GP who was interviewed by phone. GPs described the goal-setting consultations as *more patient-centred and reflected on its 'therapeutic powers'* (GP10) compared to day-to-day general practice, which GPs felt could be dominated by '*box-ticking*' and '*target driven*' (GP018) medicine.

For peer review only - http://bmjopen.bmj.com/site/about/guidelines.xhtml

> *"I felt almost as if I was trying to put on a different hat, you know, trying not to constantly interrupt them or to sort of sway them in any way, I was trying to give them the opportunity to just say what they wanted to say and set any goal that they wanted to and I, and it made me reflect on actually what I do during the day to day when I've got ten minutes with a patient and I'm very aware of the sort of pressure of, oh I've got to do a medication review and I've got to do this and oh no, their cholesterol's now 7 and oh gosh I've, have my colleagues already spoke to them about this and are they aware of X, Y and Z and actually it was quite nice in a way just take a step back and think, um I don't have to do that with this consultation, let's see what happens when the patient has more control over it" (GP025)*

Patient participants spoke positively about the baseline researcher visit because it helped them understand the study and encouraged them to reflect on what was important. However, when discussing wider implementation across the health service, participants acknowledged that a home visit for each patient may be too costly and alternative provision would be acceptable to most people. Patients were reluctant to receive more paperwork as they felt that it was a burden for some people. When asked by the moderator to consider the acceptability of a group session to introduce people to the study and to the concept of goal-setting, all bar one of the patient participants at the focus group felt this would be acceptable.

Continuity of care was a concern for patient participants. Participants spoke of wanting more follow-up and consistency amongst the health care team in relation to their goals in the future; some participants felt there was a disconnection between the activity of goal setting and their subsequent treatment by staff within the practice. While one person was disappointed not to see their own GP, three were positive about consulting with a different doctor, especially if it was difficult to see their usual GP.

GPs stated that the experiential work, especially role play and skill spotting, was the most useful aspect of training. When discussing delivering training at scale, GPs felt e-training with opportunities to watch '*other people role-play*', would fit in with their busy schedules. In addition, multiple shorter e-training modules, using a '*step-by-step*' approach (GP014) that contributed to continuing professional development, would be attractive to GPs when implementing the intervention more widely.

For peer review only - http://bmjopen.bmj.com/site/about/guidelines.xhtml

Discussion

The process of setting goals in a GP consultation and follow-up over six months was acceptable to patients and unanimously supported by participating GPs. Recruitment and retention of practices and patients was achieved. A wide range of goals was set and, as expected with a feasibility study, there were no statistically significant differences in the main outcomes. Goal setting consultations were a similar length to control consultations. The qualitative findings were that goal-setting helped patients and GPs focus on what was important and supported GPs to deliver more patient-centred care. Patient preparedness, continuity of care and being able to deliver training at scale were important considerations for future studies of goal setting. Data on the number of health problems were uninformative due to low numbers and low variability. Asking GPs in the non-intervention group to undertake a video-recorded usual care planning consultation is likely to have altered practice compared to what would have happened within the enhanced service. An intention-to-treat analysis was undertaken to reduce the impact of protocol violations (e.g. patients not receiving the pre-specified intervention).

A Cochrane review, published in 2015, assessed the effects of personalised care planning (defined as goal-setting and action planning), for adults with long term health conditions compared to usual care [8]. Whilst 19 RCTs were included, all except for one focused on single conditions. The one multiple condition study included patients who had high health care use and focused on care planning, with goal-setting as part of the process, across the wider health care system to reduce unplanned admissions [34]. The authors found an increase in quality of life (measured by SF36) in the intervention compared to control, however with 50% of participants lost to follow-up and an intention to treat analysis not undertaken, there was a possibility of a lost to follow-up bias in favour of the intervention.

A systematic review of randomised and non-randomised studies, published in 2017, looked at collaborative goal-setting or health priority setting for elderly people with a chronic condition or multimorbidity [12]. The authors found that in four of eight intervention studies, multifactorial approaches improved goal-setting or care planning, but the review did not assess health outcomes or quality of life. The authors concluded that future research was needed to determine the “mix of essential elements within a multifactorial intervention to provide recommendations on daily practice”. Our study helps to answer this question by identifying some key requirements of goal-setting in primary care.

This was a feasibility study and the main implications are for the design of a subsequent definitive trial. Our objectives were to assess participant recruitment and retention, the acceptability of a goal-setting intervention to patients and GPs, the training needs of GPs, the content of control consultations, goal-setting and the feasibility of collecting relevant outcome measures.

We set out to recruit six practices, and seven (out of 60 invited) were willing to take part after initial email invitations. Participant recruitment and retention was sufficient overall, but low in one practice (which recruited four out of a target of ten). Reminder letters were not sent, and these may help all practices to recruit larger numbers if required in a future study. Seven participants, five from the control and two from goal-setting, did not receive the initial consultation because they declined the consultation, withdrew consent or were not able to attend. Possibly some were disappointed to be allocated to the control group.

Goal-setting was acceptable to participating patients and GPs, albeit a self-selecting group who were willing to take part in research into goal-setting. Goal setting is unlikely to be relevant to everyone,

For peer review only - http://bmjopen.bmj.com/site/about/guidelines.xhtml

but the positive response of participants in this feasibility study suggests that it is likely to have wider acceptability in general practice. Further research is needed to understand which patients will benefit most from goal setting. The readiness of patients to undertake goal-setting appeared to be important. Although several goals were only partially attained, GPs and patients still felt them to be worthwhile, suggesting that the process of goal setting has benefits, apart from the achievement of goals.

Training participating GPs in goal-setting was important, and participating GPs thought that the face-to-face training with role play used in the feasibility study could be replaced with online e-learning to allow delivery at scale to a wider GP workforce. The initial researcher visit was important to participants and the key elements of this visit would be delivered in a future trial using video and leaflet-based patient information aids, again to be developed using material collected during this feasibility study.

Goal setting consultations were more focussed on what mattered to the patient than the control consultations, and key challenges, which we discuss fully elsewhere, were around preparation and agreeing goals [32]. The concern reported by some patients when their goals were not considered in future health care contacts suggests that better communication of goals with the rest of the health care team will be needed. Planned follow-up of goals with the GP sooner than six months if needed would also improve continuity of care, which is associated with lower mortality [35].

We collected a wide range of outcome measures in order to assess their feasibility and suitability for use in a future trial. Both EQ-5D-5L and the ICECAP-O should be used in a future economic evaluation but would not be the best primary outcome measure for a trial of goal setting. A recent study which aimed to improve the management of patients with multimorbidity, the 3D study, used the EQ5D5L as a primary outcome, but did not find any significant difference between arms [36]. It may be that the domains within the EQ5D5L are insensitive to changes in care for patients with multimorbidity and a measure of patient centred care such as PACIC is a more appropriate primary outcome measure as it contains a sub scale to measure goal setting. Baseline and follow-up data were collected during researcher visits, which could be replaced by postal questionnaires as the amount and complexity of data to be collected would be reduced. Postal questionnaires are widely used in research and could either increase or reduce the completeness of follow-up data, depending on the preference of individuals for a visit rather than a postal form to complete.

Quality and Outcomes Framework data did not prove useful because of the small numbers and low variation. The observer OPTION scoring, initially developed within a rehabilitation context, had low consistency between researchers and therefore was less useful. A possible reason for this lack of consistency was that OPTION was developed for specific clinical decisions, and not for goal setting which often involved multiple complex decisions.

Goal-setting can be valuable for GPs and patients seeking to agree the desired outcomes of care, particularly for older patients with multimorbidity. This study has demonstrated that it is acceptable and feasible in general practice, and a full trial is now needed to assess whether goal setting improves important clinical outcomes for patients.

For peer review only - http://bmjopen.bmj.com/site/about/guidelines.xhtml

Competing interests: None declared.

Author contributions: NS, JF, CS and AS conceived the idea. All authors contributed to the design of the study. EL led the data collection which was supported by TW, IK and SJ. CS, JM and AS led the analysis of the qualitative data which was supported by SW. AC undertook the statistical analysis. DT undertook the economic analysis. CG and AM provided a patient and public perspective and helped with qualitative analysis. AL led the partnership with NHS England and contributed to handling of adverse events. All authors contributed to the interpretation of the results. JF drafted the initial manuscript. All authors revised the manuscript and approved the final version. NS is the guarantor.

Acknowledgements: We thank Rebecca Harmston (RH) for reviewing the video-consultations from the patient and public involvement perspective, Clara Yates and Gosia Majsak-Newman at the Norfolk and Suffolk Primary and Community Care Research Office for their support, and the patients and staff who took part in the study.

Funding statement: This paper presents independent research funded by the National Institute for Health Research (NIHR) under its Research for Patient Benefit (RfPB) Programme (Grant Reference Number PB-PG-0215-36079). The views expressed are those of the author(s) and not necessarily those of the NHS, the NIHR or the Department of Health and Social Care. The funder did not have any role in the design, collection, analysis or interpretation of data or in writing the manuscript.

Data sharing:

Dataset of quantitative data and statistical code is available from the corresponding author.

For peer review only

References

1. Steel N, Ford JA, Newton JN, et al. Changes in health in the countries of the UK and 150 English Local Authority areas 1990 to 2016: a systematic analysis for the Global Burden of Disease Study 2016. *Lancet* 2018;**392**:1647-61 doi: 10.1016/S0140-6736(18)32207-4.
2. Barnett K, Mercer SW, Norbury M, Watt G, Wyke S, Guthrie B. Epidemiology of multimorbidity and implications for health care, research, and medical education: a cross-sectional study. *Lancet* 2012;**380**:37-43 doi: 10.1016/s0140-6736(12)60240-2.
3. Fortin M, Lapointe L, Hudon C, Vanasse A, Ntetu AL, Maltais D. Multimorbidity and quality of life in primary care: a systematic review. *Health and Quality of Life Outcomes* 2004;**2**:51-51 doi: 10.1186/1477-7525-2-51.
4. Wilkie P. Really putting patients first: ensuring significant involvement for patients in healthcare decision making. *Br J Gen Pract* 2015;**65**:108-09 doi: 10.3399/bjgp15X683821.
5. Reuben DB, Tinetti ME. Goal-Oriented Patient Care — An Alternative Health Outcomes Paradigm. *NEMJ* 2012;**366**:777-79 doi: 10.1056/NEJMp1113631.
6. Guthrie B, Payne K, Alderson P, McMurdo MET, Mercer SW. Adapting clinical guidelines to take account of multimorbidity. *BMJ* 2012;**345** doi: 10.1136/bmj.e6341.
7. National Institute for Health and Care Excellence. Multimorbidity: clinical assessment and management NICE guideline [NG56]. London, 2016. https://www.nice.org.uk/guidance/ng56 Accessed on 8 Nov 2018.
8. Coulter A, Entwistle VA, Eccles A, Ryan S, Shepperd S, Perera R. Personalised care planning for adults with chronic or long-term health conditions. *Cochrane Database Syst Rev* 2015:Cd010523 doi: 10.1002/14651858.CD010523.pub2.
9. Reeves D, Hann M, Rick J, et al. Care plans and care planning in the management of long-term conditions in the UK: a controlled prospective cohort study. *Br J Gen Pract* 2014;**64**:e568-75 doi: 10.3399/bjgp14X681385.
10. Burt J, Roland M, Paddison C, et al. Prevalence and benefits of care plans and care planning for people with long-term conditions in England. *J Health Serv Res Policy* 2012;**17 Suppl 1**:64-71 doi: 10.1258/jhsrp.2011.010172.
11. Elwyn G, Laitner S, Coulter A, Walker E, Watson P, Thomson R. Implementing shared decision making in the NHS. *BMJ* 2010;**341**:c5146 doi: 10.1136/bmj.c5146.
12. Vermunt NPCA, Harmsen M, Westert GP, Olde Rikkert MGM, Faber MJ. Collaborative goal setting with elderly patients with chronic disease or multimorbidity: a systematic review. *BMC Geriatr* 2017;**17**:167 doi: 10.1186/s12877-017-0534-0.
13. NHS England. Enhanced service specification: Avoiding unplanned admissions: proactive case finding and patient review for vulnerable people. 2014. https://www.england.nhs.uk/wp-content/uploads/2014/06/avoid-unpln-admss-serv-spec.pdf Accessed on 8 Nov 2018.
14. Steel N, Clark A, Ford J, et al. Goal-setting in care planning for people with multimorbidity: feasibility study and intervention refinement Norwich: University of East Anglia. 2016. http://www.uea.ac.uk/medicine/research/primary-care-and-epidemiology/goalplan Accessed on 8 Nov 2018.
15. Elwyn G, Frosch D, Thomson R, et al. Shared Decision Making: A Model for Clinical Practice. *J Gen Intern Med* 2012;**27**:1361-67 doi: 10.1007/s11606-012-2077-6.
16. Elwyn G, Durand MA, Song J, et al. A three-talk model for shared decision making: multistage consultation process. *BMJ* 2017;**359** doi: 10.1136/bmj.j4891.
17. Silverman J, Kurtz S, Draper J. *Skills for communicating with patients*: CRC Press, 2016.
18. Herdman M, Gudex C, Lloyd A, et al. Development and preliminary testing of the new five-level version of EQ-5D (EQ-5D-5L). *Qual Life Res* 2011;**20**:1727-36 doi: 10.1007/s11136-011-9903-x.
19. Coast J, Flynn TN, Natarajan L, et al. Valuing the ICECAP capability index for older people. *Soc Sci Med* 2008;**67**:874-82 doi: 10.1016/j.socscimed.2008.05.015.

For peer review only - http://bmjopen.bmj.com/site/about/guidelines.xhtml

20. Brodaty H, Pond D, Kemp NM, et al. The GPCOG: a new screening test for dementia designed for general practice. *J Am Geriatr Soc* 2002;**50**:530-4.
21. Glasgow RE, Wagner EH, Schaefer J, Mahoney LD, Reid RJ, Greene SM. Development and validation of the Patient Assessment of Chronic Illness Care (PACIC). *Med Care* 2005;**43**:436-44.
22. NHS Employers. General Medical Services (GMS) contract Quality and Outcomes Framework (QOF). 2014. https://www.england.nhs.uk/gp/gpfv/investment/gp-contract/ Accessed on 8 Nov 2018.
23. Barr PJ, Thompson R, Walsh T, Grande SW, Ozanne EM, Elwyn G. The psychometric properties of CollaboRATE: a fast and frugal patient-reported measure of the shared decision-making process. *J Med Internet Res* 2014;**16**:e2 doi: 10.2196/jmir.3085.
24. Melbourne E, Sinclair K, Durand MA, Legare F, Elwyn G. Developing a dyadic OPTION scale to measure perceptions of shared decision making. *Patient Educ Couns* 2010;**78**:177-83 doi: 10.1016/j.pec.2009.07.009.
25. Turner-Stokes L. Goal attainment scaling (GAS) in rehabilitation: a practical guide. *Clin Rehabil* 2009;**23**:362-70 doi: 10.1177/0269215508101742.
26. Barr PJ, O'Malley AJ, Tsulukidze M, Gionfriddo MR, Montori V, Elwyn G. The psychometric properties of Observer OPTION5, an observer measure of shared decision making. *Patient Educ Couns* 2015;**98**:970-76 doi: https://doi.org/10.1016/j.pec.2015.04.010.
27. Eldridge SM, Costelloe CE, Kahan BC, Lancaster GA, Kerry SM. How big should the pilot study for my cluster randomised trial be? *Stat Methods Med Res* 2016;**25**:1039-56 doi: 10.1177/0962280215588242.
28. Department of Health and Social Care. NHS reference costs 2015 to 2016. London, 2016. https://www.gov.uk/government/publications/nhs-reference-costs-2015-to-2016 Accessed on 8 Nov 2018.
29. Curtis L. Unit Costs of Health and Social Care 2017. Canterbury: University of Kent 2017. https://www.pssru.ac.uk/project-pages/unit-costs/unit-costs-2017/ Accessed on 8 Nov 2018.
30. Drummond MF, Sculpher MJ, Claxton K, Stoddart GL, Torrance GW. *Methods for the economic evaluation of health care programmes*: Oxford University Press, 2015.
31. Drew P, Chatwin J, Collins S. Conversation analysis: a method for research into interactions between patients and health-care professionals. *Health Expect* 2001;**4**:58-70.
32. Salter C, Shiner A, Lenaghan E, et al. Setting goals with patients living with multimorbidity: qualitative analysis of general practice consultations. 2018. Manuscript submitted for publication .
33. Ritchie J, Lewis J, Nicholls CM, Ormston R. *Qualitative research practice: A guide for social science students and researchers*: Sage, 2013.
34. Battersby M, Harvey P, Mills PD, et al. SA HealthPlus: a controlled trial of a statewide application of a generic model of chronic illness care. *Milbank Q* 2007;**85**:37-67 doi: 10.1111/j.1468-0009.2007.00476.x.
35. Pereira Gray DJ, Sidaway-Lee K, White E, Thorne A, Evans PH. Continuity of care with doctors—a matter of life and death? A systematic review of continuity of care and mortality. *BMJ Open* 2018;**8** doi: 10.1136/bmjopen-2017-021161.
36. Salisbury C, Man MS, Bower P, et al. Management of multimorbidity using a patient-centred care model: a pragmatic cluster-randomised trial of the 3D approach. *Lancet* 2018;**392**:41-50 doi: 10.1016/s0140-6736(18)31308-4.
37. Office for National Statistics. 2011 Rural-urban classification. 2011. http://www.ons.gov.uk/ons/guide-method/geography/products/area-classifications/2011-rural-urban/index.html Accessed on 8 Nov 2018.

For peer review only

For peer review only - http://bmjopen.bmj.com/site/about/guidelines.xhtml

Figure legends

Figure 1: Consort flow diagram

For peer review only

For peer review only - http://bmjopen.bmj.com/site/about/guidelines.xhtml

Table 1: Practice characteristics and patient recruitment

	Goal-setting			Control		
	**Practice 1**	**Practice 2**	**Practice 3**	**Practice 4**	**Practice 5**	**Practice 6**
**Practice characteristics**						
Practice rurality*	Village	Town and fringe	Town and fringe	Urban >10K	Urban >10K	Urban >10K
Patient population	5000 to 9,900	10,000 to 14,900	5000 to 9,900	>14,900	10,000 to 14,900	10,000 to 14,900
IMD practice decile	7	5	7	9	5	5
Characteristics of participating GPs	2 x male (partners, 2 x PT)	1 x male, 1 x female (partners, 2 x FT)	1 x male (partner, PT)	1 x male, 1 x female (partners, 1 x FT, 1 x PT)	2 x female (partners, 2 x PT)	2 x female (partners, 2 x PT)
Years qualified of participating GPs	GP014 = >20 yrs; GP018 = 10 to 20 yrs	GP025 = <10 yrs; GP026 = 10 to 20 yrs	GP038 = 10 to 20 yrs	GP046 = >20 yrs; GP047 = >20 yrs	GP053 = >20 yrs; GP055 = >20 yrs	GP061 = 10 to 20 yrs; GP067 = 10 to 20 yrs
**Patient recruitment**						
Patients assessed for eligibility, n	9067	14845	6791	108 (0.6)	124 (1.2)	86 (0.6)
Patients invited, n(%)	77 (0.8)	108 (0.7)	47 (0.7)	8 (7.4)	10 (8.1)	10 (11.6)
Recruited, n(%)**	11 (14.3)	9 (8.3)	4 (8.5)	108 (0.6)	124 (1.2)	86 (0.6)

*ONS indicator 2011 [37], IMD = Index of Multiple Deprivation (1= most deprived and 10 least deprived), partner = GP with responsibility for the practice, FT= full time, PT = part time, n= number, **=does not include those on the reserve list (see Figure 1)

For peer review only

Table 2: Baseline characteristics of patient participants

Variable		Control	Goal-setting
Number		28	24
Female n (%)		11 (39%)	13 (54%)
Age mean (SD)		77.18 (9.42)	80.42 (8.72)
GPCOG category n (%)	Impairment and further investigations implied	1 (4%)	0 (0%)
	Informant interview required	17 (61%)	19 (79%)
	No cognitive impairment	10 (36%)	5 (21%)
Number of diagnoses* median (IQR)		4.00 (3.00, 5.00)	5.00 (3.00, 6.00)
IMD national quartile n (%)	1	5 (18%)	0 (0%)
	2	9 (32%)	14 (58%)
	3	3 (11%)	10 (42%)
	4	11 (39%)	0 (0%)
Marital status n (%)	Divorced	0 (0%)	2 (8%)
	Living with partner	0 (0%)	2 (8%)
	Married	12 (43%)	10 (42%)
	Single	2 (7%)	4 (17%)
	Widowed	14 (50%)	6 (25%)

N= number, SD = standard deviation, IQR = Interquartile Range, GPCOG = General Practitioner assessment of Cognition, PACIC = Patients Assessment Chronic Illness Care, EQ-5Q-5L = 5 level EQ-5D, ICECAP-O = ICEpop CAPability measure for Older people, * = based on Barnett list [2], IMD = Index of Multiple Deprivation

For peer review only

Table 3: Characteristics of initial consultations

	Intervention group				Control group				Mean difference between intervention and control (95% CI)
	Practice 1 (n = 10)	Practice 2 (n = 8)	Practice 3 (n = 4)	Intervention total (n = 22)	Practice 4 (n = 7)	Practice 5 (n = 9)	Practice 6 (n = 7)	Control total (n = 23)	
Duration of initial consultation (mins) mean (SD)	24.1 (4.0)	23.3 (4.4)	19.9 (6.2)	23.0 (4.6)	14.3 (4.8)	25.2 (5.7)	16.3 (4.1)	19.2 (6.9)	3.88 (-3.25,11.01)
Dyadic OPTION scores mean (SD)	65.3 (9.0)	63.2 (6.4)	62.5 (3.6)	64.0 (7.2)	63.5 (13.0)	62.7 (4.0)	42.1 (20.4)	56.6 (16.2)	7.57 (-6.37,21.50)
CollaboRATE scores mean (SD)	7.8 (1.0)	8.5 (0.9)	8.8 (0.2)	8.2 (1.0)	7.0 (2.6)	8.6 (0.7)	8.7 (0.6)	8.1 (1.8)	0.20 (-1.06,1.47)
GP:patient word count ratio mean (SD)	1.23 (0.40)	1.41 (0.78)	1.50 (1.05)	1.35 (0.67)	1.13 (0.45)	1.92 (0.75)	1.39 (0.52)	1.52 (0.67)	-0.14 (-0.65,0.37)

SD= standard deviation, n= number, 95%CI = 95% confidence interval

For peer review only

For peer review only - http://bmjopen.bmj.com/site/about/guidelines.xhtml

Table 4: Number of goals set and goal attainment score

		Practice 1 (n = 10)	Practice 2 (n = 8)	Practice 3 (n = 4)	Total
Goals set		27	16	7	50
Number of goal per patient	1 goal set	0	2	1	3
	2 goals set	3	4	3	10
	3 goals set	7	2	0	9
Number of goals with data available for attainment scoring		21	15	6	42
Mean score of goal attainment per patient		1.43	1.67	1.0	1.45
Goal attainment n (%)	worse than expected	1 (4.8)	2 (13.3)	1 (16.7)	4 (9.5)
	no change	4 (19.0)	0 (0.0)	2 (33.3)	6 (14.3)
	partially attained	9 (42.9)	5 (33.3)	1 (16.7)	15 (35.7)
	as expected	2 (9.5)	3 (20.0)	1 (16.7)	6 (14.3)
	a little more	2 (9.5)	4 (26.7)	0 (0.0)	6 (14.3)
	a lot more than expected	3 (14.3)	1 (6.7)	1 (16.7)	5 (11.9)

n = number of participants

For peer review only

For peer review only - http://bmjopen.bmj.com/site/about/guidelines.xhtml

Table 5: Categories of goals set

Goal categories	Number of goals
Management of chronic condition (non-medication)	9
Walking-related	8
Maintain interests	5
Management of chronic condition (medication-related)	5
Gain weight	4
Social participation	3
Healthy living	3
Balance/mobility	3
Gardening-related	3
Manual dexterity	3
Mental health	2
End of life management	1
Cooking/food preparation	1
Grand Total	50

For peer review only

For peer review only - http://bmjopen.bmj.com/site/about/guidelines.xhtml

Table 6: Change in outcome measures between groups at six months

Variable	Control				Intervention				Mean difference-in-difference between goal-setting and control (95% CI)	Intraclass correlation coefficient (95% CI)
	n	Baseline	Follow-up	Difference	n	Baseline	Follow-up	Difference		
Number of medication	28	12.5 (8.19)	12.79 (7.25)	0.29 (2.65)	23	13.61 (4.56)	14.65 (4.44)	1.04 (3.21)	0.76 (-0.85,2.37)	Not estimated
GPCOG	23	7.35 (1.70)	6.78 (2.19)	-0.57 (2.02)	19	7.58 (1.30)	7.00 (2.26)	-0.58 (2.29)	0.09 (-1.65,1.84)	0.08 (0.00,0.77)
PACIC	23	1.45 (0.30)	1.85 (0.77)	0.40 (0.69)	18	1.94 (0.76)	2.25 (0.70)	0.31 (0.98)	-0.09 (-0.60,0.42)	Not estimated
EQ-5D-5L	23	0.54 (0.34)	0.52 (0.35)	-0.02 (0.19)	18	0.56 (0.25)	0.55 (0.28)	-0.01 (0.15)	0.02 (-0.11,0.13)	0.05 (0.00,0.94)
ICECAP-O	22	0.72 (0.26)	0.78 (0.20)	0.06 (0.14)	17	0.78 (0.12)	0.77 (0.13)	-0.02 (0.06)	-0.08 (-0.15,-0.00)	Not estimated

SD = standard deviation, GPCOG = General Practitioner assessment of Cognition, PACIC = Patients Assessment Chronic Illness Care, EQ-5Q-5L = 5 level EQ-5D, ICECAP-O = ICEpop CAPability measure for Older people, 95%CI = 95% confidence interval

For peer review only

Table 7: Costs associated with health care use

Resource use	6-months prior to recruitment: Control Total contacts n	Control Total cost £	Control Mean cost £ (SD)	Goal-setting Total contacts n	Goal-setting Total cost £	Goal-setting Mean cost £ (SD)	Recruitment to 6-month follow-up: Control Total contacts n	Control Total cost £	Control Mean cost £ (SD)	Goal-setting Total contacts n	Goal-setting Total cost £	Goal-setting Mean cost £ (SD)
Community based services												
GP	157	4,636	166 (164)	89	2,464	107 (115)	177	5,150	184 (150)	124	4,002	174 (145)
Other practice based	97	922	33 (42)	108	1,080	47 (30)	152	1,823	65 (58)	149	1,529	66 (53)
District Nurse	148	3,582	128 (546)	198	6,450	280 (1297)	100	2,879	103 (321)	241	7,450	324 (1384)
Other	72	1,434	51 (132)	72	2,601	113 (193)	189	7,652	273 (355)	97	5,510	240 (224)
All community based	474	10,575	378 (778)	467	12,594	548 (1520)	618	15,681	560 (719)	611	16,962	737 (1537)
Inpatient	4	11,291	403 (1113)	16	28,054	1220 (2584)	12	35,055	1252 (2203)	13	39,889	1734 (4815)
Outpatient	45	4,848	173 (208)	51	7,381	321 (397)	41	4,424	158 (202)	52	6,295	274 (329)
A&E	1	138	5 (26)	6	826	36 (74)	15	2,066	74 (109)	16	2,204	96 (128)
Total for all costs		26,853	959 (1776)		48,856	2124 (4031)		57,226	2044 (2665)		65,349	2841 (4968)

SD = standard deviation, A&E = Accident and Emergency

For peer review only - http://bmjopen.bmj.com/site/about/guidelines.xhtml

For peer review only

For peer review only - http://bmjopen.bmj.com/site/about/guidelines.xhtml

Figure 1: Consort flow diagram

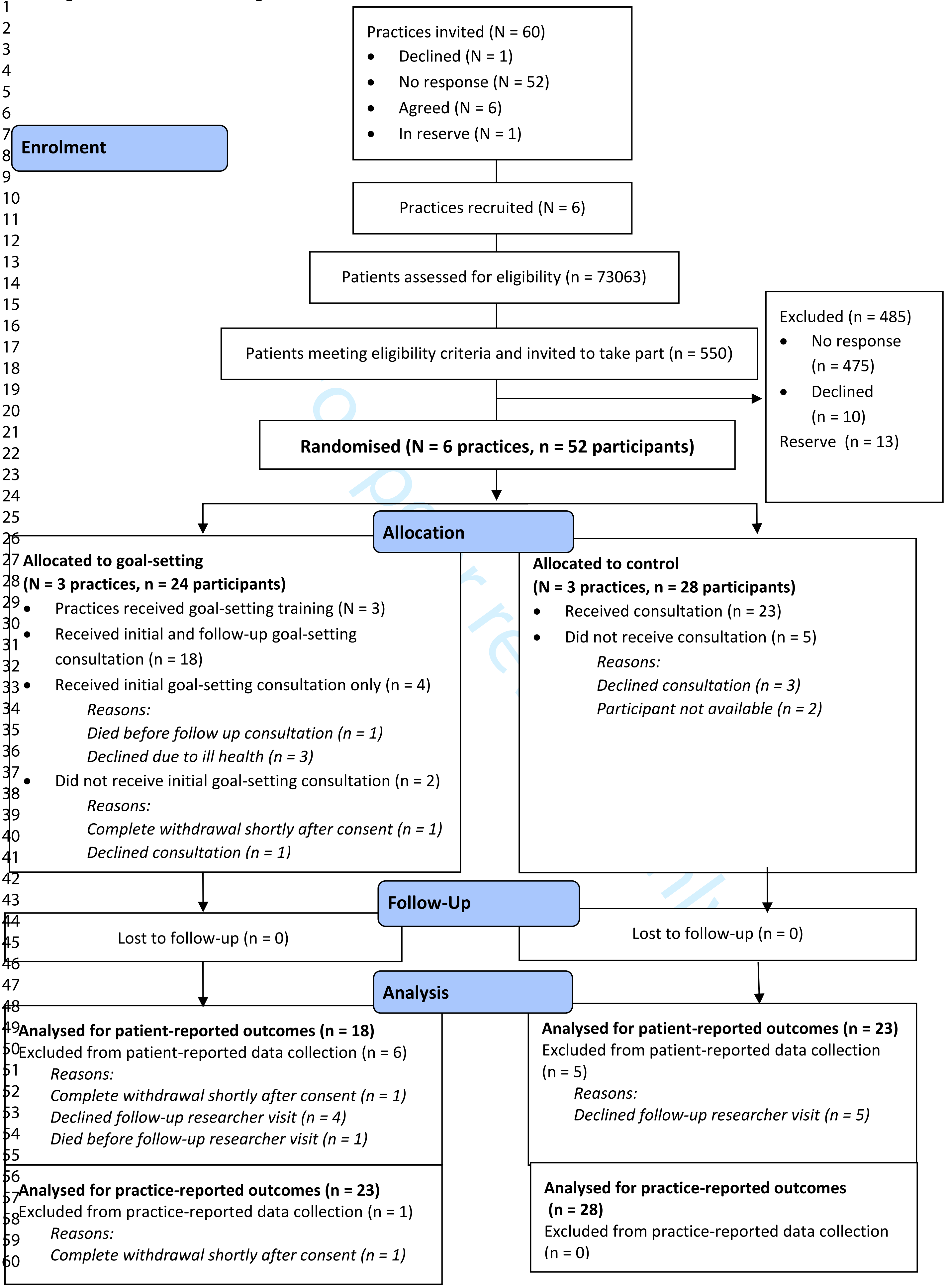

(N= number of practice, n= number of individuals)

For peer review only - http://bmjopen.bmj.com/site/about/guidelines.xhtml

Supplementary Table 1: Characteristics of those who participated compared with those who did not

	Participation	Non-participation
Number	52	498
Age mean (SD)	78.5 (9.0)	79.6 (12.2)
Female %	46.2%	53.8%
IMD decile mean (SD)	5.8 (2.3)	5.3 (2.2)

SD = standard deviation, IMD = Index of Multiple Deprivation

For peer review only

Supplementary Table 2: Quality and Outcomes Framework data

		Goal-setting	Control
BMI	No of participants	2	3
	Baseline mean (SD)	28.4 (1.9)	37.8 (8.0)
	Follow-up (mean, SD)	28.5 (3.6)	37.0 (9.5)
	Diff (mean, SD)	0.1 (1.7)	-0.8 (2.7)
BP, mmHg	No of participants	5	5
	Baseline systolic (mean, SD)	133.5 (6.5)	127.5 (19.0)
	Baseline diastolic (mean, SD)	70.7 (4.4)	69.2 (5.5)
	Follow-up systolic (mean, SD)	144.7 (7.0)	124.4 (6.2)
	Follow-up diastolic (mean, SD)	80.6 (4.7)	67.1 (5.6)
	Mean diff systolic (mean, SD)	11.2 (12.6)	-3.1 (14.4)
	Mean diff diastolic (mean, SD)	9.9 (3.1)	-2.1 (8.7)
	Baseline Qof target met (150/90)	5/5	5/5
	Follow-up Qof target met (150/90)	4/5	5/5
eGRFR, mL/min/1,73m^2	No of participants	4	6
	Baseline (mean, SD)	54 (14)	57 (24)
	Follow-up (mean, SD)	56 (17)	59 (25)
	Mean diff (mean, SD)	2 (5)	2 (3)
HB1Ac, mmol/mol	No of participants	1	3
	Baseline (mean, SD)	80 (NA)	39 (3)
	Follow-up (mean, SD)	87 (NA)	43 (6)
	Mean diff (mean, SD)	7 (NA)	4 (3)
	Baseline Qof target met - Diabetes and HB1Ac <59	0/1	1/1
	Baseline Qof target met - Diabetes and HB1Ac <64	0/1	1/1
	Baseline Qof target met - Diabetes and HB1Ac <75	0/1	1/1
	Follow-up Qof target met - Diabetes and HB1Ac <59	0/1	1/1
	Follow-up Qof target met - Diabetes and HB1Ac <64	0/1	1/1
	Follow-up Qof target met - Diabetes and HB1Ac <75	0/1	1/1
Total cholesterol, mg/dL	No of participants	2	1
	Baseline (mean, SD)	2.8 (0.7)	4.2 (NA)
	Follow-up (mean, SD)	3.9 (1.1)	4.9 (NA)
	Mean diff (mean, SD)	1.1 (0.5)	0.7 (NA)
HDL cholesterol, mg/dL	No of participants	2	0
	Baseline (mean, SD)	0.84 (0.19)	NA
	Follow-up (mean, SD)	1.01 (0.4)	NA
	Mean diff (mean, SD)	0.17 (0.15)	NA

BMI = body mass index, SD= standard deviation, BP = blood pressure, eGFR = estimated glomerular filtration rate, HB1Ac = glycated haemoglobin, Qof = Quality and Outcomes Framework, HDL = High Density Lipoproteins, NA= not applicable

For peer review only

University of East Anglia

GoalPlan Study:
Goal-setting form
for completion before care plan appointment

Part 1) What are your goals? What is important to you?

Write down what you would really like to do or achieve over the next 6 months, even if you think it may not be related to your health. Think about things that you would like to do in your personal, home, work, and social life—things that you ***need*** to do, ***want*** to do and / or ***enjoy doing***. Then list them in order of priority— starting with 1 for the goal that matters to you most and that you would like to focus on at the moment. To help you, we have put a completed example overleaf.

What do you really want to achieve over the next 6 months? (your goals)

For peer review only

Example

- *I want to be able to do more in the garden as it relaxes me*
- *I want to lose weight so I can walk more confidently at my daughter's wedding and fit into my favourite outfit again*
- *I want to be able to get more exercise as it used to help me sleep well*
- *I want to get back to driving so I can visit my friend at his house every week*
- *I want to be able to go out with friends once a week again for lunch*

Part 2) Why are these goals important to you?

We now ask you to focus on your goals from Part 1, and to think about why they are important to you. Below is an example of how to find out if a goal you have come up with is important for its own sake, or if it is important because it will help you achieve something else. We ask you to do this exercise with up to three of your goals overleaf.

Part 3) What are the first steps you would like to take towards achieving this goal or goals?

Having identified your most important goals, the final step on this form is to start thing about steps you would like to take to achieve those goals. This is also included in the example below. Please do the same for your goals overleaf.

EXAMPLE GOAL 1: *I want to be able to do more in the garden*

Why are these goals important to you? →

Gardening is my hobby and gives me pleasure. It is my main form of exercise.

What first steps can I take towards achieving this goal? →

Discuss changes to medicines with GP – might reducing medicines help me be more active?

For peer review only - http://bmjopen.bmj.com/site/about/guidelines.xhtml

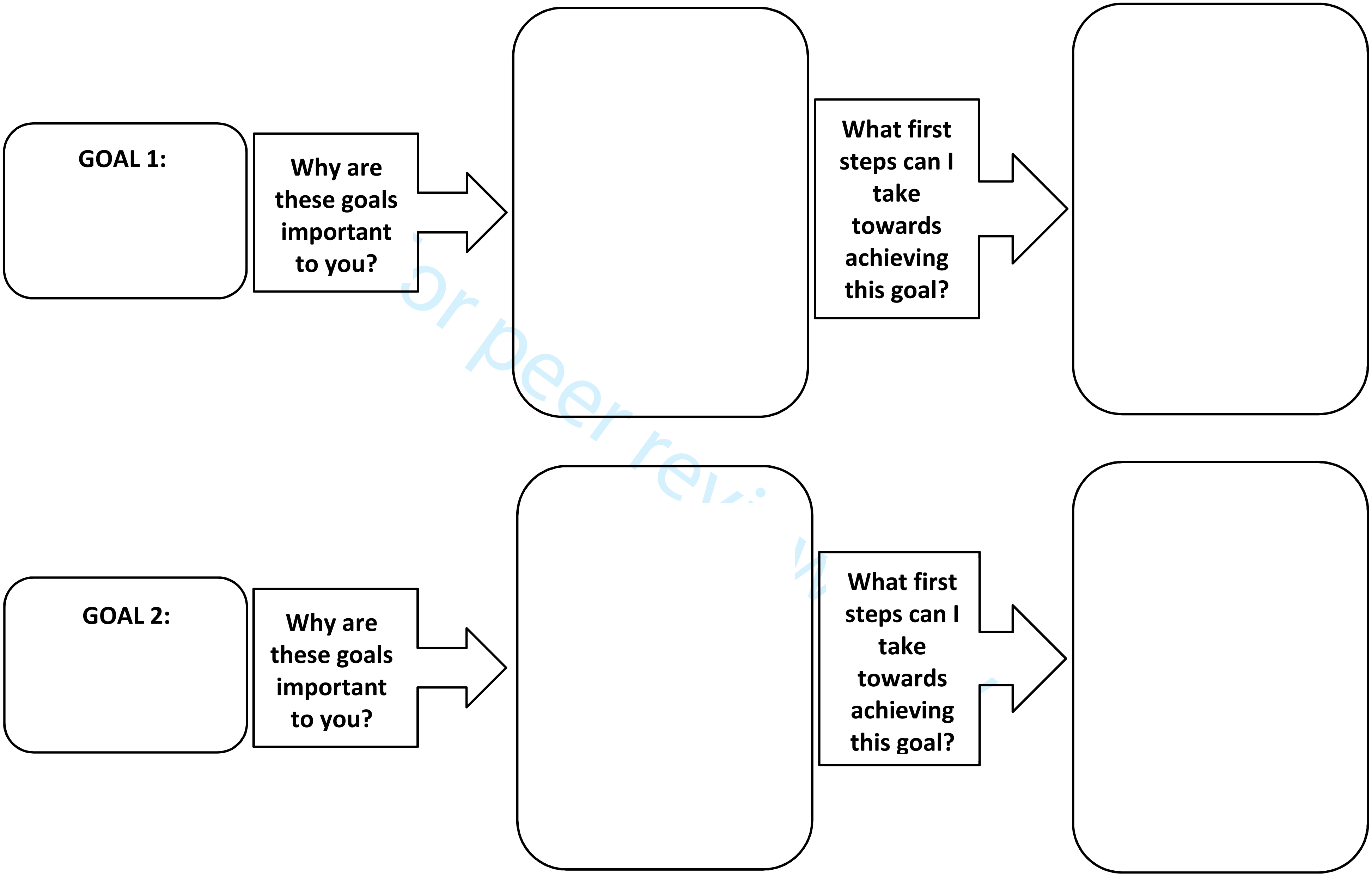

For peer review only - http://bmjopen.bmj.com/site/about/guidelines.xhtml

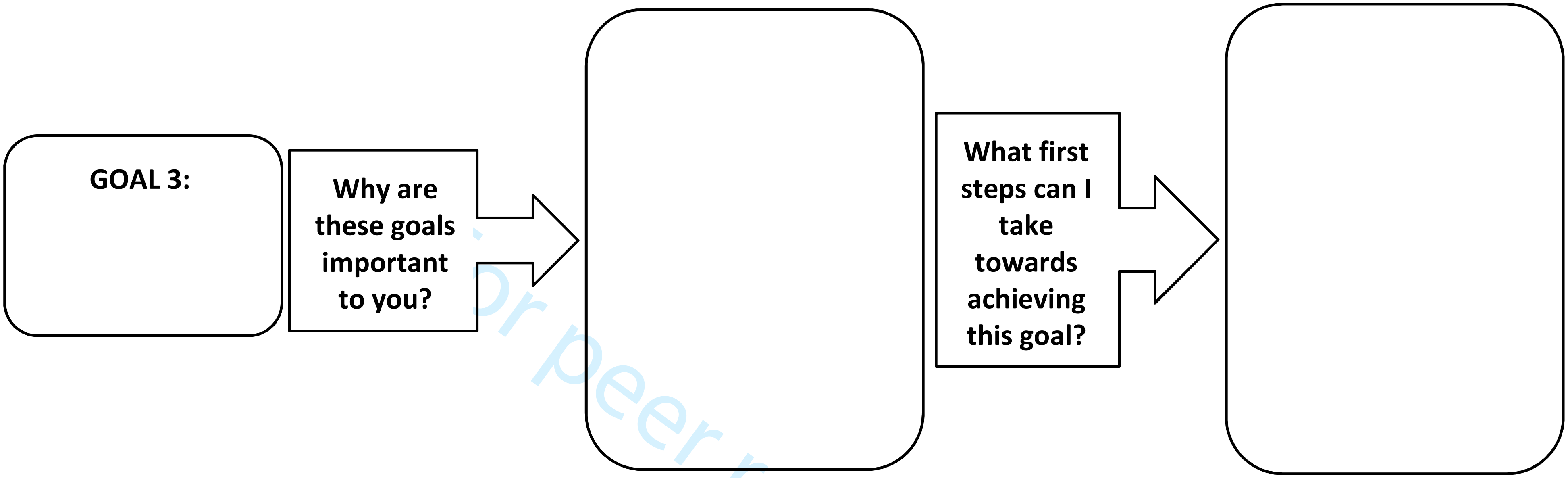

Thank you for completing this goal-setting form. Please take it with you to your care plan appointment. Your GP will discuss it with you and together you will make specific plans to achieve your goals.

For peer review only - http://bmjopen.bmj.com/site/about/guidelines.xhtml

University of East Anglia

GoalPlan Study

GAS-Light verbal scoring system

**Goal 1:**			Tick
**At Baseline**	With respect to this goal do they have?	Some attainment	☐
		No attainment (as bad as they could be)	☐
**At 6 month review:** Was the goal attained?	**Yes**	A lot more	☐
		A little more	☐
		As expected	☐
	**No**	Partially attained	☐
		No change	☐
		Got worse	☐

For peer review only

PTO for further goals

For peer review only - http://bmjopen.bmj.com/site/about/guidelines.xhtml

**Goal 2:**			Tick
**At Baseline**	With respect to this goal do they have?	Some attainment	☐
		No attainment (as bad as they could be)	☐
**At 6 month review:** Was the goal attained?	**Yes** →	A lot more	☐
		A little more	☐
		As expected	☐
	**No** →	Partially attained	☐
		No change	☐
		Got worse	☐

PTO for further goals

For peer review only

For peer review only - http://bmjopen.bmj.com/site/about/guidelines.xhtml

**Goal 3:**			Tick
**At Baseline**	With respect to this goal do they have?	Some attainment	☐
		No attainment (as bad as they could be)	☐
**At 6 month review:** Was the goal attained?	**Yes**	A lot more	☐
		A little more	☐
		As expected	☐
	**No**	Partially attained	☐
		No change	☐
		Got worse	☐

For peer review only

For peer review only - http://bmjopen.bmj.com/site/about/guidelines.xhtml

CONSORT 2010 checklist of information to include when reporting a pilot or feasibility trial*

Section/Topic	Item No	Checklist item	Reported on page No
**Title and abstract**			
	1a	Identification as a pilot or feasibility randomised trial in the title	1
	1b	Structured summary of pilot trial design, methods, results, and conclusions (for specific guidance see CONSORT abstract extension for pilot trials)	2
**Introduction**			
Background and objectives	2a	Scientific background and explanation of rationale for future definitive trial, and reasons for randomised pilot trial	4
	2b	Specific objectives or research questions for pilot trial	4
**Methods**			
Trial design	3a	Description of pilot trial design (such as parallel, factorial) including allocation ratio	5
	3b	Important changes to methods after pilot trial commencement (such as eligibility criteria), with reasons	5
Participants	4a	Eligibility criteria for participants	5
	4b	Settings and locations where the data were collected	5
	4c	How participants were identified and consented	5
Interventions	5	The interventions for each group with sufficient details to allow replication, including how and when they were actually administered	5-6
Outcomes	6a	Completely defined prespecified assessments or measurements to address each pilot trial objective specified in 2b, including how and when they were assessed	6
	6b	Any changes to pilot trial assessments or measurements after the pilot trial commenced, with reasons	5
	6c	If applicable, prespecified criteria used to judge whether, or how, to proceed with future definitive trial	NA
Sample size	7a	Rationale for numbers in the pilot trial	5
	7b	When applicable, explanation of any interim analyses and stopping guidelines	NA
Randomisation:			
Sequence generation	8a	Method used to generate the random allocation sequence	5
	8b	Type of randomisation(s); details of any restriction (such as blocking and block size)	5
Allocation concealment mechanism	9	Mechanism used to implement the random allocation sequence (such as sequentially numbered containers), describing any steps taken to conceal the sequence until interventions were assigned	5

For peer review only

Implementation	10	Who generated the random allocation sequence, who enrolled participants, and who assigned participants to interventions	5
Blinding	11a	If done, who was blinded after assignment to interventions (for example, participants, care providers, those assessing outcomes) and how	NA
	11b	If relevant, description of the similarity of interventions	NA
Statistical methods	12	Methods used to address each pilot trial objective whether qualitative or quantitative	6
**Results**			
Participant flow (a diagram is strongly recommended)	13a	For each group, the numbers of participants who were approached and/or assessed for eligibility, randomly assigned, received intended treatment, and were assessed for each objective	Figure 1
	13b	For each group, losses and exclusions after randomisation, together with reasons	Figure 1
Recruitment	14a	Dates defining the periods of recruitment and follow-up	6
	14b	Why the pilot trial ended or was stopped	NA
Baseline data	15	A table showing baseline demographic and clinical characteristics for each group	Table 3
Numbers analysed	16	For each objective, number of participants (denominator) included in each analysis. If relevant, these numbers should be by randomised group	Tables 4, 5 and 7
Outcomes and estimation	17	For each objective, results including expressions of uncertainty (such as 95% confidence interval) for any estimates. If relevant, these results should be by randomised group	Table 7
Ancillary analyses	18	Results of any other analyses performed that could be used to inform the future definitive trial	NA
Harms	19	All important harms or unintended effects in each group (for specific guidance see CONSORT for harms)	9
	19a	If relevant, other important unintended consequences	NA
**Discussion**			
Limitations	20	Pilot trial limitations, addressing sources of potential bias and remaining uncertainty about feasibility	11-12
Generalisability	21	Generalisability (applicability) of pilot trial methods and findings to future definitive trial and other studies	11-12
Interpretation	22	Interpretation consistent with pilot trial objectives and findings, balancing potential benefits and harms, and considering other relevant evidence	11-12
	22a	Implications for progression from pilot to future definitive trial, including any proposed amendments	11-12
**Other information**			
Registration	23	Registration number for pilot trial and name of trial registry	1
Protocol	24	Where the pilot trial protocol can be accessed, if available	1
Funding	25	Sources of funding and other support (such as supply of drugs), role of funders	13
	26	Ethical approval or approval by research review committee, confirmed with reference number	5

For peer review only

Citation: Eldridge SM, Chan CL, Campbell MJ, Bond CM, Hopewell S, Thabane L, et al. CONSORT 2010 statement: extension to randomised pilot and feasibility trials. BMJ. 2016;355.
*We strongly recommend reading this statement in conjunction with the CONSORT 2010, extension to randomised pilot and feasibility trials, Explanation and Elaboration for important clarifications on all the items. If relevant, we also recommend reading CONSORT extensions for cluster randomised trials, non-inferiority and equivalence trials, non-pharmacological treatments, herbal interventions, and pragmatic trials. Additional extensions are forthcoming: for those and for up to date references relevant to this checklist, see www.consort-statement.org.

For peer review only

CONSORT 2010 checklist of information to include when reporting a pilot or feasibility randomized trial in a journal or conference abstract

Item	Description	Reported on line number
Title	Identification of study as randomised pilot or feasibility trial	1
Authors *	Contact details for the corresponding author	1
Trial design	Description of pilot trial design (eg, parallel, cluster)	2
Methods		
Participants	Eligibility criteria for participants and the settings where the pilot trial was conducted	2
Interventions	Interventions intended for each group	2
Objective	Specific objectives of the pilot trial	2
Outcome	Prespecified assessment or measurement to address the pilot trial objectives**	2
Randomization	How participants were allocated to interventions	2
Blinding (masking)	Whether or not participants, care givers, and those assessing the outcomes were blinded to group assignment	2
Results		
Numbers randomized	Number of participants screened and randomised to each group for the pilot trial objectives**	2
Recruitment	Trial status†	
Numbers analysed	Number of participants analysed in each group for the pilot objectives**	2
Outcome	Results for the pilot objectives, including any expressions of uncertainty**	2
Harms	Important adverse events or side effects	2
Conclusions	General interpretation of the results of pilot trial and their implications for the future definitive trial	2
Trial registration	Registration number for pilot trial and name of trial register	1
Funding	Source of funding for pilot trial	13

Citation: Eldridge SM, Chan CL, Campbell MJ, Bond CM, Hopewell S, Thabane L, et al. CONSORT 2010 statement: extension to randomised pilot and feasibility trials. BMJ. 2016;355.

**this item is specific to conference abstracts*

***Space permitting, list all pilot trial objectives and give the results for each. Otherwise, report those that are a priori agreed as the most important to the decision to proceed with the future definitive RCT.*

†For conference abstracts.

BMJ Open

Can goal-setting for patients with multimorbidity improve outcomes in primary care?: cluster randomised feasibility trial

Journal:	*BMJ Open*
Manuscript ID	bmjopen-2018-025332.R2
Article Type:	Research
Date Submitted by the Author:	08-Mar-2019
Complete List of Authors:	Ford, John; University of East Anglia, Faculty of Medicine and Health Sciences<br>Lenaghan, E; University of East Anglia, Faculty of Medicine and Health Sciences<br>Salter, Charlotte; University of East Anglia, Faculty of Medicine and Health Sciences<br>Turner, David; University of East Anglia, Faculty of Medicine and Health Sciences<br>Shiner, A.; University of East Anglia, Faculty of Medicine and Health Sciences<br>Clark, Allan; University of East Anglia, Faculty of Medicine and Health Sciences<br>Murdoch, Jamie; University of East Anglia, Faculty of Medicine and Health Sciences<br>Green, Carole<br>James, Sarah; University of East Anglia, Faculty of Medicine and Health Sciences<br>Koopmans, Imogen; University of East Anglia, Faculty of Medicine and Health Sciences<br>Lipp, Alistair; NHS England Midlands & East,, West Wing<br>Moseley, Annie<br>Wade, Tom; University of East Anglia, Faculty of Medicine and Health Sciences<br>Winterburn, Sandra; University of East Anglia, Faculty of Medicine and Health Sciences<br>Steel, Nicholas; University of East Anglia, Faculty of Medicine and Health Sciences
**Primary Subject Heading**:	Public health
Secondary Subject Heading:	General practice / Family practice
Keywords:	PRIMARY CARE, HEALTH SERVICES ADMINISTRATION & MANAGEMENT, SOCIAL MEDICINE

For peer review only - http://bmjopen.bmj.com/site/about/guidelines.xhtml

Title

Can goal-setting for patients with multimorbidity improve outcomes in primary care?: cluster randomised feasibility trial

Authors

John A. Ford[1], Elizabeth Lenaghan[1], Charlotte Salter[1], David A. Turner[1], Alice Shiner[1], Allan Clark[1], Jamie Murdoch[1], Carole Green[2]*, Sarah James[1]*, Imogen Koopmans[1]*, Alistair Lipp[3]*, Annie Moseley[2]*, Tom Wade[1]*, Sandra Winterburn[1]*, Nicholas Steel[1]

*Authors listed alphabetically

[1]Faculty of Medicine and Health Sciences, University of East Anglia, Norwich, UK

[2] Patient and Public Involvement Representative, Norwich, UK

[3] NHS England

Corresponding author

Prof Nicholas Steel

Norwich Medical School
University of East Anglia
Chancellor's Drive
Norwich
NR4 7TJ
n.steel@uea.ac.uk
01603 591161

Trials registration:
Title: Goal-setting in care planning for people with multimorbidity
Trial ID: ISRCTN13248305
Date registered: 21/12/2016
Link: http://www.isrctn.com/ISRCTN13248305

Protocol: Available at:
http://www.uea.ac.uk/documents/246046/14839702/GoalPlan+Research+Protocol+v1.2+170316.pdf/86549c5b-c4ed-435b-8719-4c3160f9793f

Word count: 4173

Keywords: goals, multimorbidity, primary health care, patient-centred care, randomized controlled trial

For peer review only - http://bmjopen.bmj.com/site/about/guidelines.xhtml

Abstract

Introduction

Goal-setting is recommended for patients with multimorbidity, but there is little evidence to support its use in general practice.

Objective

To assess the feasibility of goal-setting for patients with multimorbidity, before undertaking a definitive trial.

Design and setting

Cluster-randomised controlled feasibility trial of goal-setting compared to control in six general practices.

Participants

Adults with 2 or more long term health conditions and at risk of unplanned hospital admission.

Interventions

General Practitioners (GPs) underwent training and patients were asked to consider goals before an initial goal-setting consultation and a follow-up consultation six months later. The control group received usual care planning.

Outcome measures

Health-related quality of life (EQ5D5L), capability (ICEpop CAPability measure for Older people (ICECAP-O)), patient assessment of chronic illness care (PACIC) and health care use. All consultations were video or audio-recorded, and focus groups were held with participating GPs and patients.

Results

Fifty-two participants were recruited with a response rate of 12%. Full follow-up data were available for 41. In the goal-setting group, mean age was 80.4 years 54% were female and the median number of prescribed medications was 13, compared to 77.2 years, 39% female and 11.5 medications in the control group. The mean initial consultation time was 23.0 minutes in the goal-setting group and 19.2 in the control group. Overall 28% of patient participants had no cognitive impairment. Participants set between one and three goals on a wide range of subjects, such as chronic disease management, walking, maintaining social and leisure interests, and weight management. Patient participants found goal-setting acceptable and would have liked more frequent follow-up. GPs unanimously liked goal-setting, felt it delivered more patient-centred care and highlighted the importance of training.

Conclusions

This goal-setting intervention was feasible to deliver in general practice. A larger, definitive study is needed to test its effectiveness.

For peer review only

Strengths and limitations of this study

- General practitioners and patients with multimorbidities both benefit from preparation before setting goals
- Recruitment reached target levels in five of six practices, but the patient response rate of 12% means that a definitive study will need sufficient numbers of patients with multimorbidity
- Existing measures of patient centred care are usually designed for a single specific treatment decision and were difficult to apply to goal setting consultations, where several goals were discussed
- The most relevant outcome measure for goal setting was the patient assessment of chronic illness care (PACIC), which includes a sub-scale for goal setting
- Qualitative data from video-recorded consultations and focus groups were vital to understand how goal-setting was implemented in practice, and how acceptable it was to GPs and patients.

For peer review only

For peer review only - http://bmjopen.bmj.com/site/about/guidelines.xhtml

Introduction

The rising number of long-term conditions and prescribed medications has increased the burden of treatment for patients [1 2]. People with multimorbidity (defined as two or more chronic conditions [2]) tend to have a lower quality of life and worse health than those with single conditions [3]. Medical outcomes that work well for relatively healthy patients (e.g. blood pressure control, or disease-free survival) may be inappropriate for patients with multimorbidity or severe disability [4 5], and the use of current single-disease guidelines in this group can encourage harmful polypharmacy with resulting drug-drug and drug-disease interactions [6].

The National Institute for Health and Care Excellence (NICE) recommends an approach to care that takes account of multimorbidity by establishing patient goals, values and priorities [7]. Goal setting is the sharing of realistic goals by health professionals and patients and agreement of the best course of action [8]. Goal setting enables patients and doctors to focus health care on the outcomes that are most important to the patient. Examples of outcomes that matter to patients may include maintaining independence, undertaking paid or voluntary work, preventing adverse outcomes (e.g. falls) and reducing treatment burden [7]. Despite the recommendation that health professionals should establish patient goals with individuals with multimorbidity, there is little evidence to support the use of goal-setting between general practitioners and patients, and it is rarely used in primary care [8-10]. The goal setting approach is more likely to be effective if it incorporates shared decision making, the process by which health professionals and patients make decisions together based on the best available evidence [11], because the goals and actions agreed will be more patient-centred leading to greater engagement in the process by patients. The difference is that shared decision making is usually concerned with specific clinical treatment decisions, whereas goal setting usually involves a wider discussion around ways to deliver outcomes that matter to the patient.

Goal-setting should be, but rarely is, an important element of the care planning process in the UK. For the purposes of this study, we define care planning as ‘a conversation in which patients and clinicians agree on goals and actions for managing the patient's conditions’ [8]. For patients with long term health conditions, personalised care planning has been found to improve physical and psychological health, in addition improving capability to self-manage, compared to usual care [8]. A recent systematic review highlighted the need for evidence exploring ’the effects of personalised care planning on goal-attainment, especially patient’s personal goals as opposed to goals determined by clinicians or researchers‘ [12].

Our goal-setting intervention was designed within the context of a national recommendation that the top 2% of patients at risk of unplanned hospital admission should have a care plan [13]. We wanted to find out if a consultation focussed on goal-setting would improve outcomes for this patient group, compared to control consultations (the usual care planning process undertaken in UK primary care which rarely includes goal setting). Before we could conduct a full trial to answer this question, we needed to answer questions about the feasibility of such a trial. We aimed to assess the feasibility of goal-setting for patients with multimorbidity, at high risk of hospital admission and eligible for a care planning consultation, with a view to undertaking a future definitive randomised controlled trial. Our objectives were to assess participant recruitment and retention, the acceptability of a goal-setting intervention to patients and GPs, the training needs of GPs, the content of control consultations, goal-setting and the feasibility of collecting relevant outcome measures.

For peer review only

For peer review only - http://bmjopen.bmj.com/site/about/guidelines.xhtml

Methods

We undertook a cluster randomised controlled feasibility trial of goal-setting compared to usual care in six general practices in the United Kingdom, with six months follow-up. Six months was long enough for patients and GPs to work towards the agreed goals, but not so long that the goals would have been forgotten. There were no significant changes to the protocol [14]. Research ethics approval was obtained from the NHS Research Ethics Committee (16/EM/0411). Participants were recruited between April and May 2017 and follow-up completed in February 2018.

General practices were invited via two emails through the East of England Clinical Research Network and recruited on a first-come first-served basis. To be eligible, practices had to be using risk stratification to identify patients at high risk of unplanned admission (for example by participating in the Avoiding Unplanned Admissions Enhanced Service: proactive case finding and patient review for vulnerable people [13]), have at least one Good Clinical Practice trained GP and nurse, be able to nominate two GPs to attend the goal-setting training and not be a single handed practice. Practices were reimbursed for staff time and travel to undertake the research and deliver the intervention. Patients were eligible if they were aged 18 or over, identified as in the top 2% for risk of unplanned admission and diagnosed with at least two of 40 morbidities in Barnett's analysis of multimorbidity [2].Patients were excluded if they were deemed to be unable to participate in goal-setting in the GP's professional opinion (e.g. advanced dementia or acute psychosis), had received a care planning consultation in the previous three months, or required translation services to communicate verbally.

Practice administrators searched their electronic patient register according to the eligibility criteria, and a GP then checked the resulting patient list for exclusion criteria. Eligible patients were sent a letter of invitation and participant information leaflet, with the intention of recruiting 10 patients per practice. The number of eligible patients ranged from 47 to 124 and all were invited. The protocol allowed GPs to opportunistically invite patients they thought might be interested, however no patients were recruited through this process. A study researcher visited interested patients at home to discuss the study and obtain written informed consent.

The Norwich Clinical Trials Unit independently randomised three practices to goal-setting and three to control, by simple block randomisation using a 1:1 ratio and sealed opaque envelopes. Practices were randomised after at least 10 expressions of interest were received from patients. It was not possible to blind participants, health professionals or researchers due to the nature of the intervention, with the exception of the statistician undertaking the analysis, who was blinded to the allocation.

Intervention

Both intervention and control practices identified two GPs to either attend the training and deliver goal setting consultations or deliver control consultations, although in one intervention practice (Practice 3) only one GP was able to attend. Therefore five participating GPs from practices allocated to goal-setting (see Table 1) received training in a three hour experiential workshop, led by senior consultation skills tutors (CS and SW) and a GP with experience in communication skills training (AS). One other GP attended the training but withdrew prior to delivering the intervention for personal reasons. The training model we developed for goal setting adapted relevant elements of the work of Elwyn and colleagues on shared decision making [15 16] and of patient-centred care in the leading training model in clinical communication (the Calgary Cambridge Guide [17]). Our model adopted a structured, patient-centred stepped approach. Steps included preparation, goal elicitation, assessing

For peer review only - http://bmjopen.bmj.com/site/about/guidelines.xhtml

options, making goals smart, decision-making and evaluation. Following an introduction to the study, the training was mainly experiential to enable GPs to rehearse existing skills and integrate additional skills for facilitating the goal-setting process. Experiential methods included role-play, video analysis and interactive skill spotting . GPs were trained in groups of three and were given a detailed handbook in advance. The handbook contained information about the study and a "how to" guide for goal-setting, including theoretical background and examples of goal setting. The control group GPs received no training for this study and were asked to undertake a care planning consultation as they would usually do in routine clinical practice. This may have involved a national care planning template, which does not include goal setting, from the Avoiding Unplanned Admissions Enhanced Service [13].

A study researcher discussed goal-setting and the associated paperwork with participants during the face-to-face baseline visit, which lasted approximately 15 minutes. The researcher gave all patient participants a patient-held goal-setting sheet (PGS), with questions to consider prior to their consultation. The questions (Supplementary Appendix 1) were:

- What are your goals? What is important to you? What do you really want to achieve over the next six months?
- Why are these goals important to you?
- What are the first steps you would like to take towards achieving this goal or goals?

The goal-setting consultations were held with the participating GPs even if they were different from the patient's usual GP. During the initial goal-setting consultation GPs, in partnership with participants, documented the goals which had been agreed. GPs then provided support, within their clinical expertise and with the help of other health care professionals, to help patients achieve their goals, for example by providing information on local groups and services. Participants in both the goal-setting and control groups had an initial consultation which lasted about 20 minutes, but only patients in the goal-setting arm were invited back for a follow-up consultation after six months to discuss their goal attainment.

Data and statistical analysis

We collected quantitative and qualitative data to meet the feasibility study objectives. Data collected from patients during a researcher visit at baseline and six months were: health-related quality of life (EQ-5D-5L [18]); capability (as measured through the five attributes of attachment, security, role, enjoyment and control in the ICEpop CAPability measure for Older people questionnaire (ICECAP-O) [19])(ICEPOP is the name of the UK MRC-funded programme through which the index was developed), cognition (general practitioner assessment of cognition scale (GP-COG) [20]) and patient centred care (patient assessment of care for chronic conditions scale (PACIC) [21]). Data collected from the electronic patient record included age, sex and postcode Index of Multiple Deprivation (IMD) score (baseline only), medications on repeat prescription, diagnoses, achievement of relevant quality of care indicators in the Quality and Outcomes Framework [22] and primary and secondary care use (see health economic section below for more details). Practice data were collected before randomisation and patient data were collected after.

GPs and patient participants were asked to complete an assessment of shared decision making during each consultation using the CollaboRATE scale [23] for patients and dyadic OPTION scale [24] for GPs. GPs and patients in the goal-setting group were asked to discuss and complete a goal attainment scaling (GAS-Light) questionnaire [25] (See Supplementary Appendix 2) at the second

For peer review only - http://bmjopen.bmj.com/site/about/guidelines.xhtml

consultation. Goal attainment was scored using the following system: -1 = worse than expected, 0 = no change, 1 = partially attained, 2 = as expected, 3 = a little more and 4 = a lot more than expected.

All initial consultations were video (n=41) or audio (n=4) recorded and transcribed. Three team members scored the consultations using the observer OPTION measure to assess shared decision making [26]. One focus group was held with patients and one with GPs from the goal-setting group at the end of the six month follow-up period to discuss perspectives, experiences and overall acceptability of the goal-setting intervention. All patients in the intervention group were sent a letter of invitation to the focus group, except two who indicated at the researcher visit they did not want to take part. Both focus groups lasted about 90 minutes, were held at the university, guided by a topic guide, audio-recorded and transcribed. Patient or GP participants unable to attend the focus groups were interviewed by phone or face-to-face using the same topic guide.

We calculated the recruitment rate by practice and by randomisation group. Demographic variables were compared for those recruited and those not recruited. The characteristics of baseline consultations were summarised both by practice and by intervention group.

The change in outcome measures from baseline to follow-up was summarised using descriptive statistics by randomisation group. We estimated the difference between randomisation groups using a linear mixed model with practice included as a random effect. This would allow the estimation of potential differences in a full-scale trial. The intra cluster correlation coefficient was estimated for each outcome, however great care should be taken in the interpretation of these due to the small number of clusters [27]. All statistical analyses were undertaken using Stata version 15.

Health economic evaluation

Data were collected on resource use from an NHS perspective to test data collection processes and to inform a future health economic evaluation estimating quality adjusted life years (QALYs). A record was kept of resources required to provide GP training, as well as the length of initial and follow-up goal-setting consultations. Additional health care resource use was extracted from electronic health records by practices supported by a study researcher (EL) for the six-months prior to randomisation and from randomisation to follow-up. Health care use was collected for: day-case and inpatient hospital admissions; outpatient visits; accident and emergency visits (A&E); consultations at the GP practice (GP, practice nurse, health care assistant, nurse practitioners); and other contacts, such as district nursing, allied health professional contacts, ambulance call outs, and specialist nursing contacts.

Resource use was costed using the NHS reference costs [28] for secondary care and a published source for primary care contacts [29]. NHS reference costs were used to estimate a weighted average cost for day cases, non-elective short stay, non-elective long stay, and elective admissions. For longer stays, additional days were costed using a weighted average of all excess bed day costs. For the first and second GP consultations in the goal-setting group, we had data on length of consultation and setting. The cost of providing training was estimated from a description given by the study researcher of duration and required staff. The cost of academic staff time was estimated using University pay scales (including employer's national insurance and superannuation payments). As the training would have relevance beyond the duration of the study, we estimated a useful life of 3 years and calculated an annual equivalent cost [30]. All costs are in 2015/16 UK pounds sterling. As the duration of the study was six-months, we did not discount costs and benefits. As the study size was very small with great variability in estimates of cost and effect, we did not estimate formal cost-effectiveness.

For peer review only

Qualitative analysis

The video and audio recordings of control and goal-setting consultations were compared by the research team (CS, EL, AS, JM and RH) to measure duration and explore the content and methodological implications for a future study. An in-depth analysis of the consultations using a conversation analytic informed approach [31] is reported elsewhere [32].

A thematic framework-based analysis was used to analyse the focus groups recordings and transcripts [33] to assess the acceptability of the goal-setting intervention to patients and GPs and possible future improvements to the goal-setting intervention, training and trial design.

Patient and Public Involvement (PPI)

Four individuals contributed to patient and public involvement (CG, RH, AM, HS). Two PPI representatives contributed to the design of the research as co-applicants on the initial application for funding (AM and HS) and steering group membership (AM and CG). PPI members contributed to the analysis and interpretation of the results, with one PPI representative reviewing and scoring video consultations using OPTION (RH) and a further two reviewing a selection of video consultation transcripts (AM and CG). Two PPI members reviewed and commented on the manuscript and are co-authors (AM and CG).

For peer review only - http://bmjopen.bmj.com/site/about/guidelines.xhtml

Results

Recruitment and retention

Sixty general practices were invited with seven expressing interest and six being recruited (Figure 1). Across the six practices (Table 1), 550 patients met the eligibility criteria and were invited. In total, 52 patients were recruited with 24 belonging to practices randomised to goal-setting and 28 to practices in the control group. Thirteen patients were held in reserve from three practices which had recruited enough patients. The response rate was 12% ((52+13))/550). There was little variation in age, sex and deprivation between those who participated and those who did not (Supplementary Table 1). Two participants in the goal-setting group and five in the control group did not receive the initial consultation because they declined to attend, were unavailable or withdrew consent. Four participants in the goal-setting group did not receive the follow-up consultation because of ill health or death. Data collected directly from participants were available for 18 participants in the goal-setting group and 23 in the control group. Participant data collected from practices were available for 23 participants in the goal-setting group and 28 in the control group. Recruitment of practices took place between December 2016 and February 2017 and recruitment of patients between April and May 2017.

The control practices were in more urbanised areas with larger practice populations and more female GPs participating compared to goal-setting practices (Table 1). The goal-setting group, compared to control (see Table 2), had more patient participants who were female (54% compared to 29%), older (80 years old compared to 77), with a higher number of health problems (5 compared to 4) and medications (13.0 compared to 11.5), but similar quality of life. The control group had participants spread across all four IMD quartiles, whereas the goal-setting group had participants in only the second and third quartiles. All participants were white British and retired, except for one participant in the goal-setting group who was of working age but not employed and one in the control group who was self-employed. There was variation in participant baseline characteristics between practices in mean age (range 69.5 to 85.8 years old), proportion of females (range 25% to 73%), number of medications (range 10.0 to 15.5) and number of health problems (range 3.0 to 7.5) across participating practices.

The mean initial consultation time in the goal-setting group was 23.0 minutes and in the control group was 19.2 minutes (Table 3). GPs in the intervention group saw a mean of 4.4 patients (range 4 to 5), whereas GPs in the control group saw a mean of 3.8 patients (range 2 to 7). Patients spoke more in the goal-setting group initial consultation (mean GP:patient word count ratio (WCR) 1.35) than the control group (WCR 1.52), but this was not statistically significant. Dyadic OPTION scores for GPs perceptions of shared decision making were not statistically significantly higher in the goal-setting group compared to the control group, and collaboRATE scores were similar. Observer OPTION scores showed large variation and inconsistency in scoring between the three research team members (data not presented).

Most patients set two or three goals (Table 4) in the goal setting intervention arm, with GPs and patients setting on average one more goal in Practice 1 than in Practice 3. The commonest types of goals were related to management of chronic conditions, walking, maintaining social and leisure interests and weight management (Table 5). Forty-two of the 50 goals were scored with a mean attainment score per patient of 1.45 (1= partially attained and 2= as expected) with 'partially attained' being the commonest outcome (Table 4).

For peer review only - http://bmjopen.bmj.com/site/about/guidelines.xhtml

In the control arm, goals were rarely mentioned. Four usual-care GPs followed the care planning template recommended within the Avoiding Unplanned Admissions enhanced service [13], one GP appeared to treat it as a normal problem-focused consultation and another GP focused solely on end of life issues.

Outcome measures

As expected in this small feasibility study, there were no statistically significant differences between goal-setting and control from baseline to follow-up in PACIC score, health-related quality of life as measured by EQ5D, number of medications or GPCOG score (Table 6 which also shows the intra-class correlation coefficients). Capability as measured by ICECAP-O at six months, improved slightly more in the control group than in the goal-setting group, but the 95% confidence interval includes zero (mean difference between groups -0.08, 95% CI -0.15 to -0.00).

There was considerable variation in health care use in the six months prior to randomisation and six months follow-up (Table 7). Most health care contact increased in both the control and goal-setting groups, but district nurse contacts increased and inpatient admissions decreased only in the goal-setting group. Quality and Outcomes Framework data were collected at baseline and follow-up, but the results were uninformative due to low numbers and low variability (Supplementary Table 2). There was one death in the goal-setting group due to cancer, which was judged to be unrelated to the intervention. The estimated cost of the goal-setting was £147 per patient, of which £95 related to costs of providing initial and follow-up GP consultations, and £43 related to the cost of GP training. There was a small cost for the study researcher to explain goal-setting. A mean cost of £50 per patient was incurred in the control group for the initial consultation. The single largest cost for the six-months prior to recruitment and the six-months of follow-up was inpatient stays (Table 7). There were also substantial costs in other settings, for example in general practice contacts and district nurse services. The types, number and associated costs of health service use varied considerably, as would be expected in a feasibility study.

Acceptability

Eleven patients expressed interest in the focus group but only six were able to attend on the selected date. Two patients who were unable to attend took part in a telephone interview. Of the five GPs who deliver the intervention, four attended the focus group and one was unable to attend, so was interviewed face-to-face at the GP surgery. All six patient participants attending the focus group reported positive experiences and views of the intervention, particularly regarding the different emphasis of the consultation. Participants spoke of goal setting providing clarity about what mattered to them, and helping them to plan and focus their lives

> *"[Goal-setting] gives he or she a much better understanding of particularly what is worrying you, what your aims are, the things that you miss being able to do and to be able to actually explain it where [GPs] have time, because very often the GPs, you know, you've only got ten minutes. But with these consultations, you're actually able to talk to a doctor, as you would indeed a friend almost" (Patient 107)*

Goal-setting appeared to function as a mechanism for helping make consultations patient-centred. This was reflected in the unanimous support for the intervention amongst the four GPs who attended the GP focus group and one GP who was interviewed by phone. GPs described the goal-setting consultations as *more patient-centred and reflected on its 'therapeutic powers'* (GP10) compared to day-to-day general practice, which GPs felt could be dominated by '*box-ticking*' and '*target driven*' (GP018) medicine.

For peer review only - http://bmjopen.bmj.com/site/about/guidelines.xhtml

"I felt almost as if I was trying to put on a different hat, you know, trying not to constantly interrupt them or to sort of sway them in any way, I was trying to give them the opportunity to just say what they wanted to say and set any goal that they wanted to and I, and it made me reflect on actually what I do during the day to day when I've got ten minutes with a patient and I'm very aware of the sort of pressure of, oh I've got to do a medication review and I've got to do this and oh no, their cholesterol's now 7 and oh gosh I've, have my colleagues already spoke to them about this and are they aware of X, Y and Z and actually it was quite nice in a way just take a step back and think, um I don't have to do that with this consultation, let's see what happens when the patient has more control over it" (GP025)

Patient participants spoke positively about the baseline researcher visit because it helped them understand the study and encouraged them to reflect on what was important. However, when discussing wider implementation across the health service, participants acknowledged that a home visit for each patient may be too costly and alternative provision would be acceptable to most people. Patients were reluctant to receive more paperwork as they felt that it was a burden for some people. When asked by the moderator to consider the acceptability of a group session to introduce people to the study and to the concept of goal-setting, all bar one of the patient participants at the focus group felt this would be acceptable.

Continuity of care was a concern for patient participants. While one person was disappointed not to see their own GP, three were positive about consulting with a different doctor, especially if it was difficult to see their usual GP. However, participants spoke of wanting more follow-up and consistency amongst the health care team in relation to their goals in the future; some participants felt there was a disconnection between the activity of goal setting and their subsequent treatment by staff within the practice.

GPs stated that the experiential work, especially role play and skill spotting, was the most useful aspect of training. When discussing delivering training at scale, GPs felt e-training with opportunities to watch '*other people role-play*', would fit in with their busy schedules. In addition, multiple shorter e-training modules, using a '*step-by-step*' approach (GP014) that contributed to continuing professional development, would be attractive to GPs when implementing the intervention more widely.

For peer review only

Discussion

The process of setting goals in a GP consultation and follow-up over six months was acceptable to patients and unanimously supported by participating GPs. Recruitment and retention of practices and patients was achieved. A wide range of goals were set and, as expected with a feasibility study, there were no statistically significant differences in the main outcomes. Goal setting consultations were a similar length to control consultations. The qualitative findings were that goal-setting helped patients and GPs focus on what was important and supported GPs to deliver more patient-centred care. Patient preparedness, continuity of care and being able to deliver training at scale were important considerations for future studies of goal setting. Data on the number of health problems were not sufficiently robust for analysis because they were extracted from practice records using different processes. Asking GPs in the non-intervention group to undertake a video-recorded usual care planning consultation is likely to have altered practice compared to what would have happened within the enhanced service. An intention-to-treat analysis was undertaken to reduce the impact of protocol violations (e.g. patients not receiving the pre-specified intervention).

A Cochrane review, published in 2015, assessed the effects of personalised care planning (defined as goal-setting and action planning), for adults with long term health conditions compared to usual care [8]. Whilst 19 RCTs were included, all except for one focused on single conditions. The one multiple condition study included patients who had high health care use and focused on care planning, with goal-setting as part of the process, across the wider health care system to reduce unplanned admissions [34]. The authors found an increase in quality of life (measured by SF36) in the intervention compared to control, however with 50% of participants lost to follow-up and intention to treat not undertaken, there is a possibility of a lost to follow-up bias in favour of the intervention. Our study has focused on goal-setting specifically in primary care.

A systematic review of randomised and non-randomised studies, published in 2017, looked at collaborative goal-setting or health priority setting for elderly people with a chronic condition or multimorbidity [12]. The authors found that in four of eight intervention studies, multifactorial approaches improved goal-setting or care planning, but the review did not assess health outcomes or quality of life. The authors concluded that future research was needed to determine the "mix of essential elements within a multifactorial intervention to provide recommendations on daily practice". Our study helps to answer this question by identifying some key requirements of goal-setting in primary care.

This was a feasibility study and the main implications are for the design of a subsequent definitive trial. Our objectives were to assess participant recruitment and retention, the acceptability of a goal-setting intervention to patients and GPs, the training needs of GPs, the content of control consultations, goal-setting and the feasibility of collecting relevant outcome measures.

We set out to recruit six practices, and seven (out of 60 invited) were willing to take part after one initial email invitation. Participant recruitment and retention was sufficient overall, but low in one practice (which recruited four out of a target of ten). Reminder letters were not sent, but these may help all practices to recruit larger numbers if required in a future study. Seven participants, five from the control and two from goal-setting, did not receive the initial consultation because they declined the consultation, withdrew consent or were not able to attend. Possibly some were disappointed to be allocated to the control group.

Goal-setting was acceptable to participating patients and GPs, albeit a self-selecting group who were willing to take part in research into goal-setting. Goal setting is unlikely to be relevant to everyone,

For peer review only - http://bmjopen.bmj.com/site/about/guidelines.xhtml

but the positive response of participants in this feasibility study suggests that it is likely to have wider acceptability in general practice. Further research is needed to understand which patients will benefit most from goal setting. The readiness of patients to undertake goal-setting appeared to be important. Although several goals were only partially attained, GPs and patients still felt them to be worthwhile, suggesting that the process of goal setting has benefits, apart from the achievement of goals.

Training participating GPs in goal-setting was important, and participating GPs thought that the face-to-face training with role play used in the feasibility study could be replaced with online e-learning to allow delivery at scale to a wider GP workforce. The initial researcher visit was important to participants and the key elements of this visit would be delivered in a future trial using video and leaflet-based patient information aids, again to be developed using material collected during this feasibility study.

Goal setting consultations were more focussed on what matters to the patient than the control consultations. Key challenges in goal setting included preparation and agreeing goals and we explore these further elsewhere [32]. Some patients were concerned that their goals were not considered in future consultations, which suggests that better communication of goals with the rest of the health care team will be needed. Planned follow-up of goals with the GP sooner than six months if needed would improve continuity of care, which is associated with lower mortality [35].

We collected a wide range of outcome measures in order to assess their feasibility and suitability for use in a future trial. Both EQ-5D-5L and the ICECAP-O should be used in a future economic evaluation but would not be the best primary outcome measure for a trial of goal setting. A recent study which aimed to improve the management of patients with multimorbidity, the 3D study, used the EQ5D5L as a primary outcome, but did not find any significant difference between arms [36]. It may be that the domains within the EQ5D5L are insensitive to changes in care for patients with multimorbidity and a measure of patient centred care such as PACIC is a more appropriate primary outcome measure as it contains a sub scale to measure goal setting. Baseline and follow-up data were collected during researcher visits, which could be replaced by postal questionnaires as the amount and complexity of data to be collected would be reduced. Postal questionnaires are widely used in research and could either increase or reduce the completeness of follow-up data, depending on the preference of individuals for a visit rather than a postal form to complete.

Quality and Outcomes Framework data did not prove useful because of the small numbers and low variation. The observer OPTION scoring, initially developed within a rehabilitation context, had low consistency between researchers and therefore was not useful. A possible reason for this lack of consistency was that OPTION was developed for specific clinical decisions, and not for goal setting which often involved multiple complex decisions.

Goal-setting can be valuable for GPs and patients seeking to agree the desired outcomes of care, particularly for older patients with multimorbidity. This study has demonstrated that it is acceptable and feasible in general practice, and a full trial is now needed to assess whether goal setting improves important clinical outcomes for patients.

For peer review only

For peer review only - http://bmjopen.bmj.com/site/about/guidelines.xhtml

Competing interests: None declared.

Author contributions: NS, JF, CS and AS conceived the idea. NS, JF, EL, CS, DT, AS, AC, JM, CG, SJ, IK, AL, AM, TW and SW contributed to the design of the study. EL led the data collection. CS, JM and AS led the analysis of the qualitative data. AC undertook the statistical analysis. DT undertook the economic analysis. NS, JF, EL, CS, DT, AS, AC, JM, CG, SJ, IK, AL, AM, TW and SW contributed to the interpretation of the results. JF drafted the initial manuscript. NS, JF, EL, CS, DT, AS, AC, JM, CG, SJ, IK, AL, AM, TW and SW revised the manuscript and approved the final version. NS is the guarantor.

Acknowledgements: We thank Rebecca Harmston (RH) for reviewing the video-consultations from the patient and public involvement perspective, Clara Yates and Gosia Majsak-Newman at the Norfolk and Suffolk Primary and Community Care Research Office for their support, and the patients and staff who took part in the study.

Funding statement: This paper presents independent research supported by the National Institute for Health Research (NIHR) under its Research for Patient Benefit (RfPB) Programme (Grant Reference Number PB-PG-0215-36079) and by the NIHR Collaboration for Leadership in Applied Health Research and Care East of England Programme. The views expressed are those of the author(s) and not necessarily those of the NHS, the NIHR or the Department of Health and Social Care. The funders did not have any role in the design, collection, analysis or interpretation of data or in writing the manuscript.

Data sharing:

Dataset of quantitative data and statistical code is available from the corresponding author.

For peer review only

References

1. Steel N, Ford JA, Newton JN, et al. Changes in health in the countries of the UK and 150 English Local Authority areas 1990 to 2016: a systematic analysis for the Global Burden of Disease Study 2016. *Lancet* 2018;**392**:1647-61 doi: 10.1016/S0140-6736(18)32207-4.

2. Barnett K, Mercer SW, Norbury M, Watt G, Wyke S, Guthrie B. Epidemiology of multimorbidity and implications for health care, research, and medical education: a cross-sectional study. *Lancet* 2012;**380**:37-43 doi: 10.1016/s0140-6736(12)60240-2.

3. Fortin M, Lapointe L, Hudon C, Vanasse A, Ntetu AL, Maltais D. Multimorbidity and quality of life in primary care: a systematic review. *Health and Quality of Life Outcomes* 2004;**2**:51-51 doi: 10.1186/1477-7525-2-51.

4. Wilkie P. Really putting patients first: ensuring significant involvement for patients in healthcare decision making. *Br J Gen Pract* 2015;**65**:108-09 doi: 10.3399/bjgp15X683821.

5. Reuben DB, Tinetti ME. Goal-Oriented Patient Care — An Alternative Health Outcomes Paradigm. *NEMJ* 2012;**366**:777-79 doi: 10.1056/NEJMp1113631.

6. Guthrie B, Payne K, Alderson P, McMurdo MET, Mercer SW. Adapting clinical guidelines to take account of multimorbidity. *BMJ* 2012;**345** doi: 10.1136/bmj.e6341.

7. National Institute for Health and Care Excellence. Multimorbidity: clinical assessment and management NICE guideline [NG56]. London, 2016. https://www.nice.org.uk/guidance/ng56 Accessed on 8 Nov 2018.

8. Coulter A, Entwistle VA, Eccles A, Ryan S, Shepperd S, Perera R. Personalised care planning for adults with chronic or long-term health conditions. *Cochrane Database Syst Rev* 2015:Cd010523 doi: 10.1002/14651858.CD010523.pub2.

9. Reeves D, Hann M, Rick J, et al. Care plans and care planning in the management of long-term conditions in the UK: a controlled prospective cohort study. *Br J Gen Pract* 2014;**64**:e568-75 doi: 10.3399/bjgp14X681385.

10. Burt J, Roland M, Paddison C, et al. Prevalence and benefits of care plans and care planning for people with long-term conditions in England. *J Health Serv Res Policy* 2012;**17 Suppl 1**:64-71 doi: 10.1258/jhsrp.2011.010172.

11. Elwyn G, Laitner S, Coulter A, Walker E, Watson P, Thomson R. Implementing shared decision making in the NHS. *BMJ* 2010;**341**:c5146 doi: 10.1136/bmj.c5146.

12. Vermunt NPCA, Harmsen M, Westert GP, Olde Rikkert MGM, Faber MJ. Collaborative goal setting with elderly patients with chronic disease or multimorbidity: a systematic review. *BMC Geriatr* 2017;**17**:167 doi: 10.1186/s12877-017-0534-0.

13. NHS England. Enhanced service specification: Avoiding unplanned admissions: proactive case finding and patient review for vulnerable people. 2014. https://www.england.nhs.uk/wp-content/uploads/2014/06/avoid-unpln-admss-serv-spec.pdf Accessed on 8 nov 2018.

14. Steel N, Clark A, Ford J, et al. Goal-setting in care planning for people with multimorbidity: feasibility study and intervention refinement Norwich: University of East Anglia, 2016. http://www.uea.ac.uk/medicine/research/primary-care-and-epidemiology/goalplan Accessed on 8 Nov 2018.

15. Elwyn G, Frosch D, Thomson R, et al. Shared Decision Making: A Model for Clinical Practice. *J Gen Intern Med* 2012;**27**:1361-67 doi: 10.1007/s11606-012-2077-6.

16. Elwyn G, Durand MA, Song J, et al. A three-talk model for shared decision making: multistage consultation process. *BMJ* 2017;**359** doi: 10.1136/bmj.j4891.

17. Silverman J, Kurtz S, Draper J. *Skills for communicating with patients*: CRC Press, 2016.

18. Herdman M, Gudex C, Lloyd A, et al. Development and preliminary testing of the new five-level version of EQ-5D (EQ-5D-5L). *Qual Life Res* 2011;**20**:1727-36 doi: 10.1007/s11136-011-9903-x.

19. Coast J, Flynn TN, Natarajan L, et al. Valuing the ICECAP capability index for older people. *Soc Sci Med* 2008;**67**:874-82 doi: 10.1016/j.socscimed.2008.05.015.

For peer review only

For peer review only - http://bmjopen.bmj.com/site/about/guidelines.xhtml

20. Brodaty H, Pond D, Kemp NM, et al. The GPCOG: a new screening test for dementia designed for general practice. *J Am Geriatr Soc* 2002;**50**:530-4.
21. Glasgow RE, Wagner EH, Schaefer J, Mahoney LD, Reid RJ, Greene SM. Development and validation of the Patient Assessment of Chronic Illness Care (PACIC). *Med Care* 2005;**43**:436-44.
22. NHS Employers. General Medical Services (GMS) contract Quality and Outcomes Framework (QOF). 2014. https://www.england.nhs.uk/gp/gpfv/investment/gp-contract/ Accessed on 8 Nov 2018.
23. Barr PJ, Thompson R, Walsh T, Grande SW, Ozanne EM, Elwyn G. The psychometric properties of CollaboRATE: a fast and frugal patient-reported measure of the shared decision-making process. *J Med Internet Res* 2014;**16**:e2 doi: 10.2196/jmir.3085.
24. Melbourne E, Sinclair K, Durand MA, Legare F, Elwyn G. Developing a dyadic OPTION scale to measure perceptions of shared decision making. *Patient Educ Couns* 2010;**78**:177-83 doi: 10.1016/j.pec.2009.07.009.
25. Turner-Stokes L. Goal attainment scaling (GAS) in rehabilitation: a practical guide. *Clin Rehabil* 2009;**23**:362-70 doi: 10.1177/0269215508101742.
26. Barr PJ, O'Malley AJ, Tsulukidze M, Gionfriddo MR, Montori V, Elwyn G. The psychometric properties of Observer OPTION5, an observer measure of shared decision making. *Patient Educ Couns* 2015;**98**:970-76 doi: https://doi.org/10.1016/j.pec.2015.04.010.
27. Eldridge SM, Costelloe CE, Kahan BC, Lancaster GA, Kerry SM. How big should the pilot study for my cluster randomised trial be? *Stat Methods Med Res* 2016;**25**:1039-56 doi: 10.1177/0962280215588242.
28. Department of Health and Social Care. NHS reference costs 2015 to 2016. London, 2016. https://www.gov.uk/government/publications/nhs-reference-costs-2015-to-2016 Accessed on 8 Nov 2018.
29. Curtis L. Unit Costs of Health and Social Care 2017. Canterbury: University of Kent 2017. https://www.pssru.ac.uk/project-pages/unit-costs/unit-costs-2017/ Accessed on 8 Nov 2018.
30. Drummond MF, Sculpher MJ, Claxton K, Stoddart GL, Torrance GW. *Methods for the economic evaluation of health care programmes*: Oxford university press, 2015.
31. Drew P, Chatwin J, Collins S. Conversation analysis: a method for research into interactions between patients and health-care professionals. *Health Expect* 2001;**4**:58-70.
32. Salter C, Shiner A, Lenaghan E, et al. Setting goals with patients living with multimorbidity: qualitative analysis of general practice consultations. *Br J Gen Pract* 2018:undergoing peer review.
33. Ritchie J, Lewis J, Nicholls CM, Ormston R. *Qualitative research practice: A guide for social science students and researchers*: Sage, 2013.
34. Battersby M, Harvey P, Mills PD, et al. SA HealthPlus: a controlled trial of a statewide application of a generic model of chronic illness care. *Milbank Q* 2007;**85**:37-67 doi: 10.1111/j.1468-0009.2007.00476.x.
35. Pereira Gray DJ, Sidaway-Lee K, White E, Thorne A, Evans PH. Continuity of care with doctors—a matter of life and death? A systematic review of continuity of care and mortality. *BMJ Open* 2018;**8** doi: 10.1136/bmjopen-2017-021161.
36. Salisbury C, Man MS, Bower P, et al. Management of multimorbidity using a patient-centred care model: a pragmatic cluster-randomised trial of the 3D approach. *Lancet* 2018;**392**:41-50 doi: 10.1016/s0140-6736(18)31308-4.
37. Office for National Statistics. 2011 Rural/urban Classification. London, 2011. http://www.ons.gov.uk/ons/guide-method/geography/products/area-classifications/2011-rural-urban/index.html Accessed on 8 Nov 2018.

For peer review only

For peer review only - http://bmjopen.bmj.com/site/about/guidelines.xhtml

602 Figure legends

603 Figure 1: Consort flow diagram

For peer review only

For peer review only - http://bmjopen.bmj.com/site/about/guidelines.xhtml

Table 1: Baseline characteristics of participating practices and patients, by practice

	Goal-setting			Control		
	**Practice 1**	**Practice 2**	**Practice 3**	**Practice 4**	**Practice 5**	**Practice 6**
**Practice characteristics**						
Practice rurality*	Village	Town and fringe	Town and fringe	Urban >10K	Urban >10K	Urban >10K
Patient population	5000 to 9,900	10,000 to 14,900	5000 to 9,900	>14,900	10,000 to 14,900	10,000 to 14,900
IMD practice decile	7	5	7	9	5	5
Characteristics of participating GPs	n=2 both male, partners and working part-time	n=2 one male, one female, both partners and working full-time	n=1 male, partner working part-time	n=2 One male, one female, both partners, one working full-time and one part-time	n=2 both female, partners and working part-time	n=2 both female, partners and working part-time
Years qualified of participating GPs	GP014 = >20 yrs; GP018 = 10 to 20 yrs	GP025 = <10 yrs; GP026 = 10 to 20 yrs	GP038 = 10 to 20 yrs	GP046 = >20 yrs; GP047 = >20 yrs	GP053 = >20 yrs; GP055 = >20 yrs	GP061 = 10 to 20 yrs; GP067 = 10 to 20 yrs
**Practice recruitment**						
Patients assessed for eligibility, n	9067	14845	6791	18540	10381	13439
Patients invited, n (% assessed)	77 (0.8)	108 (0.7)	47 (0.7)	108 (0.6)	124 (1.2)	86 (0.6)
Recruited, n (% invited)*	11 (14.3)	9 (8.3)	4 (8.5)	8 (7.4)	10 (11.6)	10 (11.6)

*ONS indicator 2011 [37], ** = based on Barnett list [2] IMD = Index of Multiple Deprivation (1= most deprived and 10 least deprived), partner = GP with responsibility for the practice, n= number, SD = standard deviation, IQR = Interquartile Range, n= number, *=does not include those on the reserve list (see Figure 1)

For peer review only - http://bmjopen.bmj.com/site/about/guidelines.xhtml

Table 2: Baseline characteristics of patient participants

Variable		Control	Goal-setting
Number		28	24
Female n (%)		11 (39%)	13 (54%)
Age mean (SD)		77.18 (9.42)	80.42 (8.72)
GPCOG category n (%)	Impairment and further investigations implied	1 (4%)	0 (0%)
	Informant interview required	17 (61%)	19 (79%)
	No cognitive impairment	10 (36%)	5 (21%)
Number of diagnoses* median (IQR)		4.00 (3.00, 5.00)	5.00 (3.00, 6.00)
IMD national quartile n (%)	1	5 (18%)	0 (0%)
	2	9 (32%)	14 (58%)
	3	3 (11%)	10 (42%)
	4	11 (39%)	0 (0%)
Marital status n (%)	Divorced	0 (0%)	2 (8%)
	Living with partner	0 (0%)	2 (8%)
	Married	12 (43%)	10 (42%)
	Single	2 (7%)	4 (17%)
	Widowed	14 (50%)	6 (25%)

N= number, SD = standard deviation, IQR = Interquartile Range, GPCOG = General Practitioner assessment of Cognition, PACIC = Patients Assessment Chronic Illness Care, EQ-5Q-5L = 5 level EQ-5D, ICECAP-O = ICEpop CAPability measure for Older people, * = based on Barnett list [2], IMD = Index of Multiple Deprivation

For peer review only

Table 3: Characteristics of initial consultations

	Intervention group				Control group				Mean difference between intervention and control (95% CI)
	Practice 1 (n = 10)	Practice 2 (n = 8)	Practice 3 (n = 4)	Intervention total (n = 22)	Practice 4 (n = 7)	Practice 5 (n = 9)	Practice 6 (n = 7)	Control total (n = 23)	
Duration of initial consultation (mins) mean (SD)	24.1 (4.0)	23.3 (4.4)	19.9 (6.2)	23.0 (4.6)	14.3 (4.8)	25.2 (5.7)	16.3 (4.1)	19.2 (6.9)	3.88 (-3.25,11.01)
Dyadic OPTION scores mean (SD)	65.3 (9.0)	63.2 (6.4)	62.5 (3.6)	64.0 (7.2)	63.5 (13.0)	62.7 (4.0)	42.1 (20.4)	56.6 (16.2)	7.57 (-6.37,21.50)
CollaboRATE scores mean (SD)	7.8 (1.0)	8.5 (0.9)	8.8 (0.2)	8.2 (1.0)	7.0 (2.6)	8.6 (0.7)	8.7 (0.6)	8.1 (1.8)	0.20 (-1.06,1.47)
GP:patient word count ratio mean (SD)	1.23 (0.40)	1.41 (0.78)	1.50 (1.05)	1.35 (0.67)	1.13 (0.45)	1.92 (0.75)	1.39 (0.52)	1.52 (0.67)	-0.14 (-0.65,0.37)

SD= standard deviation, n= number, 95%CI = 95% confidence interval

For peer review only - http://bmjopen.bmj.com/site/about/guidelines.xhtml

Table 4: Patient participants, goals set and attainment scores by practice

		Practice 1	Practice 2	Practice 3	Overall
Number of patients		10	8	4	22
Number of patients setting 1, 2 or 3 goals	1 goal	0	2	1	3
	2 goals	3	4	3	10
	3 goals	7	2	0	9
Number of goals set		27	16	7	50
Number of goals with data available for attainment scoring		21	15	6	42
Number of goals in each attainment score category (category score) n (%)	worse than expected (-1)	1 (4.8)	2 (13.3)	1 (16.7)	4 (9.5)
	no change (0)	4 (19.0)	0 (0.0)	2 (33.3)	6 (14.3)
	partially attained (1)	9 (42.9)	5 (33.3)	1 (16.7)	15 (35.7)
	as expected (2)	2 (9.5)	3 (20.0)	1 (16.7)	6 (14.3)
	a little more (3)	2 (9.5)	4 (26.7)	0 (0.0)	6 (14.3)
	a lot more than expected (4)	3 (14.3)	1 (6.7)	1 (16.7)	5 (11.9)
Mean goal attainment score per patient (range -1 to 4)		1.43	1.67	1.0	1.45

For peer review only

For peer review only - http://bmjopen.bmj.com/site/about/guidelines.xhtml

Table 5: Categories of goals set

Goal categories	Number of goals
Management of chronic condition (non-medication)	9
Walking-related	8
Maintain interests	5
Management of chronic condition (medication-related)	5
Gain weight	4
Social participation	3
Healthy living	3
Balance/mobility	3
Gardening-related	3
Manual dexterity	3
Mental health	2
End of life management	1
Cooking/food preparation	1
Grand Total	50

For peer review only

For peer review only - http://bmjopen.bmj.com/site/about/guidelines.xhtml

Table 6: Change in outcome measures between groups at six months

Variable	Control				Intervention				Mean difference-in-difference between goal-setting and control (95% CI)	Intraclass correlation coefficient (95% CI)
	n	Baseline, mean (SD)	Follow-up, mean (SD)	Difference, mean (SD)	n	Baseline, mean (SD)	Follow-up, mean (SD)	Difference, mean (SD)		
Number of medication	28	12.5 (8.19)	12.79 (7.25)	0.29 (2.65)	23	13.61 (4.56)	14.65 (4.44)	1.04 (3.21)	0.76 (-0.85,2.37)	0.00*
GPCOG	23	7.35 (1.70)	6.78 (2.19)	-0.57 (2.02)	19	7.58 (1.30)	7.00 (2.26)	-0.58 (2.29)	0.09 (-1.65,1.84)	0.08 (0.00,0.77)
PACIC	23	1.45 (0.30)	1.85 (0.77)	0.40 (0.69)	18	1.94 (0.76)	2.25 (0.70)	0.31 (0.98)	-0.09 (-0.60,0.42)	0.00*
EQ-5D-5L	23	0.54 (0.34)	0.52 (0.35)	-0.02 (0.19)	18	0.56 (0.25)	0.55 (0.28)	-0.01 (0.15)	0.02 (-0.11,0.13)	0.05 (0.00,0.94)
ICECAP-O	22	0.72 (0.26)	0.78 (0.20)	0.06 (0.14)	17	0.78 (0.12)	0.77 (0.13)	-0.02 (0.06)	-0.08 (-0.15,-0.00)	0.00*

SD = standard deviation, GPCOG = General Practitioner assessment of Cognition, PACIC = Patients Assessment Chronic Illness Care, EQ-5Q-5L = 5 level EQ-5D, ICECAP-O = ICEpop CAPability measure for Older people, 95%CI = 95% confidence interval

*The confidence interval was not reported in cases when the ICC is zero as the standard error is undefined in these cases

For peer review only

Table 7: Costs associated with health care use in

Resource use	6-months prior to recruitment						Recruitment to 6-month follow-up					
	Control			Goal-setting			Control			Goal-setting		
	Total contacts n	Total cost £	Mean cost £ (SD)	Total contacts n	Total cost £	Mean cost £ (SD)	Total contacts n	Total cost £	Mean cost £ (SD)	Total contacts n	Total cost £	Mean cost £ (SD)
Community based services												
GP	157	4,636	166 (164)	89	2,464	107 (115)	177	5,150	184 (150)	124	4,002	174 (145)
Other practice based	97	922	33 (42)	108	1,080	47 (30)	152	1,823	65 (58)	149	1,529	66 (53)
District Nurse	148	3,582	128 (546)	198	6,450	280 (1297)	100	2,879	103 (321)	241	7,450	324 (1384)
Other	72	1,434	51 (132)	72	2,601	113 (193)	189	7,652	273 (355)	97	5,510	240 (224)
All community based	474	10,575	378 (778)	467	12,594	548 (1520)	618	15,681	560 (719)	611	16,962	737 (1537)
Inpatient	4	11,291	403 (1113)	16	28,054	1220 (2584)	12	35,055	1252 (2203)	13	39,889	1734 (4815)
Outpatient	45	4,848	173 (208)	51	7,381	321 (397)	41	4,424	158 (202)	52	6,295	274 (329)
A&E	1	138	5 (26)	6	826	36 (74)	15	2,066	74 (109)	16	2,204	96 (128)
Total for all costs		26,853	959 (1776)		48,856	2124 (4031)		57,226	2044 (2665)		65,349	2841 (4968)

SD = standard deviation, A&E = Accident and Emergency

For peer review only - http://bmjopen.bmj.com/site/about/guidelines.xhtml

For peer review only

For peer review only - http://bmjopen.bmj.com/site/about/guidelines.xhtml

Figure 1: Consort flow diagram

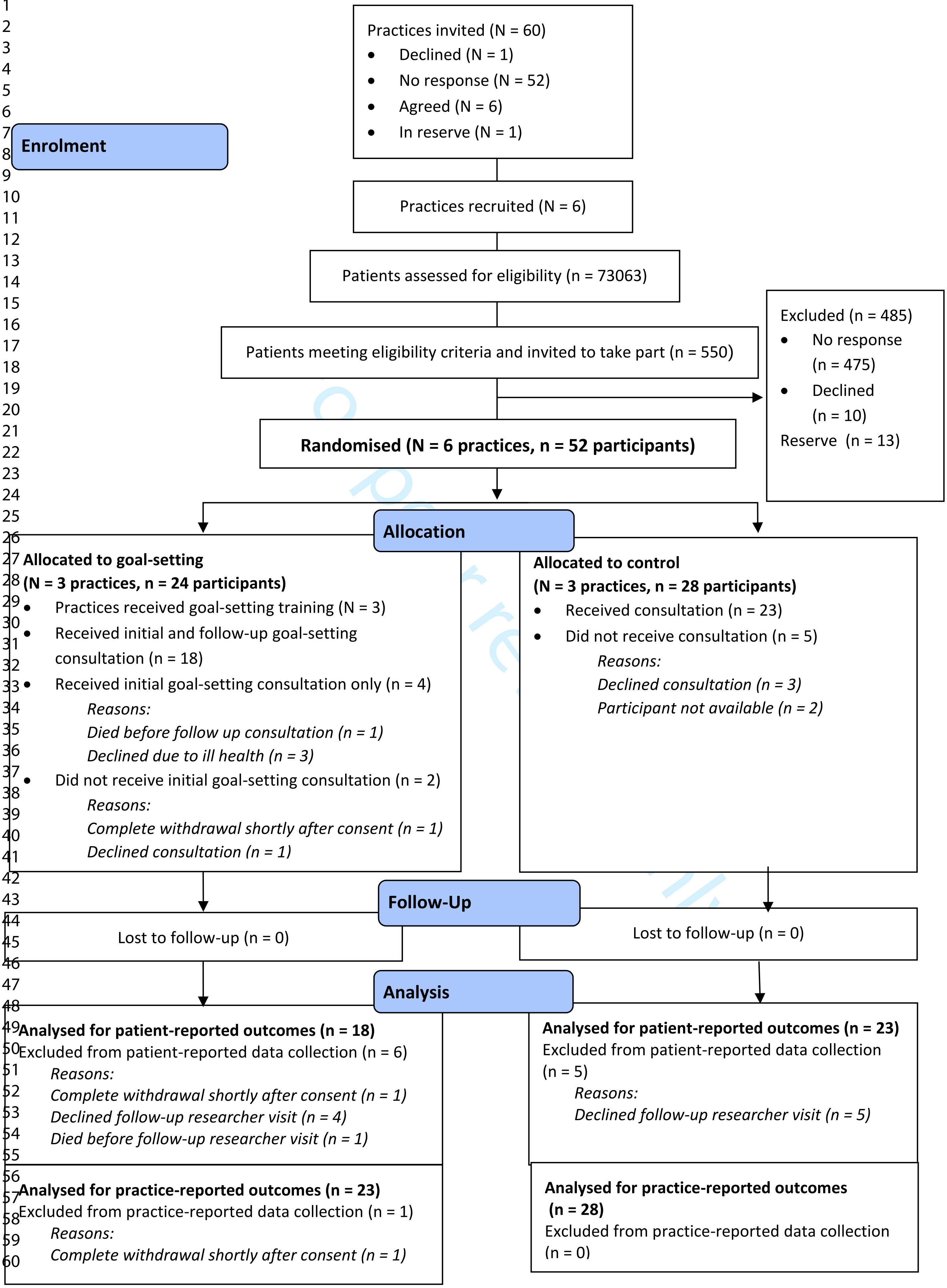

(N= number of practice, n= number of individuals)

Supplementary Table 1: Characteristics of those who participated compared with those who did not

	Participation	Non-participation
Number	52	498
Age mean (SD)	78.5 (9.0)	79.6 (12.2)
Female %	46.2%	53.8%
IMD decile mean (SD)	5.8 (2.3)	5.3 (2.2)

SD = standard deviation, IMD = Index of Multiple Deprivation

For peer review only

Supplementary Table 2: Quality and Outcomes Framework data

		Goal-setting	Control
BMI	No of participants	2	3
	Baseline mean (SD)	28.4 (1.9)	37.8 (8.0)
	Follow-up (mean, SD)	28.5 (3.6)	37.0 (9.5)
	Diff (mean, SD)	0.1 (1.7)	-0.8 (2.7)
BP, mmHg	No of participants	5	5
	Baseline systolic (mean, SD)	133.5 (6.5)	127.5 (19.0)
	Baseline diastolic (mean, SD)	70.7 (4.4)	69.2 (5.5)
	Follow-up systolic (mean, SD)	144.7 (7.0)	124.4 (6.2)
	Follow-up diastolic (mean, SD)	80.6 (4.7)	67.1 (5.6)
	Mean diff systolic (mean, SD)	11.2 (12.6)	-3.1 (14.4)
	Mean diff diastolic (mean, SD)	9.9 (3.1)	-2.1 (8.7)
	Baseline Qof target met (150/90)	5/5	5/5
	Follow-up Qof target met (150/90)	4/5	5/5
eGRFR, mL/min/1,73m^2	No of participants	4	6
	Baseline (mean, SD)	54 (14)	57 (24)
	Follow-up (mean, SD)	56 (17)	59 (25)
	Mean diff (mean, SD)	2 (5)	2 (3)
HB1Ac, mmol/mol	No of participants	1	3
	Baseline (mean, SD)	80 (NA)	39 (3)
	Follow-up (mean, SD)	87 (NA)	43 (6)
	Mean diff (mean, SD)	7 (NA)	4 (3)
	Baseline Qof target met - Diabetes and HB1Ac <59	0/1	1/1
	Baseline Qof target met - Diabetes and HB1Ac <64	0/1	1/1
	Baseline Qof target met - Diabetes and HB1Ac <75	0/1	1/1
	Follow-up Qof target met - Diabetes and HB1Ac <59	0/1	1/1
	Follow-up Qof target met - Diabetes and HB1Ac <64	0/1	1/1
	Follow-up Qof target met - Diabetes and HB1Ac <75	0/1	1/1
Total cholesterol, mg/dL	No of participants	2	1
	Baseline (mean, SD)	2.8 (0.7)	4.2 (NA)
	Follow-up (mean, SD)	3.9 (1.1)	4.9 (NA)
	Mean diff (mean, SD)	1.1 (0.5)	0.7 (NA)
HDL cholesterol, mg/dL	No of participants	2	0
	Baseline (mean, SD)	0.84 (0.19)	NA
	Follow-up (mean, SD)	1.01 (0.4)	NA
	Mean diff (mean, SD)	0.17 (0.15)	NA

BMI = body mass index, SD= standard deviation, BP = blood pressure, eGFR = estimated glomerular filtration rate, HB1Ac = glycated haemoglobin, Qof = Quality and Outcomes Framework, HDL = High Density Lipoproteins, NA= not applicable

For peer review only

GoalPlan Study:
Goal-setting form
for completion before care plan appointment

Part 1) What are your goals? What is important to you?

Write down what you would really like to do or achieve over the next 6 months, even if you think it may not be related to your health. Think about things that you would like to do in your personal, home, work, and social life—things that you ***need*** to do, ***want*** to do and / or ***enjoy doing***. Then list them in order of priority— starting with 1 for the goal that matters to you most and that you would like to focus on at the moment. To help you, we have put a completed example overleaf.

What do you really want to achieve over the next 6 months? (your goals)

For peer review only

Example

- *I want to be able to do more in the garden as it relaxes me*
- *I want to lose weight so I can walk more confidently at my daughter's wedding and fit into my favourite outfit again*
- *I want to be able to get more exercise as it used to help me sleep well*
- *I want to get back to driving so I can visit my friend at his house every week*
- *I want to be able to go out with friends once a week again for lunch*

Part 2) Why are these goals important to you?

We now ask you to focus on your goals from Part 1, and to think about why they are important to you. Below is an example of how to find out if a goal you have come up with is important for its own sake, or if it is important because it will help you achieve something else. We ask you to do this exercise with up to three of your goals overleaf.

Part 3) What are the first steps you would like to take towards achieving this goal or goals?

Having identified your most important goals, the final step on this form is to start thing about steps you would like to take to achieve those goals. This is also included in the example below. Please do the same for your goals overleaf.

EXAMPLE GOAL 1:
I want to be able to do more in the garden

Why are these goals important to you?

Gardening is my hobby and gives me pleasure. It is my main form of exercise.

What first steps can I take towards achieving this goal?

Discuss changes to medicines with GP – might reducing medicines help me be more active?

For peer review only - http://bmjopen.bmj.com/site/about/guidelines.xhtml

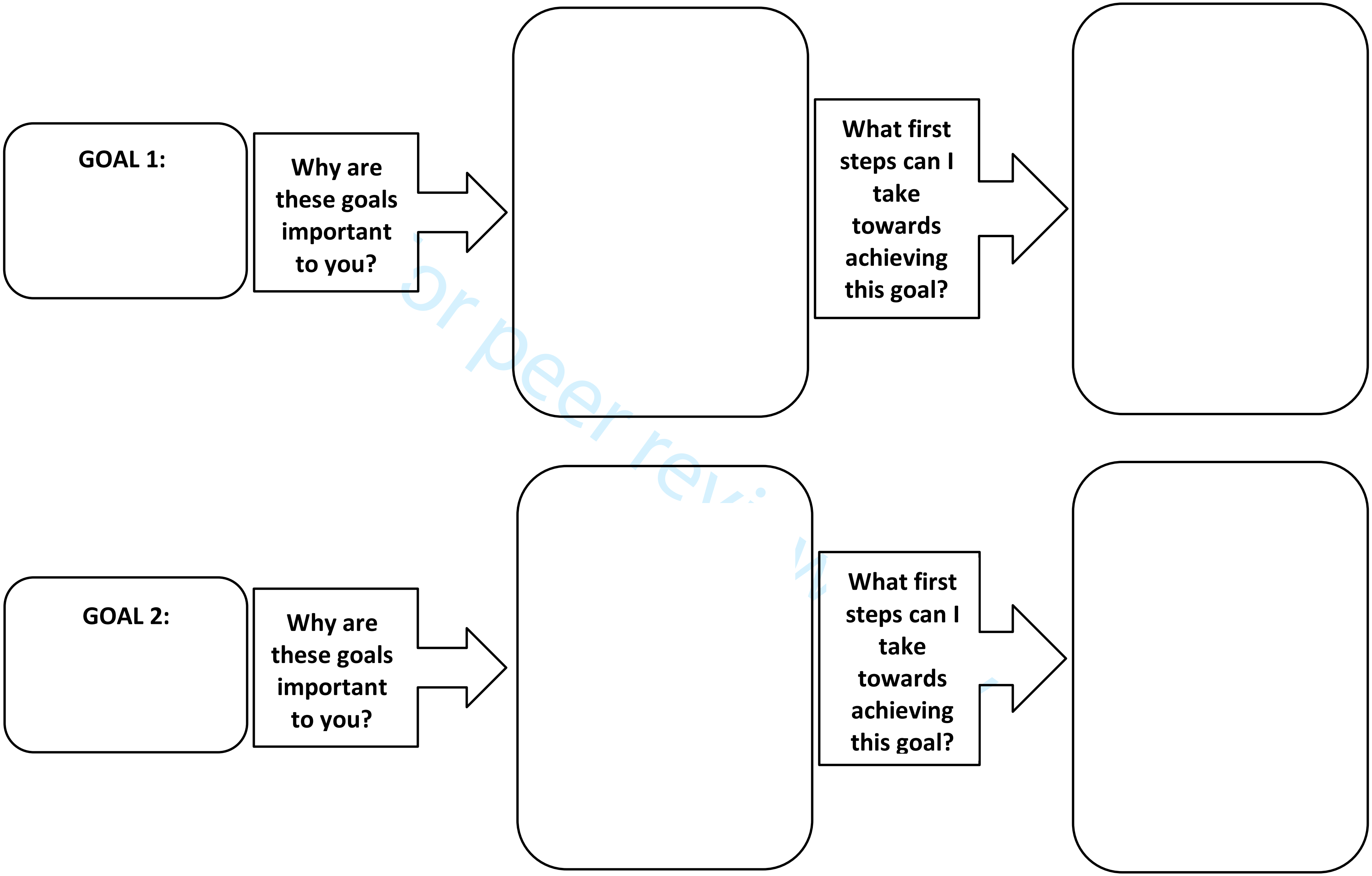

For peer review only - http://bmjopen.bmj.com/site/about/guidelines.xhtml

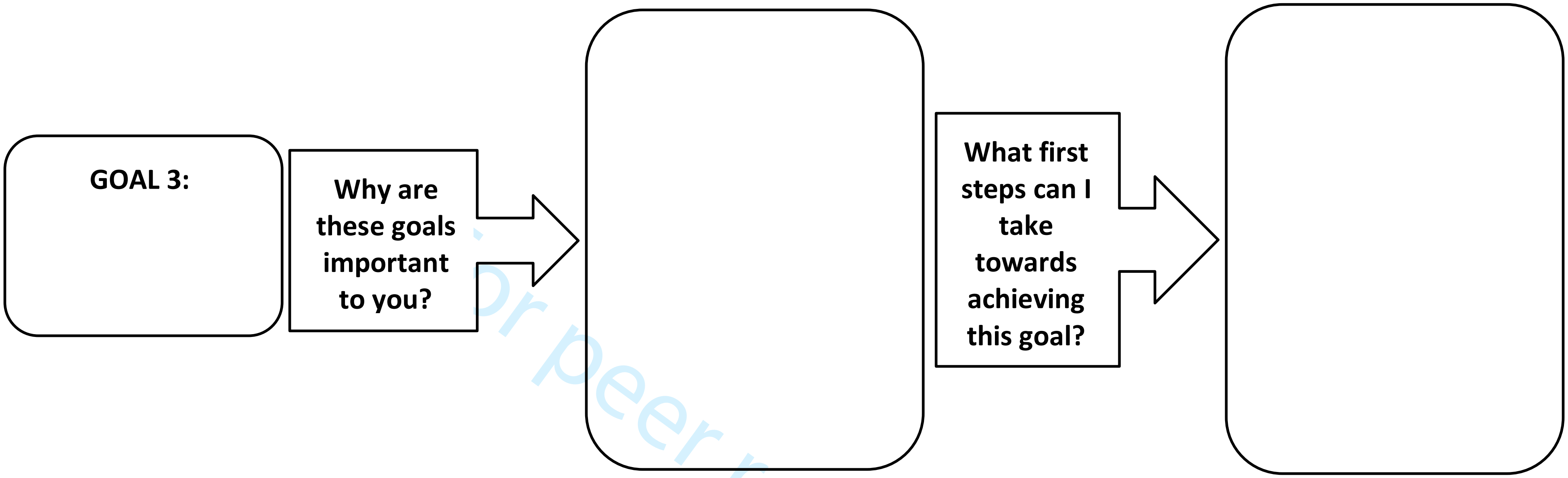

Thank you for completing this goal-setting form. Please take it with you to your care plan appointment. Your GP will discuss it with you and together you will make specific plans to achieve your goals.

For peer review only

University of East Anglia

GoalPlan Study

GAS-Light verbal scoring system

**Goal 1:**			Tick
**At Baseline**	With respect to this goal do they have?	Some attainment	☐
		No attainment (as bad as they could be)	☐
**At 6 month review:** Was the goal attained?	**Yes**	A lot more	☐
		A little more	☐
		As expected	☐
	**No**	Partially attained	☐
		No change	☐
		Got worse	☐

For peer review only

PTO for further goals

For peer review only - http://bmjopen.bmj.com/site/about/guidelines.xhtml

**Goal 2:**			Tick
**At Baseline**	With respect to this goal do they have?	Some attainment	☐
		No attainment<br>(as bad as they could be)	☐
**At 6 month review:**<br><br>Was the goal attained?	**Yes**	A lot more	☐
		A little more	☐
		As expected	☐
	**No**	Partially attained	☐
		No change	☐
		Got worse	☐

For peer review only

PTO for further goals

For peer review only - http://bmjopen.bmj.com/site/about/guidelines.xhtml

**Goal 3:**			Tick
**At Baseline**	With respect to this goal do they have?	Some attainment	☐
		No attainment<br>(as bad as they could be)	☐
**At 6 month review:**<br><br>Was the goal attained?	**Yes**	A lot more	☐
		A little more	☐
		As expected	☐
	**No**	Partially attained	☐
		No change	☐
		Got worse	☐

For peer review only

For peer review only - http://bmjopen.bmj.com/site/about/guidelines.xhtml

CONSORT 2010 checklist of information to include when reporting a pilot or feasibility trial*

Section/Topic	Item No	Checklist item	Reported on page No
**Title and abstract**			
	1a	Identification as a pilot or feasibility randomised trial in the title	1
	1b	Structured summary of pilot trial design, methods, results, and conclusions (for specific guidance see CONSORT abstract extension for pilot trials)	2
**Introduction**			
Background and objectives	2a	Scientific background and explanation of rationale for future definitive trial, and reasons for randomised pilot trial	4
	2b	Specific objectives or research questions for pilot trial	4
**Methods**			
Trial design	3a	Description of pilot trial design (such as parallel, factorial) including allocation ratio	5
	3b	Important changes to methods after pilot trial commencement (such as eligibility criteria), with reasons	5
Participants	4a	Eligibility criteria for participants	5
	4b	Settings and locations where the data were collected	5
	4c	How participants were identified and consented	5
Interventions	5	The interventions for each group with sufficient details to allow replication, including how and when they were actually administered	5-6
Outcomes	6a	Completely defined prespecified assessments or measurements to address each pilot trial objective specified in 2b, including how and when they were assessed	6
	6b	Any changes to pilot trial assessments or measurements after the pilot trial commenced, with reasons	5
	6c	If applicable, prespecified criteria used to judge whether, or how, to proceed with future definitive trial	NA
Sample size	7a	Rationale for numbers in the pilot trial	5
	7b	When applicable, explanation of any interim analyses and stopping guidelines	NA
Randomisation:			
Sequence generation	8a	Method used to generate the random allocation sequence	5
	8b	Type of randomisation(s); details of any restriction (such as blocking and block size)	5
Allocation concealment mechanism	9	Mechanism used to implement the random allocation sequence (such as sequentially numbered containers), describing any steps taken to conceal the sequence until interventions were assigned	5

For peer review only

Implementation	10	Who generated the random allocation sequence, who enrolled participants, and who assigned participants to interventions	5
Blinding	11a	If done, who was blinded after assignment to interventions (for example, participants, care providers, those assessing outcomes) and how	NA
	11b	If relevant, description of the similarity of interventions	NA
Statistical methods	12	Methods used to address each pilot trial objective whether qualitative or quantitative	6
**Results**			
Participant flow (a diagram is strongly recommended)	13a	For each group, the numbers of participants who were approached and/or assessed for eligibility, randomly assigned, received intended treatment, and were assessed for each objective	Figure 1
	13b	For each group, losses and exclusions after randomisation, together with reasons	Figure 1
Recruitment	14a	Dates defining the periods of recruitment and follow-up	6
	14b	Why the pilot trial ended or was stopped	NA
Baseline data	15	A table showing baseline demographic and clinical characteristics for each group	Table 3
Numbers analysed	16	For each objective, number of participants (denominator) included in each analysis. If relevant, these numbers should be by randomised group	Tables 4, 5 and 7
Outcomes and estimation	17	For each objective, results including expressions of uncertainty (such as 95% confidence interval) for any estimates. If relevant, these results should be by randomised group	Table 7
Ancillary analyses	18	Results of any other analyses performed that could be used to inform the future definitive trial	NA
Harms	19	All important harms or unintended effects in each group (for specific guidance see CONSORT for harms)	9
	19a	If relevant, other important unintended consequences	NA
**Discussion**			
Limitations	20	Pilot trial limitations, addressing sources of potential bias and remaining uncertainty about feasibility	11-12
Generalisability	21	Generalisability (applicability) of pilot trial methods and findings to future definitive trial and other studies	11-12
Interpretation	22	Interpretation consistent with pilot trial objectives and findings, balancing potential benefits and harms, and considering other relevant evidence	11-12
	22a	Implications for progression from pilot to future definitive trial, including any proposed amendments	11-12
**Other information**			
Registration	23	Registration number for pilot trial and name of trial registry	1
Protocol	24	Where the pilot trial protocol can be accessed, if available	1
Funding	25	Sources of funding and other support (such as supply of drugs), role of funders	13
	26	Ethical approval or approval by research review committee, confirmed with reference number	5

For peer review only

Citation: Eldridge SM, Chan CL, Campbell MJ, Bond CM, Hopewell S, Thabane L, et al. CONSORT 2010 statement: extension to randomised pilot and feasibility trials. BMJ. 2016;355.
*We strongly recommend reading this statement in conjunction with the CONSORT 2010, extension to randomised pilot and feasibility trials, Explanation and Elaboration for important clarifications on all the items. If relevant, we also recommend reading CONSORT extensions for cluster randomised trials, non-inferiority and equivalence trials, non-pharmacological treatments, herbal interventions, and pragmatic trials. Additional extensions are forthcoming: for those and for up to date references relevant to this checklist, see www.consort-statement.org.

For peer review only

CONSORT 2010 checklist of information to include when reporting a pilot or feasibility randomized trial in a journal or conference abstract

Item	Description	Reported on line number
Title	Identification of study as randomised pilot or feasibility trial	1
Authors *	Contact details for the corresponding author	1
Trial design	Description of pilot trial design (eg, parallel, cluster)	2
Methods		
Participants	Eligibility criteria for participants and the settings where the pilot trial was conducted	2
Interventions	Interventions intended for each group	2
Objective	Specific objectives of the pilot trial	2
Outcome	Prespecified assessment or measurement to address the pilot trial objectives**	2
Randomization	How participants were allocated to interventions	2
Blinding (masking)	Whether or not participants, care givers, and those assessing the outcomes were blinded to group assignment	2
Results		
Numbers randomized	Number of participants screened and randomised to each group for the pilot trial objectives**	2
Recruitment	Trial status†	
Numbers analysed	Number of participants analysed in each group for the pilot objectives**	2
Outcome	Results for the pilot objectives, including any expressions of uncertainty**	2
Harms	Important adverse events or side effects	2
Conclusions	General interpretation of the results of pilot trial and their implications for the future definitive trial	2
Trial registration	Registration number for pilot trial and name of trial register	1
Funding	Source of funding for pilot trial	13

Citation: Eldridge SM, Chan CL, Campbell MJ, Bond CM, Hopewell S, Thabane L, et al. CONSORT 2010 statement: extension to randomised pilot and feasibility trials. BMJ. 2016;355.

**this item is specific to conference abstracts*

***Space permitting, list all pilot trial objectives and give the results for each. Otherwise, report those that are a priori agreed as the most important to the decision to proceed with the future definitive RCT.*

†For conference abstracts.

For peer review only
